# Author Correction: A scalable peptide-GPCR language for engineering multicellular communication

**DOI:** 10.1038/s41467-019-08545-y

**Published:** 2019-01-29

**Authors:** Sonja Billerbeck, James Brisbois, Neta Agmon, Miguel Jimenez, Jasmine Temple, Michael Shen, Jef D. Boeke, Virginia W. Cornish

**Affiliations:** 1 Department of Chemistry, Columbia University, New York, NY 10027 USA; 2 Institute for Systems Genetics and Department of Biochemistry and Molecular Pharmacology, NYU Langone Health, 430 East 29th Street, New York, NY 10016 USA; 3 Department of Systems Biology, Columbia University, New York, NY 10032 USA; 4 Present Address: The Koch Institute for Integrative Cancer Research, Massachusetts Institute of Technology, Cambridge, MA 02139 USA

Correction to: *Nature Communications*; 10.1038/s41467-018-07610-2; published online 29 Nov 2018.

The original version of this Article omitted a declaration from the Competing Interests statement, which should have included the following: ‘J.D.B. is a founder and Director of the following: Neochromosome, Inc., the Center of Excellence for Engineering Biology, and CDI Labs, Inc. and serves on the Scientific Advisory Board of the following: Modern Meadow, Inc., Recombinetics, Inc., and Sample6, Inc.’. This has now been corrected in both the PDF and HTML versions of the Article.

