# Supplementary material for: A scalable peptide-GPCR language for engineering multicellular communication
Source: Nat Commun. 2018 Nov 29;9:5057. doi: 10.1038/s41467-018-07610-2 (PMC6265332; doi:10.1038/s41467-018-07610-2)
Supplement: Supplementary file 1 — Supplementary Information [file 41467_2018_7610_MOESM1_ESM.pdf]

Supplementary Material

A scalable peptide/GPCR language for engineering multicellular communication

Billerbeck et al.

Supplementary Table 1 - Overview of GPCRs and peptide ligands. Ascomycete species used for genomic GPCR extraction, inferred peptide ligands (**Supplementary Table 2** lists peptide precursors used for inference of peptide ligands) and % identity of a given GPCR's amino acid sequence or a given motif stretch when compared to the *S. cerevisiae* Ste2 (see also **Supplementary Figure 1**). GPCRs are organized by % identity (full Ste2). For species codes labeled with a reference, the #1 peptide candidate has been postulated or tested before.

	Code	Species	Mature Peptide ligand	% Identity		
				Full Sc.Ste2	Res. 289-296	Res.228-248
1	Sc[2]	*Saccharomyces cerevisiae*	1-WHWLQLKPGQPMY	100	100	100
2	Sca[3]	*Saccharomyces castellii*	1-NWHWLRLDPGQPLY	67.68	100	100
3	Vp2[3]	*Vanderwaltozyma polyspora2*	1--WHWLRLRYGEPIY<br>2-PWHWLRLRYGEPIY	52.82	100	90.48
4	Vp1[3]	*Vanderwaltozyma polyspora1*	1-WHWLELDNGQPIY	50.79	100	85.71
5	Td	*Torulaspora delbrueckii*	1-GWMRLRLGQPL<br>2-GWMRLRLGQPM<br>3- GWMRLRIGQPL	49.8	100	95.24
6	Sk[4]	*Saccharomyces kluyveri*	1--WHWLSFSKGEPMY<br>2-PWHWLSFSKGEPMY	49.3	100	90.48
7	Kl[3]	*Kluyveromyces lactis*	1---WSWITLRPGQPIF<br>2-SPWSWITLRPGQPIF	48.93	75.0	85.71
8	Zr[3]	*Zygosaccharomyces rouxii*	1--HFIELDPGQPMF<br>2-AHFIELDPGQPMF	44.92	100	100
9	Zb	*Zygosaccharomyces bailii*	1--HLVRLSPGAAMF<br>2--PLVRLSPGAAMF<br>3-APLVRLSPGAAMF<br>4-AHLVRLSPGAAMF	44.34	100	100
10	Cg[5]	*Candida glabrata*	1-WHWVRLRKGQGLF<br>2-WHWVKIRKGQGLF	43.45	87.5	80.95
11	Ag	*Ashbya gossypii*	1-WFRLSLHHGQSM	41.04	87.5	80.95
12	Ss	*Scheffersomyces stipitis*	1--WHWTSYGVFEPG<br>2-PWHWTSYGVFEPG	36.22	75.0	66.67
13	Kp	*Komagataella (Pichia) pastoris*	1-FRWRNNEKNQPFG	35	87.5	66.67
14	Cgu[3]	*Candida (Pichia) guilliermondii*	1-KKNSRFLTYWFFQPIM	33.9	87.5	66.67
15	Cp[3]	*Candida parapsilosis*	1-KPHWTTYGYYEPQ	31.33	87.5	80.95
16	Cau	*Candida auris*	1-KWGWLRFFPGEPFV	30.87	87.5	71.43
17	Yl[3]	*Yarrowia lipolytica*	1-WRWFWLPGYGEPNW	30.8	87.5	38.10
18	Cl[3]	*Candida (Clavispora) lusitaniae*	1--KWKWIKFRNTDVIG<br>2---WGWIHFLNTDVIG<br>3-PKWKWIKFRNTDVIG	30.69	75.0	71.43
19	Ca[6]	*Candida albicans*	1-GFRLTNFGYFEPG	28.83	87.5	85.71
20	Ct[3]	*Candida tropicalis*	1-KFKFRLTRYGWFSPN	28.11	75.00	76.19
21	Cn	*Candida tenuis*	1-FSWNYRLKWQPIS	27.49	62.5	71.43
22	Le[3]	*Lodderomyces elongisporous*	1----WMWTRYGRFSPV<br>2-DPGWMWTRYGRFSPV	26.97	87.5	76.19
23	Gc	*Geotrichum candidum*	1--GDWGWFWYVPRPGDPAM<br>2-PGDWGWFWYVPRPGDPAM	26.76	87.5	57.14
24	Bm	*Baudoinia compniacensis*	1-GWIGRCGVPGSSC	26.56	87.5	42.86
25	So[3]	*Schizosaccharomyces octosporus*	1-----TYEDFLRVYKNWWSFQNPDRPDL<br>2-PACTTYEDFLRVYKNWWSFQNPDRPDL	26.04	87.5	28.57
26	Tm	*Tuber melanosporum*	1-WTPRPGRGAY	25.94	100	38.10
27	Ao[3]	*Aspergillus oryzae*	1-WCALPGQGC	24.67	87.5	33.33

28	Sp[7]	*Schizosaccharomyces pombe*	1--TYADFLRAYQSWNTFVNPDRPNL<br>2-KTYADFLRAYQSWNTFVNPDRPNL	23.75	87.5	28.57
29	Af[3]	*Aspergillus (Neosartorya) fischeri*	1-WCHLPGQGC	23.67	87.5	42.86
30	Pd	*Pseudogymnoascus destructans*	1---FCWRPGQPCG<br>2---FCQRPGQLCG<br>3-LEFGGLEKEQNS	23.56	87.5	28.57
31	Sj[3]	*Schizosaccharomyces japonicus*	1-----VSDRVKQMLSHWWNFRNPDTANL<br>2-PERRVSDRVKQMLSHWWNFRNPDTANL	23.3	87.5	28.57
32	Pb[8]	*Paracoccidioides brasiliensis*	1-WCTRPGQGC	22.9	87.5	28.57
33	Mg	*Mycosphaerella graminicola*	1-GNSFVGWCGAIGAPCA<br>2-------WCGAIGAPCA	22.44	100	42.86
34	Pr	*Penicillium chrysogenum*	1-WCGHIGQGC<br>2-KWCGHIGQGC	21.81	87.5	33.33
35	An[9]	*Aspergillus nidulans*	1-WCRFRGQVCG	21.73	87.5	38.10
36	Sn[3]	*Phaeosphaeria nodorum*	1-KYNGWRYRPYGLPVG	21.61	75.0	38.10
37	Hj	*Hypocrea jecorina*	1-WCYRIGEPCW<br>2-WCWILGGKCW	19.87	75.0	15.00
38	Bc[3]	*Botrytis cinerea*	1-WCGRPGQPC	19.54	75.0	28.57
39	Bb	*Beauvaria bassiana*	1-WCMRPGQPCW<br>2-WCMQTPKCW	19.23	50.0	15.00
40	Nc[10]	*Neurospora crassa*	1-QWCR---IHGQSCW<br>2-QVCNMRLHPKKVCW	18.94	50.0	20.00
41	She	*Sporothrix scheckii*	1---YCPLKGQSCW<br>2-QRYCPLKGQSCW	18	62.5	15.00
42	Mo[3]	*Magnaporthe oryzea*	1-QWCPRRGQPCW	17.56	50.0	20.00
43	Dh	*Dactylellina haptotyla*	1-WCVYNSCP	17.02	37.5	33.33
44	Fg[3]	*Fusarium graminearum*	1-WCWWKGQPCW<br>2-WCTWKGQPCW	16.8	50.0	30.00
45	Cc	*Capronia coronata*	1-GLSYWKGVNDGGSS	16.05	50.0	19.05

Supplementary Table 2 – Annotated pre-pro peptides used to infer mature peptide ligand sequences. Green: Potential secretion signal sequences. Bold: Potential Kex2 processing sites. Orange: Potential Ste13 processing sites. Underlined: Inferred mature peptide sequence. Grey highlighted residues: Repetitive extensions, yielding alternate peptide candidates. For Species codes labeled with a reference, #1 peptide candidates have been postulated or tested before.

	Code	Mature peptide ligand	Precursor
1	Af[3]	1-WCHLPGQGC	MRLLSLVLATFAATAVQADITPWCHLPGQGCYML**KR**AADASDEVRRSASAVA<br>EAVAEAFPQTPWCHLPGQGCAKA**KR**AAEAAAEEVKRSADAFAEAMAAFEKE
2	Ag	1-WFRLSLHHGQSM	MKTTHILSLATLAACAPVQPAPVQPTDLAAAANVPEKAVLGFFQLYNVGDVE<br>LLPVDDGAHSGILFVNRTLADVDYSSEHVVQKWFRLSLHHGQSM
3	An[9]	1-WCRFRGQVCG	MKLFFVSILLAALLATAVKAAPAAELQHRWCRFAGRICPPT**KR**TADALNFV**K**<br>**R**EAEAVAEPFKINRWCRFRGQVCGKA**KR**AAEAIGNVKLSAEAVADAMAFLDE<br>LTREEYAQLAKDFGHLKESDNSDG
4	Ao[3]	1-WCALPGQGC	MKLISVVVAALAATSVQAGVLQKWCSLPAQGCYML**KR**AADASGDVRRSAEAL<br>SEAMPDAEALAKWCALPGQGCLKA**KR**AAEAVEEARRSADALADAMADLGEY
5	Bb	1-WCMRPGQPCW<br>2-WCMQT-PKCW	MKLSLVMLATAATTVIAAPRPWCMRPGQPCWKLKRAVDALGEPAPSPVEPLD<br>ADNIGLFASGAHDRLLHLASSDAANVDDEGAFE**KR**WCMQTPKCWKLLADEDG<br>ELS**KR**WCMRPGQPCW**KR**SVDEHGDLA**KR**WCMRPGQPCWKAKRAAESVLNAGQ<br>EDGDAQEQDCGDDGECSVAKRHLDGLHHVARAIVEAF
6	Bc[3]	1-WCGRPGQPC	MKFTNAIALAILAATATAVAVPEPWCGRPGQPC**KR**EAVAVAAPVAEPWCGRP<br>GQPC**KR**TPEAEAWCGRPGQPC**KR**DAEPWCGRPGQPC**KR**EALPEAWCGRPGQP<br>C**KR**TPLAEAEAEAWCGRPGQPCRKN**KR**AAEAVAEAFAEPWCGRPGQPC**KR**DA<br>EADVSEAAI**KR**CNMVGGACFEA**KR**LARDLAEATAETVEDSDLFLRSLNIETR<br>EVSEVVAREAEAWCGRPGQPC**KR**DAEAWCGRPGQPC**KR**EALAEAEAWCGRPG<br>QPC**KR**EALAEAEAWCGRPGQPC**KR**TAEPWCGRPGQPCKE**KR**EADPEAEAWCG<br>RPGQPCRAV**KR**AAEAIAEALAEPTAEAWCGRPGQPC**KR**EALAEAEANAEAWC<br>GRPGQPCRKA**KR**DAFALAYAADVALAQL
7	Bm	1-GWIGRCGVPGSSC	MKFSIVAVAAVAAQAAAVSGSTSAVFKDGVGACNVPGQKCHTVKNAARDILN<br>AINKPTDVDDQQSYFCDIQGSAGCNQLHGSVDKLQQAAIKAYHTVA**AR**EAEA<br>EAEAEANPGYGWIGRCGVPGSSCNK**KR**EADPGYGWIGRCGVPGSSCNK**KR**DE<br>DAAAREHWLAQREAGGWIGRCGVPGSSCNKKREEEVEVLRREAEAGGWIGRC<br>GVPGSSCNKARDANPGGWIGRCGVPGSSCNKKREAGGWIGRCGVPGSSCNKA<br>RDAEDDQKIQQMQDAIRAFNPEIEKAECNQDGQPCDLIKTAAQALHNNTRRE<br>AEAGGWIGRCGVPGSSCNKNKRALAFCQSGENCTGPAYAHLQSQDATADKAE<br>KDCHGPNGACTIAARALAELEQAVDAALLDADA
8	Ca[6]	1-GFRLTNFGYFEPG	MKFSLTLLTATIATIVAAAPAQYTGQAIDSNQVVEIPESAVEAYFPIDDELT<br>PVFGEIDNKPVILIVNGTTLTSGANNE**KR**EAKSKGGFRLTNFGYFEPG**KR**DA<br>NADAGFRLTNFGYFEPG**KR**DANAEAGFRLTNFGYFEPGK
9	Cau	1-KWGWLRFFPGEPFV	MKFSITAIIAATGSLVAAAPTPSSTDAPSFSEVPSSVESSFGVPTEAIIGQF<br>SFDADEYPLLTVYEDRRYIILLNSTIMEEAYASLNSGNE**KR**DAEAEAKWGWL<br>RFFPGEPFV**KR**DAEADAEAKWGWLRFFPGEPFVKRDAEADAEAKWGWLRFFP<br>GEPFVKRDAEADAEAKWGWLRFFPGEPFVKRDADAEAKWGWLRFYPGEPFVK<br>REVEADLEG
10	Cc	1-GLSYWKGVNDGGSS<br>2---SYWKGVNDGGSS	MHISSTTVTLVLTASFIQSALAFPVPAFLDVLRRDASPDPRLSYWKGVNDGG<br>SSKIKSRRWLSPIIEMLD**KR**EPGLSYWKGVNDGGSS**KR**EAAPEPDPGLSYWK<br>GVNDGGFS**KR**EAEPEPEPEPRLPYWKGVNDGGSS**KR**EAAPEPDPGLSYWKGV<br>NDGGSS**KR**EAAPEPEPEPEPGLSYWKGVNDGGSS**KR**GLSYWKGVNDGGSS**KR**<br>EAEPEPQPDALPALGLT
11	Cg[5]	1-WHWVRLRKGQGLF<br>2-WHWVKIRKGQGLF	MRFLRFISTVALLITGLATAQPVGEELGETVEVPSEAFIGYLDFGATNDVAI<br>LPISNKTNNGLLFVNTTLYNQATKGEKLSDFT**KR**DANPDAEAEAWHWVKIRK<br>GQGLF**RR**SADASPEAEAWHWVRLRKGQGLF**RR**SADASPEAEAWHWVRLRKGQ<br>GLF
12	Cgu[3]	1-KKNSRFLTYWFFQPIM	MKFSTAFVSTLFATYAAAAPLAAASDKIPVPFPKSAVNQIVTIDETNAPIYL<br>NNSGTITLFLVNTTVKEESPE**KR**ELGEVATGYEFNAAQYMKRESFPIENLVP<br>ESSLE**KR**EDKKNSRFLTYWFFQPIMKRGEEETSEVVKREAKKNSRFLTYWFF<br>QPIMKREEDIVAGDEMVKREAKKNSRFLTYWFFQPIMKREGGNEVEKRDAKK<br>NSRFLTYWFFQPIM
13	Cl[3]	1--KWKWIKFRNTDVIG<br>2---WGWIHFLNTDVIG<br>3-PKWKWIKFRNTDVIG	MKFSLAIIFSLAAAVVSAAPVAPESSSDFQIPEEAIISSQALGDDQLPLLLG<br>EGNATYFVLVNGTTLAEAYGITKRDAEAFDATYLGSSVA**KR**EANADAWGWIH<br>FLNTDVIGKRDAEPKWKWIHFRNTDVIGKRDASPKWKWIKFRNTDVIGKRDA<br>EADASPKWKWIKFRNTDVIGKRDAEADAAPKWKWIKFRNTDVIGKRDANAAP<br>KWRWINFRNTDVIGKREAQE

14	Cn	1-FSWNYRLKWQPIS	MRLSTILTLALTSKFVFSAPVEKVKREDGLDVPDEAIIAVYPIDEYKQPFYA<br>EADGQNYVVILNTTALGEADLA**KR**DADAFSWNYRLKWQPIS**KR**DADADADAD<br>AFSWNYRLKWQPIS**KR**DADADADADADAFSWNYRLKWQPIS
15	Cp[3]	1-KPHWTTYGYYEPQ	MKFSIAVLTAIAAALVASAPVASKEAEVPALPVDNVLERVVEAFFNGPSIDA<br>EIKDKTAADVKGVVGSQ**KR**EAEAKPHWTTYGYYEPQ**KR**DANAEAEAKPHWTT<br>YGYYEPQ**KR**DANAEAEAKPHWTTYGYYEPQK
16	Ct[2]	1-KFKFRLTRYGWFSPN	MKFSLALLTTVAAALVVAAPTQAPVEEAEVPTNETGLAIPDSAVCAIVPLDG<br>ELAPVFVELDDIPVLMIVNTTAVEEAYQAEEEAYEAEEGSSDVE**KR**DAAKFK<br>FRLTRYGWFSPN**KR**EEIDAEDIIDAEKRDAAKFKFRLTRYGWFSPN**KR**DIGD<br>EEDIVDAE**KR**DAAKFKFRLTRYGWFSPN**KR**ELAEEEETVDAE**KR**DAAKFKFR<br>LTRYGWFSPN**KR**EVAEENDIVE**KR**DAAKFKFRLTRYGWFSPN
17	Dh	1-WCVYNSCP<br>2-WCVYNSCPKT	MQLKHTITILSLLAPLLNALPVAEPEPTAAPEAKAGSGDVMLPRSWCIYNSC<br>PKN**KR**APEPVAEPVAIPEPTAAPEPVIPAHIEARGVEAVRRWCVYNSCPKTK<br>REAAPAPEPTAEPEPVIPAHIEARGEEYV**KR**WCVYNSCPKT**KR**AAEPIPEPT<br>AQPEPIIPDHVQAQGEEFV**KR**WCVYNSCPKT**KR**EAQPEPTAAPEPVIPDHIQ<br>ARGEEYI**KR**WCVYNSCPKT**KR**EAQPEPTAAAEAGIPAHIQARGEEYV**KR**WCV<br>YNSCPKT**KR**EAMPEPTAAPEPVIPDHIQARGEEFV**KR**WCVYNSCPKT**KR**EAA<br>PAPAPTAAPEPVIPAHIQARGEEYV**KR**WCVYNSCPKT**KR**EALPAPTAAPEPI<br>PAPEAEKMEPRSWCIYNSCPKYKRAAQPVPEPTAMPVA
18	Fg[3]	1-WCWWKGQPCW<br>2-WCTWKGQPCW	MKYSILTLAAVASTTLAVAVPAPQPDPVAEPMPWCTWKGQPCW**KE**KMARREA<br>QPEPEAVAAPEPDPVAEPMPWCTWKGQPCW**KE**KMARRAAQPEPEAVAAPEPD<br>PVAEPMPWCTWKGQPCW**KE**KMKMAKREAQPEPEAVAAPEPDPVAEPMPWCTW<br>KGQPCW**KE**KMAKRAAEAEAEPEPIPAPQPDPVAEAEPWCTWKGQPCW**KA**KMA<br>KRAAEAEAEAEPIPDPVAAPQPDPVAEPMPWCTWKGQPCW**KE**KMAKREAKPE<br>PWCWWKGQPCW**KAKR**DAAPEPWCWWKGQPCW**KA**KRNAAPEPMPEPANEPRWC<br>WWKGQPCWKSKSKRDASPEPWCWWKGQPCW**KAKR**DAGEALTVALHATRGVET<br>RSVAETEHLPRDAAHQAKRSIVELANVIALSARGSPEEYFKHLYLEEFFPEI<br>PHNATAKRDVKTLQED**KR**WCWWKGQPCW**KAKR**AAEAVLHAVDGSDGAGAPGG<br>PEEHFDTSHFNPQNFEAKRDLMAIKAAARSVVESLEG
19	Gc	1--GDWGWFWYVPRPGDPAM<br>2-PGDWGWFWYVPRPGDPAM	MRFSLATVYAFTVIGTVLGVPIASSEPTATTLSTVAAASATFSPGGDSPFTG<br>IKNFPDFASFPPFPPGFDTGLS**KR**SADASPGDWGWFWYVPRPGDPAMKKRDA<br>LAEANPEANPGDWGWFWYVPRPGDPAMK**KR**DALADANPDANPVE
20	Hj	1-WCYRIGEPCW<br>2-WCWILGGKCW	METKEKTVVPKSKSPLSIYFSLDRVSLHPSSLLISPSPSHLLSPSPHIAKLQ<br>TMKFLAAVTVFASAALAAPNPEPWCYRIGEPCWKL**KR**TAEAFNLAVRSHDLT<br>TRAQGEAIPDEVALSAIEGLDQLKKLILVSTEDPSSLLPPNATEPESKRDVE<br>VEED**KR**WCYRIGEPCWKA**KR**EAEAEAAAEEE**KR**WCYRIGEPCWKAKRTDEIS<br>EE**KR**WCWILGGKCWKT**KR**VAEAVLSATIEGDEKRSVEAEGNADE**KR**WCYRIG<br>EPCW**KAKR**DLETIQDVARSVIESMQ
21	Kl[3]	1---WSWITLRPGQPIF<br>2-SPWSWITLRPGQPIF	MKFSTILAASTALISVVMAAPVSTETDIDDLPISVPEEALIGFIDLTGDEVS<br>LLPVNNGTHTGILFLNTTIAEAAFADKDDLK**KR**EADASPWSWITLRPGQPIF<br>**KR**EANADANAEASPWSWITLRPGQPIF**KR**EANADANADASPWSWITLRPGQP<br>IF**KR**EANPEAEADAKPSAWSWITLRPGQPIF
22	Kp	1-FRWRNNEKNQPFG	MKSLILNIISVTLAITSTAASAPVESIFANQPDSSLTDTNDGVGVGMSTIKE<br>EDFGKHFVENQILDEAVIMSLKLRKGVNLFFLDDIGLATELIGNKIAQIEAI<br>DLSERLAQSWTNIRKNRLFG**KR**EAEAEAEAEAFRWRNNEKNQPFG**KR**EAEAE<br>AEAEAEAEAEAEAFRWRNNEKNQPFG**KR**EAEAEAEAEAEAEAEAFRWRNNEK<br>NQPFG**KR**EAEAEAEAEAFRWRNNEKNQPFG**KR**EADAEAEAEAEAFRWRNNEK<br>NQPFG**KR**EAEAEAEAEAFRWRNNEKNQPFG**KR**EAEAEAEAEAEAEAFRWRNN<br>EKNQPFG**KR**EAEAEAEAEAFRWRNNEKNQPFG**KR**EADAEAEAEAEAFRWRNN<br>EKNQPFG**KR**EASIDTGTDDGAYWSWRKNSVLERQ
23	Le[3]	1----WMWTRYGRFSPV<br>2-DPGWMWTRYGRFSPV	MKFSTAVLTAIAVTLVAAAPVDIDTNANAADNVIEATTSNEEAAIPETTEIA<br>LDNAEQITDEQIPSDCGLELGPETQIEGELPQEDGEEGYYVYIPDTENFANE<br>EEAAQYYQ**KR**SADPGWMWTRYGRFSPV**KR**DANAEAEAEANADPGWMWTRYGR<br>FSPV**KR**DANAEAEAEDKAEANADPGWMWTRYGRFSPV**KR**DANAEAEAEANAD<br>PGWMWTRYGRFSPV**KR**DANAEAEANADPGWMWTRYGRFSPV**KR**DANAEADPG<br>WMWTRYGRFSPV**KR**DANAEADANAEADPGWMWTRYGRFSPV**KR**DANAEADPG<br>WMWTRYGRFSPV
24	Mg	1-GNSFVGWCGAIGAPCA<br>2-------WCGAIGAPCA	MKLAVSTVLMVAVTLTQALAVADAEPKRRRGNSFVGWCGAIGAPCAKV**KR**DA<br>EAMPDPK**KR**RGNSFTGWCGAIGAPCARV**KR**SADAIAEAFAYPEADPK**KR**RGN<br>SFVGWCGAIGAPCAKA**KR**DIIEVGESVEEAVHDVYAREAEAEADPK**KR**RGNS<br>FVGWCGAIGAPCA**KR**DLFSEVETDVSAEDSEDEDAIYARDAAPEARRKKKAK<br>KP**KR**RGHRGNSFVGWCGALGAPCAKV**KR**DADAVAFAEAKKQRGNSFTGWCGA<br>IGAPCAKD**KR**EEHEILKTDVCEADDGECKALRNAYEAFHEIKARDAELEAEN<br>LASIDDDDELTKREVEVCNEPDGECDLAKRALDTIEAKLDAAIKAL

25	Mo[3]	1-QWCPRRGQPCW	MKTVSVITLILGAGAAANAAAIVNAETLEARSEDAATLEARQWCPRRGQPCW<br>KVKRAVDAFASAMHSNEARDVATTTSPSDGHLTARDLSHLPGGAAYNAKRSV<br>NALAALLASTQYDPEAFYNDLYLDRYFDPDTSVDAKAVDEKPDAEAKTEKRD<br>EEGGHLEARQWCPRRGQPCWKRDVEHDKRHCNSAGEACDVAKRAVGALLSAV<br>EDSGADLAKRQWCPRRGQPCWKRDNVFEPVALGRRDVSDAEADVLTKRQWCP<br>RRGQPCWKRSEISGLEARCYGPAGECTKAQRDLNAIHLAARDVLASLDFGRH<br>LSSRLLDHS
26	Nc[10]	1-QWCRI---HGQSCW<br>2-QVCNMRLHPKKVCW	MKFTLPLVIFAAVASATPVAQPNAEAEAQWCRIHGQSCWKVKRVADAFANAI<br>QGMGGLPPRDESGHQPAQVAKRQVDELAGIIALTQEDVNAYYDSLSLQEKFA<br>PSTEEEKKTEKVAKREAEAEAQWCRIHGQSCWKKREAEAQWCRIHGQSCWKR<br>DALPEAEPQWCRIHGQSCWKKRDAAPEAAPEAEANPQWCRIHGQSCWKAKRA<br>AEAVMTAIQSAEAESALLLRDTTFSPVDRVGKRDPQVCNMRLHPKKVCWKRD<br>ASPEAACNAPDGSCTKATRDLHAMYNVARAILTAHSDEN
27	Pb[8]		
28	Pd	1---FCWRPGQPCG<br>2---FCQRPGQLCG<br>3-LEFGGLEKEQNS	MKYLATLCVAALVAGVNSAAIAAAEPFCWRLGQPCDKVKRAAEAFAEAFDEP<br>IAEAEAFDEPIAEAEASAFCWRPGQICEKAKRAALALAHTVADANPEAEAFF<br>DKLAIDEAFPEPEAVADAEIADKVKREAEAEAFCWRPGQPCGKVKRAADAIA<br>SALAEPAPEPFCQRPGQLCGKVKRDAEAVAEAFCWRPGQPCGKAKREANALA<br>EAAAEALEFGGLEKEQNSKRIFRPPHYTTTAIFPTDPRLFHHFHEEQPYDCR<br>KVDPNCVTVEA
29	Pr	1--WCGHIGQGC<br>2-KWCGHIGQGC	MKFTSVVVAVIAAGTVQAAALAPSETLPKWCGHIGQGCKRTTDASLDVKRSA<br>DALAEAMAGGLPLVLQKWCGHIGQGCYKAKRAADAVDEVKRTSDALARAFAA<br>LEEEDDE
30	Sc[2]	1-WHWLQLKPGQPMY	MRFPSIFTAVLFAASSALAAPVNTTTEDETAQIPAEAVIGYLDLEGDFDVAV<br>LPFSNSTNNGLLFINTTIASIAAKEEGVSLDKREAEAWHWLQLKPGQPMYKR<br>EAEAEAWHWLQLKPGQPMYKREADAEAWHWLQLKPGQPMY
31	Sca[3]	1-NWHWLRLDPGQPLY	MKLSALLSTVALASTSFAAPIDTTASNENLNSTDIPAEAVIGYLDLGSDSDV<br>AMLPFQNSTSNGLLFVNTTIVQQAAQENDDSVGLAKREANAEAGWHWLRLDP<br>GQPLYKREADADAEANWHWLRLDPGQPLYKREAEADAEANWHWLRLDPGQPL<br>YKREADADAEANWHWLRLDPGQPLYKREADADAEANWHWLRLDPGQPLY
32	She	1---YCPLKGQSCW<br>2-QRYCPLKGQSCW	MKTAAVFTILAVGASAAAVAEAEAYCQSVGQSCYQVKRAAEAFAEAIADLGA<br>PEAGISRRSLSFGGVHNNAIRAIDGLASIVASTQYNPRSFYSDLSLESHFPV<br>PVEEPVTKREAEADADADAQRYCPLPGQPCWKNKREAEAAADAEAQKYCPLK<br>GQSCWKARRAAEAVINAIEGGSVQKREAEADAEAQKYCPLKGQSCWKRNVGT<br>RCYAPGGACANASRDLHAIYNAARSVIESLPKAE
33	Sj[3]	1-----<br>VSDRVKQMLSHWWNFRNPDTANL<br>2-<br>PERRVSDRVKQMLSHWWNFRNPD<br>TANL	MKFSAIFILSLFASAFAAPVPSSDAVEAAAPIIPELLSTEQVVLEGRVSDRV<br>KQMLSHWWNFRNPDTANLKRSEPERRVSDRVKQMLSHWWNFRNPDTANLKRS<br>EPERRVSDRVKQMLSHWWNFRNPDTANLKRSEPERRVSDRVKQMLSHWWNFR<br>NPDTANLKKRALTDAQEEEAESEMDLLSYLLYSNDTSIAASGLNATEMVETI<br>LKDYE
34	Sk[4]	1--WHWLSFSKGEPMY<br>2-PWHWLSFSKGEPMY	MKLFTTLSASLIFIHSLGSTRAAPVTGDESSVEIPEESLIGFLDLAGDDISV<br>FPVSNETHYGLMLVNSTIVNLARSESANFKGKREADAEPWHWLSFSKGEPMY<br>KREADAEPWHWLSFSKGEPMY
35	Sn[3]	1-KYNGWRYRPYGLPVG<br>2- GWRYRPYGLPVG	MRFNAVIAACILAVTVSGAALPTEDAAITDAATITTTEAEITEAEIIKAAPE<br>EDDFFDDDEQFEKRDAASWKYNGWRYRPYGLPVGKRDADAEAGWRYRPYGLP<br>VGKREAAPEADAEAKYNGWRYRPYGLPVGKREAEAKYNGWRYRPYGLPVGKR<br>EAEADASAEARYNGWRYRPYGLPVGR
36	So[3]	1-----<br>TYEDFLRVYKNWWSFQNPDRPDL<br>2-<br>PACTTYEDFLRVYKNWWSFQNPD<br>RPDL	MKFFSLVALLFALASAAPIPATSKDSGVSPLDQLPSKTYEDFLRVYKNWQTF<br>QNPDRPDLKKRDVPELPSKTYEDFLRVYKNWWSFQNPDRPDLKKRDVEELPA<br>KTYEDFLRVYQNWETFQNPDRPDLKKRDVPELPSKTYEDFLRVYKNWWSFQN<br>PDRPDLKKRDVEELPAKTYEDFERVYQNWETFQNPDRPDLKKRDVPELPSKT<br>YEDFLRVYKNWWSFQNPDRPDLKKRDVPELPSKTYEDFLRVYKNWWSFQNPD<br>RPDLKKRDVEEPVLKTEKDKEDYYHFLEFYVMNVPFNSTVAQTNISSHFD
37	Sp[7]	1--<br>TYADFLRAYQSWNTFVNPDRPNL<br>2-<br>KTYADFLRAYQSWNTFVNPDRPN<br>L	MKITAVIALLFSLAAASPIPVADPGVVSVSKSYADFLRVYQSWNTFANPDRP<br>NLKKREFEAAPAKTYADFLRAYQSWNTFVNPDRPNLKKREFEAAPEKSYADF<br>LRAYHSWNTFVNPDRPNLKKREFEAAPAKTYADFLRAYQSWNTFVNPDRPNL<br>KKRTEEDEENEEEDEEYYRFLQFYIMTVPENSTITDVNITAKFES
38	Ss	1--WHWTSYGVFEPG<br>2-PWHWTSYGVFEPG	MHLRSTAILSAVVFTSVALSAPTSGQNIDIDFPDESIAGAIPLSYDLVPIIG<br>SYQGQNVILIVNSTIAAASEAAASEGKSKRDANAWHWTSYGVFEPGKRDANA<br>NAAPWHWTSYGVFEPGKRDANADAAPWHWTSYGVFEPGK
39	Td	1-GWMRLRLGQPL-	MKFFNTILSTTLFTYVALAAPVESDPVNIPSEAILGYMDFTEDQDVGVVAYT<br>NSTFSGLIFFNSSIIETKDLTKRDAEAGWMRLRLGQPLKKRDADADADAGWM<br>RLSPGKPMKKREADADAEAGWMRLRIGQPL

40	Tm	1-WTPRPGRGAY	MKVTILFLATLLSAALSEPIPWEVNGNRGVY**RR**EPEAEAEAWHPRAGDPMAI<br>WQ**KR**NAEPYPEAEPEAIPWTPRPGRGAY**RR**HARPWTPRPGRGAY**RR**SAEAWH<br>PRAGPPAYTLS**KR**DAAPEPVRFQPIGSFYKE
41	Vp1[3]	1-WHWLELDNGQPIY	MKLTNVLSAVALASTALAAPVAKDATNTTDASSVQIPAEAVIGYLDLEQSND<br>VAMLQFSNSTNNGILFVNSTILKAAYAEANANSNSNT**KR**EAKADAWHWLELD<br>NGQPIY**KR**EANAEAKPWHWLELDNGQPIY**KR**EAKAEAKADAWHWLELDNGQP<br>IY**KR**EAKAEAKADAWHWLELDNGQPIY**KR**EAEAKAGAWHWLELDNGQPIY
42	Vp2[3]	1--WHWLRLRYGEPIY<br>2-PWHWLRLRYGEPIY	MKFSTVLSTVALAATAVSAAPISRASNETVESVESGLNVPAEAVLGYLDFGE<br>KDDVAMLPFSNGTSNGLLFVNTTIYDAAFADSDDESASLA**KR**DAEAWHWLRL<br>RYGEPIY**KR**EDSEGVEKREAAAEPWHWLRLRYGEPIY**KR**EDSESVEKREAAA<br>EPWHWLRLRYGEPIY**KR**EDSESVEKREANADADAWHWLRLRYGEPIY
43	Yl[3]	1-WRWFWLPGYGEPNW	MKFSTIALAAVACLVSAAPAAPVGTGSHGPQSIPEEAIVGGLQGTENEIFVF<br>FNDDESGKQGIAIIDAKKAQEAGFMDPQPDSEVAAGNA**KR**EASPEAWRWFWL<br>PGYGEPNW**KR**DAMPADMDKE**KR**EANPEAWRWFWLPGYGEPNW**KR**DAMPADMD<br>KE**KR**EANPEAWRWFWLPGYGEPNW**KR**DAMPADMDKE**KR**EANPEAWRWFWLPG<br>YGEPNW
44	Zb	1--HLVRLSPGAAMF<br>2--PLVRLSPGAAMF<br>3-APLVRLSPGAAMF<br>4-AHLVRLSPGAAMF	MRFSITLCSTLCALTVAAAPIEEY**KR**APVAEAEAAHLVRLSPGAAMF**KR**EAD<br>ADAEAEAAHLVRLSPGAAMF**KR**EAEAEAEAEAAHLVRLSPGAAMF**KR**EAEAD<br>ADAEAEAAPLVRLSPGAAMF**KR**EADADAEAEAAHLVRLSPGAAMF**KR**EAEAE<br>AEAEAAHLVRLSPGAAMF**KR**EAEADADAEAEAAHLVRLSPGAAMF**KR**EAEAE<br>AAHLVRLSPGAAMF**KR**EAEADAEAEAEAAPLVRLSPGAAMF**KR**KAEADAEAE<br>APPLVRLSPGAAMF**KR**EAEADADAEAEAAHLVRLSPGAAMF**KR**EAEADAEAE<br>AAHLVRLSPGAAMF**KR**EAEADADAEAEAAPLVRLSPGAAMF**KR**EAEADAEAE<br>AAHLVRLSPGAAMF**KR**EAEADADAEAEAAHLVRLSPGAAMF**KR**EAEADADAE<br>AGADST
45	Zr[3]	1--HFIELDPGQPMF<br>2-AHFIELDPGQPMF	MRLSIALGVTFGAVAGLTAPVEEV**KR**DADAHFIELDPGQPMF**KR**EAEAHFIE<br>LDPGQPMF**KR**EAEAEAHFIELDPGQPMF**KR**EAEAEAHFIELDPGQPMF**KR**EA<br>EADAHFIELDPGQPMF**KR**DADAHFIELDPGQPMF**KR**EAEAEAHFVELDPGQP<br>MF**KR**EAEADAHFIELDPGQPMF**KR**GEIESAA

Supplementary Table 3 – Sequences of codon-optimized GPCR genes, expression cassette and genomic integration design (*STE2* locus and *STE3* locus). Codon-optimized GPCR genes were cloned into vector pRS416 under control of the constitutive TDH3 promoter and the Ste2 terminator. The first row shows the sequence of the generic GPCR expression cassette. The second row shows the *STE2* locus replaced by the generic expression cassette. Codon-optimized sequences of the indicated GPCRs have been reported previously[11].

<table>
<tr><th colspan="2">TDH3p-xy.Ste2-Ste2t expression cassette</th></tr>
<tr><td colspan="2">AGTTTATCATTATCAATACTGCCATTTCAAAGAATACGTAAATAATTAATAGTAGTGATTTTCCTAACTTTATTTAGT
CAAAAAATTAGCCTTTTAATTCTGCTGTAACCCGTACATGCCCAAAATAGGGGGCGGGTTACACAGAATATATAA
CATCGTAGGTGTCTGGGTGAACAGTTTATTCCTGGCATCCACTAAATATAATGGAGCCCGCTTTTTAAGCTGGCA
TCCAGAAAAAAAAAGAATCCCAGCACCAAAATATTGTTTTCTTCACCAACCATCAGTTCATAGGTCCATTCTCTTA
GCGCAACTACAGAGAACAGGGGCACAAACAGGCAAAAAACGGGCACAACCTCAATGGAGTGATGCAACCTGCC
TGGAGTAAATGATGACACAAGGCAATTGACCCACGCATGTATCTATCTCATTTTCTTACACCTTCTATTACCTTCT
GCTCTCTCTGATTTGGAAAAAGCTGAAAAAAAAGGTTGAAACCAGTTCCCTGAAATTATTCCCCTACTTGACTAAT
AAGTATATAAAGACGGTAGGTATTGATTGTAATTCTGTAAATCTATTTCTTAAACTTCTTAAATTCTACTTTTATAGT
TAGTCTTTTTTTTAGTTTTAAAACACCAAGAACTTAGTTTCGACGGATACTAGTAAA- ATG…xySte2…TAG -
CTCGAGACGGCTTTGAAAAAGTAATTTCGTGACCTTCGGTATAAGGTTACTACTAGATTCAGGTGCTCATCAGAT
GCACCACATTCTCTATAAAAAAAAATGGTATCTTTCTTATTTGATAATATTTAAACTCCTTTACATAATAAACATCTC
GTAAGTAGTGGTAGAAACCACCTTTGCTTTTACGAGTTCAAGCTTTTTTCTTGCCATGATCTAGAACTCTCAGGCA
ATATATACAGTTAATCTTTTTTTACTGGGTTGTAGTTCTAATGTATTGTTTCGAAAAATAGCAACCAGGCACA</td></tr>
<tr><th colspan="2">STE2 locus with integrated TDH3p-xy.Ste2-Ste2t expression cassette (100bp upstream and 100bp downstream, corresponds to Ste2 terminator)</th></tr>
<tr><td colspan="2">GTATCCTGCTTTGCAATGAAACAATAGTATCCGCTAAGAATTTAAGCAGGCCAACGTCCATACTGCTTAGGACCT
GTGCCTGGCAAGTCGCAGATTGAAG – AGTTT… TDH3p-xy.Ste2…TAG -
CTCGAGACGGCTTTGAAAAAGTAATTTCGTGACCTTCGGTATAAGGTTACTACTAGATTCAGGTGCTCATCAGAT
GCACCACATTCTCTATAAAAAAAAA</td></tr>
<tr><th colspan="2">STE3 locus with integrated TDH3p-xy.Ste2-Ste2t expression cassette (100bp upstream and 100bp downstream, corresponds to Ste2 terminator)</th></tr>
<tr><td colspan="2">CTATATTATTGTACCACATTGCCAGATTTATGAACTCTGGGTATGGGTGCTAATTTTCGTTAGAAGCGCTGGTACA
ATTTTCTCTGTCATTGTGACACTA – AGTTT …TDH3p-xy.Ste2…TAG -
CACAAGAGTGTCGCATTATATTTACTGGACTAGGAGTATTTTATTTTTACAGGACTAGGATTGAAATACTGCTTTTT
AGTGAATTGTGGCTCAAATAATG</td></tr>
<tr><th>Code</th><th>Codon-optimized GPCR DNA sequence</th></tr>
<tr><td>Af</td><td>ATGAACTCCACCTTCGACCCATGGACCCAAAACATTACTTTGACTCAATCCGACGGTACCACTGTCATC
TCCTCTTTGGCTTTGGCCGATGACTACTTGCACTACATGATTAGATTGGGTATCAACTACGGTGCCCAA
TTGGGTGCTTGTGCTGTTTTGTTGTTGGTTTTGTTATTGTTGACTAGACCAGAAAAGAGAGTTTCTTCTG
TCTTCGTTTTGAACGTCGCTGCTTTGTTGGCTAACATCATCAGATTGGGTTGTCAATTGTCCTACTTCTC
TACCGGTTTCGCTAGAATGTACGCCTTGTTGGCCGGTGACTTCTCCAGAGTCTCTCGTGGTGCTTACG
CCGGTCAAGTTATGGCCTCCGTCTTCTTCACCATTGTCTTCATTTGTGTTGAAGCTTCTTTGGTTTTGCA
AGTTCAAGTCGTCTGTTCTAACTTGAGAAGACAATACAGAATCTTGTTATTGGGTGCTTCCACTTTGGCT
GCCTTGGTTCCAATTGGTGTTCGTTTGACTTACTCCGTTTTAAACTGTATGGTTATTATGCACGCTGGTA
CTATGGACCACTTGGATTGGTTGGAATCTGCTACCAACATCGTTACTACCGTTTCTATTTGTTTCTTCTG
TGCTGTTTTCGTTGTCAAATTAGGTTTGGCTATCAAGATGAGAAAGCGTTTGGGTGTCAAACAATTCGG
TCCAATGAGAGTTATCTTCATCATGGGTTGTCAAACCATGACCATCCCAGCTATTTTCGCTATTTGTCAA
TACTTCTCTAGAATTCCAGAATTTTCTCATAACGTTTTGACTTTGGTTATCATCTCTTTGCCATTGTCTTC
TATCTGGGCCGGTTTTGCTTTGGTCCAAGCCAACTCTACCGCCAGATCTACCGAATCTAGACATCATTT
GTGGAACATTTTGTCTTCCGATGGTGCTACCAGAGACAAGCCATCCCAATGTGTTTCTTCTCCAATGAC
CTCTCCAACCACTACCTGTTACTCCGAACAATCCACCTCTAAGCCACAACAAGACCCAGAAAACGGTTT
TGGTATTTCTGTTGCCCACGATATTTCCATCCACTCTTTCAGAAAGGACGCCCACGGTGATATT</td></tr>
</table>

Ag	ATGGGTGAAGAGGTATCTAGCTTTGTGGAACAGTATTATGATCCAAACTATGATCCCAGTCAATCCATG<br>CTAACCTACATGTCAAAGTTCAGTAACGAGTCGACAATAAAGTTTGAGGACTTACAAGAGTATATTAATG<br>AAAACGTCATGTTGGGGGTATTTACTGGCGCAAAGATAGCGGCAGCAGCTCTGGCGTTGATAATCCTA<br>TGGATGGTGACTAAAAGGAAAAGGACACCCATTTACATCGTTAACCAGATATCACTCCTGCTTACAGTC<br>ATCCATGGCATTCTGGTGTTGTCTGGCTTGCTCGGGGGGTTTTCTTCTTCTATATTCACACTGACACTAT<br>TCCCTCAATGCGTGAATCGGAGTGATATTCGCCTGTTTGTCGCTACCAATATCTCCATGGTTTCGCTTAT<br>AGCCTCTATACAGGTTTCATTGGTTCTCCAAGTTCACGTAATCTTTCGAGCAGGCACTCACAGACGGTT<br>AGGCATCTTCTTAACTGCGGTTTCCGCTATAATAGGGTTCACAACCGTGTGCTTTTACCTGGTTTCTGCT<br>GTCCTTTCAGTGATGGCTGTATACCAGGATATCGATAACATCGGCGATACATTCTTTCTGAGCATTGCG<br>TACATTTGTATGGCCATATCTGTCAATTTCATTTTTTTGTTACTATCCGTTAAGCTGCTTCTTGCAATCAG<br>ATTAAGACGCTTCCTAGGTCTAAAACAATTTGATGGCTTACACATACTCTTCATTATGTCTACTCAGACA<br>ATTATATGTCCGAGTATTCTGTTCATACTGGCTTTCGCTTGCGAGAAAAATATAACAGATTCTTTGGTGT<br>ATATTGCGGTCTTACTCGTCTCACTGTCGCTACCACTGTCATCTGTGTGGGCAACAGCAGCCAACAACG<br>CAACAGTCCCACCTTTTTTGAACGCCCACTCTCTTACTTCTAGGTACAAAGCTGAATCCTGGTACACAG<br>ATTCAAAGAATGATGCAGGTAGTTTTAGCTCCTCAGAAAATTGTGGATCGGGATATCGACATGGACGCT<br>ATTCTAACAATGGGGGTAGTAGTCCACATCAATGTACGGGGGGGGATAATACCGTCATTGATATCGAAA<br>AATGTCAATATAGAGTGAACCCTACGCCACATACTAGTGGGCAATTCGCTTTCAATCAGGATTCATTGG<br>AAACTGAATTCTCGGAAGATACCGTCGTGCAAATTCGTACGCCCAATACTGAGGTTGAAGAGGAGGCC<br>AAAATATTCTGGGCAAGAGCCAGTATCACTCACGAAAATAGTTCTTCTGGCGTTGAGTGCGGTGCGCAT<br>GACATGCAAACCAACGTCTTCAAGACTCCTACAAGTCAAACCGGAAGTGATTGCAAC
An	ATGGCTACCCACAACCAAATCTCTGATCAATGTCAATGGTCTTACCCAGAAGTCTTCACCACTCAAGCT<br>GTCGAAGAACCAACCGCCGAACCAGCTTCTTACCACTTGCACTCTACCTTGACTATTATGGCTTCTAAC<br>TTCGACCCATGGAACCAAACCATTACCTTCAGATTGGAAGACGGTACTCCATTCGACATTTCTGTCGAC<br>TACTTGGACGGTATCTTGCAATACTCTATCAGAGCTTGTGTCAACTACGCTGCTCAATTGGGTGCTTCT<br>GTCATTTTGTTTGTTATCTTGGTCTTGTTGACTAGAGCCGAAAAAAGAGCTTCTTGTTTGTTCTGGTTAA<br>ACTCCTTAGCTTTGTTGTTGAACTTCGCCAGATTGTTGTGTGACGTCTTGTTCTTCACCGGTAACTTCGT<br>CAGAATTTACACTTTGATCTCCGCTGACGAATCTAGAGTTACTGCTTCCGACTTGGCTACTTCCATCGTC<br>GGTGCTATCATGACCGCTTTGTTGTTGACCACTATTGAAATTTCTTTGGTTTTGCAAGTCCAAGTCGTTT<br>GTTCTAACTTGAGAAGAATCTACAGAAGAGCCTTGTTGTGTGTTTCCGCCGTCGTTGCCACTGCTACCA<br>TTGCTATTAGATACTCCTTGTTGGCTGTCAACATTAGAGCTATTTTGGAATTCTCCGACCCAACTACTTA<br>CAACTGGTTGGAATCTTTAGCTACCGTCGCCTTGACCATCTCCATCTGTTACTTCTGTGTCATCTTCGTC<br>ACCAAGTTAGGTTTCGCTATTAGATTGAGAAGAAAGTTGGGTTTATCTGAATTGGGTCCAATGAAGGTC<br>GTCTTCATCATGGGTTGTCAAACCTTGGTCATCCCAGGTAAAGAACCTTGTCTTCTTTGATTCCACCAG<br>TCATTGTTTCTATTACTCACTACGTCTCCGACGTCCCAGAATTGCAAACTAACGTTTTGACTATCGTCGC<br>CTTGTCCTTGCCATTGTCCTCTATTTGGGCTGGTACCACCATTGACAAGCCAGTCACTCACTCTAACGT<br>TAGAAACTTGTGGCAAATCTTGTCCTTCTCTGGTTACAGACCAAAGCAATCTACCTACATTGCTACCACT<br>ACTACCGCTACTACCAACGCTAAGCAATGTACCCACTGTTACTCTGAATCTAGATTGTTGACTGAAAAG<br>GAATCTGGTCGTAACAACGACACTTCTTCTAAGTCTTCCTCCCAATACGGTATCGCTGTCGAACACGAT<br>ATTTCCGTTAGATCTGCTCGTCGTGAATCTTTTGACGTC
Ao	ATGGACTCTAAGTTCGACCCATACTCTCAAAACTTGACTTTCCACGCTGCTGACGGTACCCCATTTCAA<br>GTTCCAGTCATGACCTTGAACGACTTTTACCAATACTGTATTCAAATTTGTATCAACTACGGTGCTCAAT<br>TCGGTGCTTCCGTCATCATTTTCATTATCTTGTTGTTATTGACTAGACCAGACAAAAGAGCTTCTTCTGT<br>TTTCTTCTTAAACGGTGGTGCCTTGTTGTTGAACATGGGTAGATTGTTGTGTCACATGATTTACTTCACT<br>ACTGACTTCGTCAAGGCTTACCAATACTTCTCTTCTGATTACTCTAGAGCCCCAACCTCTGCCTACGCTA<br>ACTCCATTTTGGGTGTCGTCTTGACCACCTTGTTGTTGGTTTGTATCGAAACCTCCTTGGTTTTACAAGT<br>CCAAGTCGTCTGTGCTAACTTGAGACGTAGATACAGAACCGTCTTATTGTGTGTTTCTATCTTGGTCGC<br>CTTGATCCCAGTCGGTTTGAGATTGGGTTACATGGTTGAAAACTGTAAGACTATTGTTCAAACTGATACC<br>CCATTGTCTTTGGTTTGGTTGGAATCTGCTACTAACATCGTCATTACCATCTCCATCTGTTTCTTCTGTTC<br>TATCTTCATCATCAAGTTGGGTTTCGCCATTCACCAAAGAAGAAGATTGGGTGTCAGAGATTTCGGTCC<br>AATGAAGGTCATTTTCGTCATGGGTTGTCAAACTTTGACTGTTCCAGCTTTGTTGTCTATTTTGCAATAC<br>GCTGTCTCTGTCCCAGAATTGAACTCTAACATTATGACTTTGGTTACTATCTCTTTGCCATTGTCCTCCA<br>TTTGGGCTGGTGTTTCTTTGACCCGTTCTTCCTCCACCGAAAACTCTCCATCCAGAGGTGCTTTGTGGA<br>ACCGTTTGACCGACTCTACCGGTACCAGATCTAACCAAACCTCTTCCACCGACACCGCCGTCGCTATG<br>ACCTACCCATCTAACAAGTCTTCTACTGTCTGTTACGCCGATCAATCTTCTGTCAAGAGACAATACGATC<br>CAGAACAAGGTCACGGTATCTCTGTTGAACACGATGTTTCTGTCCACTCCTGTCAAAGATTG

Bb	ATGGATGGTTCTTCTGCTCCATCTTCTCCAACTCCAGATCCAACCTTCGACAGATTCGCCGGTAACGTC<br>ACTTTCTTCTTGGCTGACCACATCACCACTACCTCCGTTCCAATGCCAGTCTTGAACGCCTACTACGAC<br>GAATCCTTGTGTACTACCATGAACTACGGTGCTCAATTAGGTGCTTGTTTAGTTATGTTGGTTGTCGTTG<br>TTGCTTTGACCCCAGCTGCTAAGTTGGCTAGAAGACCAGCTTCTGCTTTGCATTTGGTTGGTTTGTTGT<br>TGTGTGCTGTTAGATCCGGTTTGTTGTTTGCTTACTTCGTCTCCCCAATCTCTCACTTTTACCAAGTTTG<br>GGCTGGTGACTTCTCTGCCGTTTCCAGAAGATACTGGGACGCTTCTTTGGCTGCCAACACTTTAGCTTT<br>CCCATTGGTTGTCGTCGTTGAAGCTGCTTTGATCAACCAAGCTTGGACCATGGTTGCTTTCTGGCCAAG<br>AGCCGCTAAGGCCGCTGCCTGTGCTTGTTCTGCTGTCATTGTCTTGTTGACTATTGGTACTAGATTGGC<br>CTACACTATCGTCCAAAACCACGCTATTGTTACTGCCGTCCCACCAGAACACTTCTTGTGGGCTATTCA<br>ATGGTCCGCTGTTATGGGTGCTGTTTCCATCTTCTGGTTTTGTGCCGTTTTCAACGTCAAGTTGGTCTG<br>TCACTTAGTCGCTAACAGAGGTATCTTGCCATCTATCTCTGTTGTTAACCCAATGGAAGTCTTGGTTATG<br>ACTAACGGTACCTTGATGATTATCCCATCTATCTTCGCTGGTTTGGAATGGGCTAAGTTCACCAACTTC<br>GAATCCGGTTCTTTGACTTTGACTTCCGTTATTATTATCTTGCCATTGGGTACTTTGGCTGCCCAACGTA<br>TTTCTGGTCAAGGTTCCCAAGGTTACCAAGCTGGTCACTTATTCCACGAACAACAACAACAACAAGCTC<br>GTACCCGTTCCGGTGCCTTCGGTTCCGCTTCTCAACAATCCCATCCAACTAACAAGGTTCCATCCTCTA<br>TTACCTTGTCTACCTCTGGTACTCCAATTACTCCACAAATCTCTGCCGGTTCCCGTCCAGAATTACCATT<br>GGTTGATAGATCCGAACGTTTGGACCCAATTGACTTGGAATTGGGTAGAATCGATGCTTTCAGAGGTTC<br>TTCCGACTTCTCTCCATCCACCGCTAGACCAAAGCGTATGCAACGTGATAACTTCGCC
Bc	Sequence reported[10]
Bm	ATGGCCTCAAACGGCTGGCAAAACAATGCAACATTTGATCCATATGCTCAGACGTTCGTGTTACTACAG<br>CCAGATGGTCTAACTCCATTCCCAGCGTTGCTAGGTGATGTTTTAGCTTTGAATACTGTCAGCGTTACC<br>CAAGGTATTATTTATGGCACACAAGTCGGTATCTCCGGCTTGCTTTTACTGATACTATTGATTATGACTA<br>AACCAGACAAGAGAAGAAGTTTGGTGTTCATCCTGAATAGTCTTTCTCTACTGTTGATCTTTGCCAGAAA<br>CGTGTTGAGTTGTGTGCAATTGACTACTATATTTTATAACTTTTATAACTGGGAGTTGCACTGGTACCCT<br>GAAAGCCCTGCATTATCAAGAGCTATGGATCTATCTGCCGCAACTGAAGTGTTAAATATACCAATAGAC<br>GTGGCCATCTTCTCATCCTTGGTAGTTCAAGTTCATATAGTTTGTTGCACGATACATACACTGGTGAGG<br>ACCTCAGCACTGTTATCTAGTGCCGCGGTTGGTCTGGCCGCTGTGGCTGTTAGATTTGCTCTGGCTGT<br>GGTTAATATCAAATACAGTATTTTTGGTATTAATACATTGACTGAACCCCAATTTAACTTAATAGTACACC<br>TTAAAAGGGTAAGTGATATACTGACAGTGGTTGCTATCGCATTTTTCTCTAGCATTTTCGTCGCTAAGTT<br>GGGAGTGGCGATTCACACTAGAAGAACGCTAAATTTAAAGAATTTCGGTGCTATTCAAATCATATTCATA<br>ATGGGATGTCAAACTATGTTGATTCCTTTAATATTTGTTATAGTGTCTTTCTATGCTTCTAGAGGATCTCA<br>AATTGGGAGCATGGTTCCTACAGTGGTTGCAACCTTTTTGCCCCTATCAGGTATGTGGGCTAGCGCTCA<br>AACGAATAACGAAAAAATGGGGAGGGCTGACCAACGTTTCCATCGTGCAGTCCCTGTGGGCGCGACT<br>GATTTCTCAGTGACTAAGGCTAGAAGCGCAAAAGCCAGTGACACTCTAGATACACTAATCGGTGACGA<br>C
Ca	Sequence reported[10]
Cau	ATGGAATTCACTGGTGACATCGTTTTGAAGTACACTTTGGGTGGTGAAGAATACTTGTCTACTTTCGAAC<br>AATTGGACTCTTCTGTTAACAGATCTTTGGAATTGGGTGTTGTTCACGGTATCGCTATCGCTTGTGGTGT<br>TTTGTTGATGGTTTTGGCTTGGGTTATCATCATCAAGAAGAAGAACCCAATCTTCGTTTTGAACCAATTA<br>ACTTTACTATTGATGGTTATCAAGTCTTCTTTATACTTGGCTTTCTTGTTCGGTCCATTGTCTTCTTTGAC<br>TTACAAGTTCACTAGAGTTTTGCCACACGACAAGTGGCACGCTTTCCACGTTTACATCGCTACTAACGT<br>TATCCACACTTTATTGATCGCTACTGTTGAAATGACTTTGGTCTTCCAAATCTACATCATTTTCAAGTCTC<br>CAGAAGTTAGACACTTGGGTTACATCTTGACTGGTGCTGCTTCTGCTTTGGCTCTAACTATCGTTGCTTT<br>GTACATCCACTCTACTGTTATCTCTGCTGTTCAATTAAAGGAACAATTGTTGATGCACGAAATCAAGATC<br>ACTAACTCTTGGGTTAACAACGTTCCAATCATTTTGTTCTCAGCTTCTTTGAACGTTGTTTGTATCATTTT<br>GATCGCTAAGTTAGCTTTGGCTATCAAGACTAGAAGATACTTAGGTTTGAAGCAATTCGACGGTTTGCA<br>CATCTTGATGATCACTTCTACTCAAACTTTCATCGTTCCATCTGTTTTGATGATCGTTAACTACAAGCAAT<br>CTTCTTCTTACTTGACTTTGTTGGCTAACATCTCTGTTATCTTGGTTGTCTGTAACTTGCCATTGTCTTCT<br>TTGTGGGCTGCTTCTGCTAACAATTCTTCTACTCCAACTTCTTCTGCTAACACTGTTTTCTCTAGATGGG<br>ACTCTAAGTTCTCTGACACTGAAACTATCGCTCACGAATTACCATTGATCCCAGGTAAGGCTGAAAAGT<br>TGCAATTGGTTTCTCCAATCACTGAAAAGGGTGACACTCACACTATGTGTGAATCTCACGGTGACCAAG<br>ACTTGATCGACAAGATGTTGGACGACATCGAAGGTGCTGTTATGACTACTGAATTCAACTTGAACAACA<br>GAACTGTT

Cc	ATGGCTGCTAGAATTATCCCAGCTTTGACCTTGACCGCCCCAACCTCTTACCCAACCGCCGGTGTTGG<br>TGGTTACTACTACGACACTGCTTTCGGTGTTCCAACCTACTCCTCTGCCGCTTTCAACCAAACCACCTG<br>GAGATTGTTGGATAACTGGGACCACATCAACGTCAACTACGCTTCTTCCGAAGGTTTGGCTGCTGGTTT<br>AGGTTGGGCTACCTTGATTTACTTGTTGGCTTTGACTCCATCCCACAAGAGAACTACTCCATTCCACTG<br>TTTCTTGTTGGTTGGTTTGATTTTCTTGTTGGGTCACTTGATGGTCAACATTATTGCCGCCTTGACCCCA<br>GGTTTGAACACCACCTCTGCTTACACTTACGTTACCTTGGATACCTCCTCTTCCGTCTGGCCACGTAAG<br>TACATCGCTGTCTACGCTGTCAACGCTGTCGCTTCTTGGTTCGCTTTCATTTTTGCCACTATCTGTTTGT<br>GGTTGCAAGCTAAAGGTTTAATGACCGGTATCAGAGTCCGTTTCATCATCGTCTACAAGATTATCTTGAT<br>GTACTTGATCGTTGCTGCTGTCATTGCTTTGGCTATCTGTATGGCTTTCAACATTCAACAAATCTTATAC<br>ATTGGTAAGCCAGTTGAATTGGCTGACGGTACCGCTTTGTTGAGATTGAGAAACGCTTACTTAATCACC<br>TACGCTATCTCTATTGGTTCTTTCTCCTTAGTTTCTATCTGTTCTATCATGGATATCATCTGGAGAAGACC<br>ATCTAGAGTCATTAAGGGTCACAACATTTTCGCTTCCGCTTTGAACTTAGTTGGTTTGTTGTGTGCTCAA<br>TCCTTCGTCGTCCCATGTGAATACAAGAGAGCCTTGGGTCAAGTCCCAGATTGTACTACTTTCGCCGAT<br>CACATTTTCCACACCGTTATCTTCTGTATTTTGCAAGTTATTCCAAACTCTTCTGGTGTTATGTTGCCAGA<br>AATCATGTTATTGCCATCTGTTTACGTCATTTTGCCATTGGGTTCCTTGTTCATGACTGTTAACTCCCCA<br>GAATCCGATGTCAACAAGACCTCTTTCCCACCAAAGTCCTCCCCAGGTCCATTCGACAGATCCCCAACT<br>TTGACCTCTGGTACCTTGCCAGGTTCTAGACCAGAATCCTACGTTTTGGATATGGCTTCTGACAAGAAC<br>TCCGGTAACAGAAAGTCTGTTTGTTCCCAATTCGACCGTGAATTGAACTTGATCGATTCTTTGGACACTT<br>TGTCTGGTCGTGAAGGTGATTCTATGTTGCACGCCCAATCCAACAACAACAACCAAACCAGAGAACAAG<br>ACAAGCAACCAAGAGCCGATACCACCCACGTTGGTTCTGAAAACATGGTC
Cg	Sequence reported[10]
Cgu	ATGAAGTCCTGCTCCATCGGTTTCGGTATCCCATTCATTAATGAACCAAACTTCGAAACTGTTTCTATTT<br>TGACCATGGACGTTTCTTTCATTGACGCTGACGTCAATCCTGACAATATCTTGTTGAACTTCACCATTCC<br>TGGTTACCAAAACGGTTTCTCTGTTCCAATGGTTGTTATTAACGAATTGCAAAAGTCTCAAATGAAATAC<br>GCTATTGTTTACGGTTGTGGTGTCGGTGCCTCCTTGATTTTGTTGTTTGTCGTCTGGATTTTGTGTTCTA<br>GAAAGACTCCATTGTTTATCATGAACAACATTCCATTAGTTTTGTACGTCATCTCCTCTTCTTTGAACTTG<br>GCTTACATTACCGGTCCATTGTCTTCTGTTTCCGTCTTCTTGACCGGTATCTTGACTTCTCACGATGCCA<br>TTAACGTCGTTTACGCTTCCAACGCTTTGCAAATGTTGTTGATCTTTTCTATCCAATCTACCATGGCCTA<br>CCACGTTTACGTTATGTTCAAATCTCCACAAATTAAATACTTGAGATACATGTTAGTCGGTTTCTTGGGT<br>TGTTTACAAATTGTCACCACCTGTTTATACATCAACTACAATGTTTTGTACTCTCGTAGAATGCACAAATT<br>GTACGAAACTGGTCAAACCTACCAAGATGGTACCGTTATGACTTTCGTTCCATTCATCTTGTTCCAATGT<br>TCTGTCAACTTCTCTTCTATTTTCTTGGTTTTGAAGTTGATTATGGCCATTAGAACCAGACGTTACTTGG<br>GTTTGCGTCAATTCGGTGGTTTTCATATTTTGATGATCGTTTCTTTACAAACTATGTTGGTCCCATCTATT<br>TTGGTTTTGGTTAACTACGCCGCTCATAAGGCTGTTCCTTCCAACTTGTTATCTTCCGTTTCTATGATGA<br>TCATTGTTTTGTCTTTACCAGCTTCTTCTATGTGGGCCGCTGCTGCTAACGCCTCTTCTGCCCCTTCCTC<br>CGCTGCTTCCTCCTTGTTCAGATACACCACTTCTGATTCCGATAGAACTTTGGAAACTAAATCTGACCAC<br>TTCATCATGAAGCATGAGTCCCACAACTCTTCTCCAAATTCCTCCCCATTGACTTTGGTTCAAAAGAGAA<br>TTTCTGATGCCACCTTAGAATTACCAAAAGAGTTAGAAGACTTGATCGACTCCACCTCCATC
Cl	ATGAACCCAGCTGACATCAACATCGAATACACCTTGGGTGATACTGCTTTCTCTTCCACTTTCGCTGATT<br>TCGAAGCTTGGAAAACTAGAAACACTCAATTCGCTATTGTCAACGGTGTCGCTTTGGCTTGTGGTATTA<br>TCTTGATGGTCGTTTCTTGGATTATTATTGTTAACAAGAGAGCTCCAATCTTCGCTATGAACCAAACTAT<br>GTTGGTTATCATGGTTATTAAGTCCGCTATGTACTTGAAGCATATCATGGGTCCATTGAACTCCTTGACC<br>TTCCGTTTCACCGGTTTAATGGAAGAATCCTGGGCTCCATACAACGTTTACGTCACTATTAACGTCTTGC<br>ATGTTTTGTTGGTCGCTGCTGTCGAATCCTCTTTGGTCTTCCAAATCCATGTTGTTTTCAAGTCTTCTAG<br>AGCCAGAGTTGCTGGTAGAGCCATTGTTTCTGCTATGTCCACTTTGGCCTTGTTGATCGTTTCTTTGTAC<br>TTGTACTCTACTGTTAGACATGCTCAAACTTTGCGTGCTGAATTATCTCATGGTGACACTACCACTGTTG<br>AACCATGGGTCGATAACGTTCCATTGATTTTGTTTTCCGCTTCTTTGAACGTTTTGTGTTTGTTGTTGGC<br>CTTGAAATTGGTTTTCGCTGTCAGAACCAGAAGACATTTAGGTTTAAGACAATTCGACTCTTTCCACATC<br>TTGATTATTATGGCCACTCAAACTTTCGTTATCCCATCCTCTTTGGTCATCGCTAACTACAGATACGCTT<br>CTTCCCCATTGTTGTCTTCCATTTCCATCATCGTCGCCGTCTGTAACTTGCCATTGTGTTCCTTGTGGGC<br>TTGTTCTAACAACAACTCTTCCTACCCAACTTCTTCTCAAAACACTATTTTGTCCAGATACGAAACTGAAA<br>CCTCTCAAGCTACTGACGCTTCCTCTACCACCTGTGCCGGTATTGCTGAAAAGGGTTTCGACAAGTCTC<br>CAGACTCTCCAACTTTCGGTGACCAAGACTCCGTCTCTATCTCCCATATCTTGGACTCTTTGGAAAAGG<br>ATGTTGAAGGTGTCACCACCCATAGATTGACT

Cn	ATGGACTCCTACTTGTTGAACCATCCAGGTGACATCTCTTTGAACTTCGCCTTGCCATTGTCCGATGAA<br>GTCTACACTATTACCTTCAACGACTTAGACTCTCAATCTTCTTTTTCCATTCAATACTTGGTCATCCACTC<br>TTGTGCCATTACCGTCTGTTTGACCTTGTTGGTTTTGTTGAACTTGTTCATCAGAAACAAGAAGACTCCA<br>GTCTTCGTTTTGAACCAAGTCATCTTGTTCTTCGCTATCGTCAGATCTTCTTTGTTCATCGGTTTTATGAA<br>GTCTCCATTGTCCACCATCACCGCCTCTTTCACCGGTATCATTTCTGATGACCAAAAACACTTCTACAAG<br>GTCTCCGTCGCTGCTAACGCCGCTTTGATCATTTTGGTCATGTTGATTCAAGTTTCTTTCACTTACCAAA<br>TCTACATTATTTTCAGATCCCCAGAAGTTAGAAAGTTCGGTGTCTTCATGACCTCCGCCTTGGGTGTCTT<br>GATGGCTGTTACCTTCGGTTTTTACGTTAACTCCGCTGTCGCTTCTACCAAGCAATACCAACACATCTTC<br>TACTCTACCGACCCATACATCATGGACTCTTGGGTCACTGGTTTGCCACCAATCTTGTACTCTGCTTCC<br>GTCATCGCTATGTCTTTGGTCTTGGTTTTGAAGTTGGTCGCTGCTGTCAGAACCAGAAGATACTTGGGT<br>TTGAAGCAATTCTCCTCCTACCACATCTTGTTGATTATGTTCACCCAAACCTTGTTCGTTCCAACCATCT<br>TGACCATCTTAGCTTACGCTTTCTACGGTTACAACGATATCTTGATCCATATTTCTACCACCATCACCGT<br>TGTCTTGTTGCCATTCACCTCCATTTGGGCTTCTATCGCCAACAACTCTAGATCCTTGATGTCTGCCGCT<br>TCCTTGTACTTCTCCGGTTCCAACTCCTCTTTGTCTGAATTGTCTTCTCCATCTCCATCTGATAACGACA<br>CTTTGAACGAAAACGTCTTCGCCTTTTTCCAGACAAGTTGCAAAAGATGAACTCTTCTGAAGCCGTTTC<br>TGCTGTCGACAAGGTCGTTGTTCACGACCACTTTGATACCATCTCCCAAAAGTCTATCCCACACGACAT<br>CTTGGAAATTTTGCAAGGTAACGAAGGTGGTCAAATGAAGGAACACATCTCTGTCTACTCTGATGACTC<br>TTTCTCCAAGACTACTCCACCAATTGTCGGTGGTAACTTGTTGATCACCAACACCGACATCGGTATGAA<br>G
Cp	ATGAACAAGATTGTCTCCAAGTTGTCTTCTTCTGACGTCATCGTTACCGTCACCATCCCAAACGAAGAA<br>GATGGTACTTACGAAGTCCCATTCTACGCTATTGACAACTACCACTACTCCCGTATGGAAAACGCTGTT<br>GTTTTAGGTGCTACCATTGGTGCTTGTTCTATGTTGTTGATCATGTTGATTGGTATTTTGTTCAAGAACTT<br>CCAAAGATTGAGAAAGTCTTTGTTGTTCAACATCAACTTCGCTATCTTATTGATGTTGATTTTGAGATCC<br>GCTTGTTACATCAACTACTTGATGAACAACTTGTCTTCCATTTCTTTCTTCTTCACCGGTATTTTCGATGA<br>TGAATCTTTCATGTCTTCCGACGCTGCCAACGCCTTCAAGGTTATCTTGGTTGCCTTGATTGAAGTTTCC<br>TTGACCTACCAAATTTACGTTATGTTCAAGACCCCAATGTTGAAGTCCTGGGGTATTTTCGCCTCTGTCT<br>TGGCCGGTGTTTTGGGTTTGGCTACTTTGGCTACCCAAATCTACACTACCGTTATGTCTCACGTTAACTT<br>CGTCAACGGTACCACCGGTTCTCCATCTCAAGTTACTTCCGCTTGGATGGACATGCCAACTATCTTATT<br>CTCCGTTTCTATTAACGTTTTGTCTATGTTCTTGGTTTGTAAGTTGGGTTTGGCCATCAGAACCAGACGT<br>TACTTGGGTTTAAAGCAATTCGACGCTTTCCACATTTTATTCATTATGTCCACTCAAACCATGATCATTCC<br>ATCCATCATCTTGTTCGTTCACTACTTCGATCAAAACGACTCTCAAACCACCTTGGTCAACATCTCTTTG<br>TTATTGGTCGTCATTTCCTTGCCATTGTCTTCTTTGTGGGCTCAAACTGCTAACAACGTTAGAAGAATTG<br>ACACTTCTCCATCCATGTCCTTCATCTCTAGAGAAGCTTCCAACAGATCTGGTAACGAAACCTTGCACT<br>CTGGTGCTACTATCTCTAAGTACAACACCTCCAACACCGTTAACACTACCCCAGGTACTTCTAAGGATG<br>ACTCTTTGTTCATCTTGGACAGATCCATTCCAGAACAAAGAATTGTCGACACTGGTTTGCCAAAGGACTT<br>GGAAAAGTTCATTAACAACGATTTTTACGAAGACGATGGTGGTATGATTGCCAGAGAAGTCACCATGTT<br>GAAGACCGCTCACAACAACCAA
Ct	ATGGACATCAACAACACCATCCAATCTTCCGGTGACATCATCATTACCTACACCATCCCAGGTATCGAA<br>GAACCATTCGAATTGCCATTCGAAGTTTTGAACCACTTCCAATCTGAACAATCCAAGAACTGTTTGGTCA<br>TGGGTGTTATGATCGGTTCTTGTTCCGTTTTGTTGATCTTCTTGGTCGGTATTTTGTTCAAAACCAACAA<br>ATTCTCTACTATTGGTAAGTCTAAGAACTTGTCTAAGAACTTCTTGTTCTACTTGAACTGTTTGATCACCT<br>TCATCGGTATCATTCGTGCTGCCTGTTTTTCTAACTACTTGTTGGGTCCATTGAACTCTGCTTCTTTCGC<br>TTTCACTGGTTGGTACAACGGTGAATCTTACGCTTCTTCCGAAGCTGCTAACGGTTTCAGAGTCATCTT<br>GTTCGCTTTGATTGAAACTTCTATGGTCTTCCAAGTTTTCGTTATGTTCAGAGGTGCTGGTATGAAAAAG<br>TTGGCTTACTCCGTTACCATTTTGTGTACCGCTTTGGCTTTGGTCGTTGTTGGTTTCCAAATTAACTCCG<br>CTGTCTTATCTCACAGAAGATTCGTCAACACCGTTAACGAAATTGGTGATACTGGTTTGTCCTCCATTTG<br>GTTGGACTTGCCAACCATCTTGTTCTCCGTCTCTGTCAACTTAATGTCTGTTTTGTTGATCGGTAAATTG<br>ATCATGGCTATTAAGACTAGAAGATACTTGGGTTTGAAACAATTCGATTCCTTCCACGTTTTGTTAATTT<br>GTTCCACTCAAACTTTGTTGGTCCCATCTTTAATCTTGTTCGTTCACTACTTCTTGTTCTTTAGAAACGCC<br>AACGTTATGTTGATTAACATTTCCATCTTGTTGATCGTCTTGATGTTGCCATTCTCTTCCTTGTGGGCTC<br>AAACCGCCAACACCACCCAATACATCAACTCTTCCCCATCCTTCTCTTTCATCTCTAGAGAACCATCTGC<br>TAACTCTACTTTGCACTCCTCTTCCGGTCACTACTCTGAAAAGTCCTACGGTATTAACAAATTGAACACC<br>CAAGGTTCTTCCCCAGCCACCTTAAAGGATGATCACAACTCCGTCATCTTGGAAGCTACCAACCCAATG<br>TCTGGTTTCGACGCCCAATTGCCACCAGACATTGCTAGATTCTTGCAAGATGACATCAGAATTGAACCA<br>TCTTCTACCCAAGATTTCGTTTCCACTGAAGTCACCTACAAGAAGGTC

Dh	ATGGACCACAACACCCAACACTTCAACAGACCTGAATACATTGAAATCCCAGTTCCACCATCTAAGGGT<br>TTCAACCCACACACCAACCCTGCTTTCTTCATCTACCCAGACGGTTCTAATATGACCTTTTGGTTCGGTC<br>AAATCGACGATTTCAGACGTGACCAATTATTCACTAACACCATCTTTTCCATTCAAATTGGTGCCGCTTT<br>GGTCATCTTATGTGTCATGTTTTGTGTTACCCACGCTGATAAGCGTAAAACCATTGTCTACTTGTTAAAC<br>GTTTCCAACTTGTTCGTTGTTATCATTAGAGGTGTTTTCTTTGTTCATTACTTCATGGGTGGTTTGGCCA<br>GAACCTATACCACTTTCACCTGGGATACTTCTGATGTTCAACAATCTGAGAAGGCTACTTCCATTGTCTC<br>CTCTATTTGTTCTTTGATTTTGATGATCGGTACTCAAATCTCCTTATTGTTGCAAGTCAGAATCTGTTACG<br>CTTTGAACCCAAGATCCAAGACCGCTATCTTGGTTACTTGTGGTTCTATTTCCGGTATTGCTACCACTGC<br>TTATTTATTGTTGGGTGCTTACACTATTCAATTGAGAGAAAAGCCACCAGACATGAAGTTCATGAAGTGG<br>GCTAAGCCAGTTGTTAACGCTTTGGTTGCCTTGTCCATTGTCTCCTTTTCTGGTATTTTCTCTTGGAGAA<br>TGTTCCAATCTGTCAGAAACAGAAGAAGAATGGGTTTCACTGGTATCGGTTCCTTGGAATCTTTGTTGG<br>CTTCTGGTTTCCAATGTTTAGTCTTCCCTGGTTTGGTTACTACCGCTTTGACCGTCGCCGGTTCCACTT<br>GGTATATCGCTGTTAACTTAACTACTCCATCTGACTTGACCGCTATTTACAACTGTTCCGCTTTTTTCGC<br>TTATGCTTTCTCCATTCCATTGTTAAAGGAAAGAGCTCAAGTTGAAAAGACCATTTCTGTTGTCATTGCT<br>ATCGCTGGTGTCTTAGTCGTTGCTTACGGTGACGGTGCTGACGACGGTTCCACCTCTAACGGTGAAAA<br>GGCTAGATTGGGTGGTAACGTCTTGATCGGTATCGGTTCTGTCTTGTATGGTTTATACGAAGTCTTGTA<br>TAAGAAGTTATTATGTCCACCATCTGGTGCTTCCCCAGGTAGATCTGTTGTTTTCTCTAATACCGTTTGT<br>GCTTGCATCGGTGCTTTCACTTTGTTATTCTTGTGGATCCCATTGCCATTGTTGCACTGGTCCGGTTGG<br>GAAATTTTTGAATTGCCAACCGGTAAGACTGCTAAGTTATTGGGTATTTCCATTGCCGCTAACGCCACC<br>TTCTCTGGTTCTTTCTTGATCTTAATTTCTTTGACTGGTCCAGTTTTGTCCTCTGTTGCCGCCTTGTTGAC<br>CATTTTCTTGGTTGCTATTACTGACAGAATTTTATTCGGTAGAGAATTGACTTCTGCTGCCATTTTGGGT<br>GGTTTGTTGATCATCGCTGCCTTCGCTTTGTTATCTTGGGCTACTTGGAAGGAAATGATTGAAGAGAAC<br>GAGAAGGATACTATCGATTCCATCTCTGACGTTGGTGACCACGATGAC
Fg	Sequence reported[10]
Gc	ATGGCCGAAGACTCCATCTTCCCAAACAACTCCACCTCTCCATTGACCAACCCAATTGTTGTTGAAACC<br>ATTAAGGGTACCGCTTACATTCCATTACACTACTTGGATGATTTGCAATACGAAAAGATGTTGTTGGCTT<br>CCTTGTTCTCCGTTAGAATTGCTACTTCCTTCGTTGTTATTATTTGGTACTTCGTCGCTGTCAACAAGGC<br>TAAGAGATCTAAGTTTTTGTACATTGTCAACCAAGTTTCTTTGTTGATCGTTTTTATCCAATCCATTTTGT<br>CTTTGATTTACGTCTTCTCCAACTTCTCCAAGATGTCTACCATTTTGACCGGTGATTACACCGGTATCAC<br>TAAGAGAGACATTAACGTCTCTTGTGTTGCCTCCGTTTTCCAATTCTTGTTCATCGCTTGTATCGAATTG<br>GCTTTGTTCATCCAAGCTACTGTCGTTTTCCAAAAATCTGTTAGATGGTTGAAGTTTTCCGTTTCTTTGAT<br>CCAAGGTTCCGTCGCTTTGACTACTACCGCCTTGTACATGGCCATTATTGTCCAATCCATCTACGCTAC<br>TTTGAACCCATACGCTGGTAACTTGATTAAAGGTCGTTTCGGTTACTTATTAGCTTCTTTGGGTAAGATT<br>TTCTTCTCTATTTCTGTTACTTCTTGTATGTGTATCTTCGTTGGTAAGTTGGTCTTTGCTATTCACCAAAG<br>AAGAACTTTGGGTATTAAGCAATTCGACGGTTTGCAAATTTTGGTCATTATGTCTACTCAATCCATGATC<br>ATCCCAACTATTATCGTCTTGATGTCTTTTTTGAGACGTAACGCTGGTTCTGTTTACACCATGGCTACCT<br>TGTTGGTCGCTTTGTCCTTGCCATTGTCCTCCTTGTGGGCTGAAGCCAAGACTACCAGAGACTCTGCTT<br>CTTACACCGCTTACAGACCATCTGGTTCTCCAAACAACCGTTCTTTGTTCGCCATCTTCTCTGATAGATT<br>GGCTTGTGGTTCTGGTAGAAACAACAGACACGATGATGATTCTAGAGGTAACGGTTCTGTTAACGCCA<br>GAAAGGCTGACGTCGAATCTACTATCGAAATGTCCTCTTGTTACACTGATTCCCCAACCTACTCCAAGT<br>TCGAAGCTGGTTTGGACGCTAGAGGTATCGTCTTCTACAACGAACACGGTTTGCCAGTTGTCTCCGGT<br>GAAGTTGGTGGTTCTTCCTCCAACGGTACTAAGTTGGGTTCTGGTCATAAGTACGAAGTCAACACTACT<br>GTTGTTTTGTCTGATGTTGACTCTCCATCTCCAACCGACGTCACCCGTAAG
Hj	ATGTCTTCCTTCGACCCATACACTCAAAACATTACTATTTTGGTTTCTCCATCCTCTCCACCAATTTCCAT<br>TCCAATCCCAGTTATCGACGCTTTCAACGACGAAACCGCTTCTATCATTACTAACTACGCCGCTCAATTA<br>GGTGCTGCTTTGGCCATGTTATTAGTTTTGTTGGCCGCTACTCCAACCGCTAGATTGTTAAGAGCTGAT<br>GGTCCATCCTTGTTGCACGCTTTGGCCTTGTTAGTCTGTGTCGTCAGAACTGTCTTATTGATCTACTTCT<br>TCTTGACCCCATTCTCTCACTTCTACCAAGTCTGGACCGGTGACTTCTCTCAAGTTCCAGCTTGGAACT<br>ACAGAGCTTCTATTGCTGGTACCGTTTTGTCTACTTTGTTGACCGTTGTTACCGACGCTGCTTTGGTTAA<br>CCAAGCTTGGACTATGGTTTCTTTATTCGCTCCAAGAACTAAGAGAGCCGTTTGTGTTTTGTCCTTGTTA<br>ATCACCTTGTTGGCCATTTCTTTCAGAGTCGCTTACACCGTCATTCAATGTGAAGGTATCGCTGAATTG<br>GCTGCTCCAAGACAATACGCTTGGTTGATCAGAGCCACTTTGATCTTTAACATCTGTTCCATTGCCTGG<br>TTCTGTGCTTTGTTCAACTCTAAGTTGGTTGCTCACTTGGTTACCAACAGAGGTGTCTTGCCATCCCGTA<br>GAGCCATGTCCCCAATGGAAGTTTTGATTATGGCCAACGGTATCTTGATGATTGTTCCAGTTGTTTTCG<br>CTATCTTGGAATGGCACCACTTCATTAACTTCGAAGCTGGTTCTTTAACCCCAACCTCCATCGCCATTAT<br>CTTGCCATTGTCCTCTTTGGCCGCCCAAAGAATCGCCAACACTTCTTCCTCT

Kl	ATGTCAGAAGAGATACCCAGTTTGAACCCATTGTTCTACAATGAGACATATAATCCATTGCAGTCCGTC<br>CTAACATACAGTTCAATTTACGGAGATGGGACTGAAATAACATTTCAACAGCTACAAAATCTTGTCCATG<br>AAAACATCACCCAAGCAATTATTTTTGGAACAAGGATCGGCGCTGCTGGATTAGCGTTGATTATAATGT<br>GGATGGTCTCTAAGAATAGAAAGACGCCGATATTCATAATAAATCAGAGTTCTTTGGTTCTTACAATTGT<br>TCAATCTGCTTTATATCTATCATATTTGTTGAGCAATTTTGGAGGAGTTCCCTTTGCTCTAACTTTGTTCC<br>CACAGATGATAGGCGACCGTGACAAACATCTTTACGGTGCCGTGACTCTAATTCAATGTCTATTGGTTG<br>CGTGTATTGAGGTCTCGTTAGTCTTTCAGGTAAGAGTCATTTTCAAAGCAGATAGATATAGGAAGATAG<br>GAATCATTTTGACTGGCGTCTCCGCTAGTTTTGGTGCTGCAACTGTAGCCATGTGGATGATTACTGCAA<br>TAAAATCTATTATTGTAGTGTATGATAGTCCATTGAACAAAGTTGACACATATTATTACAACATAGCAGTT<br>ATTTTACTTGCATGTTCAATAAATTTCATCACTCTTCTTCTATCAGTGAAACTTTTCCTGGCTTTCAGAGC<br>TAGGAGACATTTAGGTTTGAAACAATTTGACTCATTTCACATTCTACTCATCATGTCTACTCAGACATTAA<br>TAGGTCCATCGGTTTTGTATATTCTCGCCTACGCGCTGAACAATAAAGGAGTTAAGTCGTTGACTTCTAT<br>TGCTACATTGCTTGTAGTTCTTTCCCTACCTTTGACATCTATCTGGGCTGCTGCTGCAAATGATGCACCA<br>AGTGCCAGTACTTTCTATCGCCAATTCAACCCTTACTCTGCACAAAATCGTGATGATTCATCATCCTACT<br>CTTATGGTAAAGCCTTTAGTGACAAATACTCTTTCAGTAACTCACCACAAACTTCGGATGGTTGTAGTTC<br>AAAGGAACTTGAACTATCTACACAGTTGGAGATGGATTTAGAGTCTGGCGAATCTTTTATGGATAGAGC<br>AAAAAGGTCCGATTTTGTTTCTTCTCCAGGATCAACAGATGCAACAGTGATTAAACAATTGAAAGCTTCC<br>AACATCTATACCTCAGAAACAGATGCTGATGAAGAGGCAAGGGCATTTTGGGTGAATGCAATTCATGAA<br>AACAAAGATGACGGTTTAATGCAATCGAAAACCGTATTCAAAGAATTAAGA
Kp	ATGGAAGAATACTCCGACTCCTTCGACCCATCCCAACAATTGTTGAACTTCACTTCCTTATACGGTGAAA<br>CCGATGCTACTTTCGCTGAATTGGACGACTACCACTTCTACGTCGTTAAGTACGCCATCGTTTACGGTG<br>CCAGAATTGGTGTCGGTATGTTTTGTACTTTGATGTTGTTCGTTGTTTCCAAGTCTTGGAAGACTCCAAT<br>CTTCGTCTTGAACCAATCTTCTTTGATTTTGTTGATTATTCACTCCGGTTTCTACATCCACTACTTGACCA<br>ACCAATTCTCTTCCTTGACCTACATGTTCACTAGAATCCCAAACGAAACCCATGCTGGTGTCGATTTGC<br>GTATTAACGTCGTTACCAACACCTTGTACGCTTTGTTGATCTTATCTATTGAAATTTCCTTAATTTACCAA<br>GTCTTCGTTATCTTCAAAGGTGTCTACGAAAACTCTTTAAGATGGATTGTTACTATTTTCACCGCTTTATT<br>CGCCGCCGCCGTCGTTGCTATTAACTTCTACGTCACTACTTTGCAATCTGTCTCTATGTACAACTCTAAC<br>GTTGACTTTCCAAGATGGGCTTCTAACGTCCCATTGATCTTGTTCGCTTCTTCTGTCAACTGGGCTTGTT<br>TGTTGTTGTCCTTGAAGTTGTTCTTCGCTATCAAGGTTAGAAGATCTTTGGGTTTGAGACAATTCGACAC<br>TTTTCACATCTTGGCCATCATGTTCTCTCAAACTTTGATTATCCCATCCATTTTGATTGTCTTGGGTTACA<br>CTGGTACCAGAGACAGAGACTCCTTGGCTTCTTTGGGTTTCTTGTTGATCGTTGTTTCTTTGCCATTTTC<br>CTCTATGTGGGCTGCCACTGCTAACAACTCCAACATCCCAACCTCTACCGGTTCTTTCGCCTGGAAGAA<br>CAGATACTCCCCATCTACTTACTCCGACGATACCACTGCTGTTTCCAAGTCCTTCACTATTATGACCGCT<br>AAGGATGAATGTTTCACCACTGATACCGAAGGTTCTCCAAGATTCATCAAGGGTGACAGAACCTCCGAA<br>GATTTGCACTTC
Le	Sequence reported[10]
Mg	ATGGTGGTAACAGCTCCACCTTCAGTTGACAGAACATATTTTATCCCGAATTCTACCTTTGATCCATATC<br>AACAAGACTTGACGTTGGTCTATCCCGATGGTGTGCACGCCCTGGTTGCTAACGTTGATGATATAGTGT<br>ACTTCATGGGTCTAGCAGTTAAGTCTACGCTAATATTTGCTATTCAAATTGGTATTTCATTTGTATTAATG<br>TTGGTTATTGCCCTGTTGACGAAACCTGAAAGAAGAGTTACGTTGGTATTCTTCTTAAACATGACTGCAC<br>TTTTTACCATCTTCATCAGAGCCATATTGATGTGTACTACATTTGTTGGTACATATTACAATTTTTACAAC<br>TGGATTATGGGCAACTACCCGAACTCTGGTTTAGCTGATCGTGTATCTATTGCAGCCGAAGTTTTTGCT<br>TTTCTGATTATACTGTCATTAGAACTTTCTATGATGTTTCAAGTTCGTATTGTATGCATCAACCTGAGCTC<br>ATTCAGGAGGAGAATAATTACTTTTAGTAGTATAGTGGTTGCAATGATTGTTTGTACAGTTAGATTTGCC<br>CTTATGGTGTTGTCTTGTGATTGGAGGATTGTGAATATCGGAGATGCGACGCAAGAAAAGAACAGAATC<br>ATTAACCGTGTGGCATCCGGTTATAACATATGCACAATAGCATCAATCATTTTTTTCAACACCATCTTCG<br>TCTCCAAGTTGGCCGTCGCTATCAAACATCGTAGAAGCATGGGCATGAAACAATTCGGTCCAATGCAG<br>ATCATCTTTGTTATGGGTTGTCAAACGCTTCTAATTCCAGCCATCTTTGGAATTATATCTTACTTTGCTCT<br>AGCTAGCACTCAGGTCTACTCTTTAATGCCAATGGTCGTAGCTATCTTCTTACCATTAAGTTCTATGTGG<br>GCTAGTTTTAACACCAACAAAACCAACAGTGTTACAAATATGAGGCAACCAAACGTCTATAGGCCTAATA<br>TGATCATCGGTCAAGACACAACCCAAAATTCCGGAAAGAATACAAACATAAGTGGTACGTCAAACTCCA<br>CGGCAACTACAAGTAGTTTTGCTAGCGATAAGAGACGTCTAAATTTATCTTTCAATACACAAGGTACACT<br>GGTTAATTCAATAAGTGAAGAAGAGGTTAATAACCCACAAAAATTGGGTCCTTCCGCTACCGTTGCGGT<br>AATGGATAGAGATTCTTTGGAATTAGAGATGAGACAACACGGCATCGCTCAAGGTAGGTCATACTCAGT<br>CCGTTCCGAC
Mo	Sequence reported[10]

Nc	ATGGCGTCCTCTTCCTCACCACCTGCAGACATTTTCTCAGGGATCACGCAATCACTAAATAGTACACAC<br>GCGACGCTTACACTACCGATTCCGCCAGCGGACAGGGATCATCTGGAAAATCAAGTATTATTTTTGTTT<br>GACAATCACGGTCAGTTACTTAATGTAACTACAACTTACATTGACGCTTTTAACAATATGCTGGTCTCTA<br>CTACTATAAACTATGCAACGCAAATTGGAGCTACTTTTATAATGCTAGCCATTATGTTATTAATGACTCCC<br>AGAAGGAGGTTCAAACGTTTACCAACAATTATTAGCTTGTTAGCCTTATGTATTAATTTGATCAGGGTGG<br>TTTTGCTGGCCCTGTTTTTTCCTTCTCACTGGACAGACTTCTACGTGTTGTATTCCGGTGACTGGCAGTT<br>TGTACCTCCAGGGGATATGCAAATATCTGTTGCTGCTACGGTTTTGTCTATCCCAGTGACGGCATTATT<br>ATTGAGCGCATTGATGGTTCAAGCCTGGTCAATGATGCAATTATGGACACCACTGTGGAGGGCACTAG<br>TGGTACTAGTGTCCGGGCTATTGTCACTGGTAACTGTGGCAATGAGTTTCGCGAATTGCATTTTCCAAG<br>CGAAAAATATTTTGTATGCCGACCCTTTACCCTCCTACTGGGTCAGAAAATTGTACTTAGCATTAACGAC<br>TGGGTCTATAAGTTGGTTCACATTCCTTTTTATGATAAGATTGGTTATGCATATGTGGACAAACAGATCT<br>ATATTACCAAGCATGAAGGGTTTGAAGGCTATGGATGTATTGATTATTACGAATTCTATATTGATGTTAAT<br>CCCAGTGTTGTTTGCAGGCTTGGAATTTCTGGATAGTGCCTCTGGATTTGAGTCCGGGTCTTTGACTCA<br>AACCTCTGTAGTGATTGTCCTGCCTTTGGGTACTTTAGTAGCACAAAGAATAGCTACGAGGGGTTACAT<br>GCCCGATAGTCTGGAGGCTTCTAGCGGACCAAATGGTTCATTGCCGTTATCTAATTTAAGTTTCGCTGG<br>AGGGGGCGGTGGTGGTTCTGGGGGACATAAAGATAAAGAAAACGGTGGCGGTATTATACCGCCTACT<br>ACGAACAATACTGCTGCTACTAATTTTTCTTCATCAATCGCGTGTTCTGGTATATCTTGTTTACCAAAAGT<br>CAAAAGAATGACCGCGAGTTCAGCCTCAAGTAGCCAGAGACCGTTGTTGACAATGACTAACTCAACCAT<br>AGCGAGTAATGACAGTTCAGGTTTCCCTTCTCCTGGCATACATAATACCACTACTACGACAACACAATA<br>CCAATATTCCATGGGAATGAACATGCCGAACTTTCCTCCAGTCCCGTTCCCAGGTTACCAGTCACGTAC<br>TACCGGTGTTACTTCCCATATTGTGTCCGACGGTAGACATCACCAGGGTATGAACAGGCACCCATCTGT<br>TGACCATTTTGATAGGGAACTTGCTAGGATTGATGATGAAGATGACGATGGTTACCCTTTCGCATCAAG<br>TGAAAAGGCCGTTATGCACGGAGACGATGACGACGATGTGGAAAGGGGACGTCGTAGAGCTCTACCA<br>CCATCCTTAGGTGGAGTTAGAGTTGAAAGGACGATCGAGACCAGGAGCGAGGAACGTATGCCATCTCC<br>GGACCCATTGGGTGTTACGAAGCCTAGATCATTCGAG
Pb	Sequence reported[10]
Pd	ATGTCCACTGCCAACGTTCATTTACCAGCTGATTTCGATCCAACTAGACAAAACATCACTATCTATACCC<br>CAGACGGTACCCCAGTTGTTGCTACCTTGCCAATGATCAATTTGTTTAACAGACAAAACAACGAAATCT<br>GTGTTGTTTACGGTTGTCAATTGGGTGCCTCTTTAATTATGTTCTTGGTTGTTTTGTTGACCACCAGAGT<br>TTCCAAGAGAAAATCTCCAATCTTCGTCTTGAACGTTTTGTCTTTGATTATTTCTTGTTTAAGATCCTTGT<br>TGCAAATTTTATACTATATTGGTCCATGGACCGAGATCTACAGATACTTGTCTTTCGATTACTCTACTGT<br>CCCAGCTTCCGCTTACGCTAATTCTGTTGCTGCCACTTTATTAACCTTATTCTTATTGATTACCATTGAAG<br>CTTCTTTAGTTTTACAAACTAACGTTGTCTGCAAGTCTATGTCTTCTCACATTCGTTGGCCAGTTACTGC<br>TTTGTCCATGGTTGTCTCTTTATTGGCTATTTCTTTTAGATTCGGTTTGACCATCCGTAACATCGAAGGT<br>ATCTTAGGTGCTACTGTCAAATCCGACTCCTTAATGTTCTCTGGTGCCTCTTTGATCTCTGAAACTGCTT<br>CTATCTGGTTCTTCTGCACTATTTTCGTTATTAAATTGGGTTGGACCTTGTACCAAAGAAAGAAGATGGG<br>TTTGAAGCAATGGGGTCCAATGCAAATTATCACTATCATGGCTGGTTGCACCATGTTGATCCCATCCTT<br>GTTCACTGTTTTGGAATTCTTCCCTGAAGAAACTTTCTACGAGGCCGGTACTTTGGCTATCTGTTTGGTT<br>GCTATTTTGTTGCCATTATCTTCCGTCTGGGCTGCCGCTGCTATTGATGGTGATGAACCAGTCCGTCCA<br>CATGGTTCTACCCCAAAATTCGCTTCTTTCAACATGGGTTCCGACTACAAATCTTCTTCTGCTCACTTGC<br>CAAGATCTATTAGAAAGGCCTCCGTCCCAGCTGAACATTTATCTAGAACTTCTGAAGAAGAGTTAGGTG<br>ACGACGGTACTTTGAACAGAGGTGGTGCCTACGGTATGGACAGAATGTCCGGTTCTATCTCCCCTAGA<br>GGTGTCAGAATTGAAAGAACTTACGAAGTTCATACCGCTGGTAGAGGTGGTTCTATCGAGAGAGAGGA<br>CATCTTC
Pr	ATGGCTACCTCTTCCCCAATCCAACCATTTGACCCATTCACCCAAAACGTTACCTTCCGTTTGCAAGAC<br>GGTACCGAATTCCCAGTTTCTGTCAAGGCTTTGGACGTCTTCGTCATGTACAACGTTAGAGTCTGTATT<br>AACTACGGTTGTCAATTCGGTGCCTCCTTCGTCTTGTTAGTCATTTTAGTCTTGTTAACTCAATCCGACA<br>AGAGAAGATCTGCTGTCTTCATTTTGAACGGTTTGGCTTTGTTCTTGAACTCTTCTAGATTGTTGTTTCA<br>AGTTATTCACTTCTCCACTGCCTTCGAACAAGTCTACCCATACGTCTCTGGTGACTACTCCTCTGTCCCA<br>TGGTCCGCTTACGCTATCTCCATTGTCGCTGTTGTTTTGACTACCTTGGTCGTTGTTTGTATCGAAGCTT<br>CTTTGGTTATTCAAGTTCACGTTGTCTGTTCCACCTTGAGACGTAGATACAGACACCCATTATTAGCTAT<br>TTCTATTTTGGTCGCTTTGGTTCCAATCGGTTTCAGATGTGCTTGGATGGTCGCTAACTGTAAGGCTATT<br>ATTAAATTGACCTACACCAACGACGTTTGGTGGATCGAATCTGCTACTAACATCTGTGTCACTATCTCCA<br>TCTGTTTCTTCTGTGTTATCTTCGTTACCAAGTTGGGTTTCGCCATCAAGCAAAGAAGAAGATTGGGTGT<br>TAGAGAATTCGGTCCAATGAAGGTTATTTTCGTCATGGGTTGTCAAACTATGGTTGTTCCAGCTATTTTC<br>TCCATCACCCAATACTACGTCGTCGTCCCAGAATTCTCCTCTAACGTCGTTACTTTGGTTGTCATTTCTT<br>TACCATTATCTTCCATTTGGGCCGGTGCTGTCTTGGAAAACGCTAGAAGAACCGGTTCCCAAGATAGAC<br>AAAGAAGACGTAACTTGTGGAGAGCTTTGGTTGGTGGTGCTGAATCCTTGTTATCCCCAACTAAGGACT<br>CTCCAACCTCTTTGTCTGCTATGACTGCTGCTCAAACCTTATGTTACTCTGATCACACCATGTCCAAGG<br>GTTCTCCAACTTCCAGAGACACCGATGCTTTCTACGGTATCTCCGTTGAACACGACATCTCCATTAACA<br>GAGTTCAACGTAACAACTCCATCGTC

Sc	Sequence reported[10]
Scas1	ATGTCTGACGCTCCACCACCATTGTCCGAATTGTTCTACAACTCCTCCTACAACCCAGGTTTGTCTATCA<br>TTTCTTACACTTCCATTTACGGTAACGGTACTGAAGTTACCTTTAACGAATTACAATCTATCGTCAACAA<br>GAAGATTACTGAAGCTATCATGTTCGGTGTCAGATGTGGTGCCGCTATTTTGACTATCATTGTCATGTG<br>GATGATTTCTAAGAAGAAAAAGACCCCAATTTTCATCATCAACCAAGTTTCTTTATTCTTGATTTTGTTGC<br>ACTCCGCTTTCAACTTCAGATACTTGTTGTCTAACTACTCTTCCGTCACTTTCGCCTTGACCGGTTTCCC<br>ACAATTCATCCACAGAAACGACGTCCACGTCTACGCTGCTGCTTCTATCTTCCAAGTCTTGTTGGTCGC<br>TTCTATTGAAATTTCCTTAATGTTCCAAATCAGAGTCATTTTCAAGGGTGATAACTTCAAGAGAATTGGTA<br>CTATCTTGACCGCTTTGTCCTCTTCTTTGGGTTTAGCTACTGTTGCTATGTACTTTGTCACCGCTATTAA<br>GGGTATTATTGCTACCTACAAGGATGTTAACGATACTCAACAAAAGTACTTCAACGTTGCTACTATCTTG<br>TTGGCTTCCTCTATCAACTTTATGACCTTGATCTTGGTTATCAAGTTGATCTTGGCTATCAGATCCAGAA<br>GATTCTTGGGTTTGAAACAATTCGACTCTTTCCATATCTTGTTGATCATGTCTTTTCAATCTTTGTTGGCC<br>CCATCCATTTTGTTCATTTTGGCTTACTCTTTGGACCCAAACCAAGGTACCGACGTCTTGGTTACTGTCG<br>CTACTTTGTTGGTCGTCTTATCTTTGCCATTGTCCTCCATGTGGGCTACTGCTGCTAACAACGCCTCCA<br>GACCATCCTCTGTTGGTTCCGACTGGACTCCATCTAACTCCGACTACTACTCTAACGGTCCATCTTCTG<br>TCAAGACCGAATCTGTCAAATCTGATGAAAAGGTCTCCTTGAGATCCAGAATTTACAACTTGTACCCAAA<br>GTCTAAGTCTGAATTCGAACAATCCTCCGAACACACTTACGTTGACAAGGTCGACTTGGAAAACAACTT<br>CTACGAATTGTCCACCCCAATCACCGAAAGATCTCCATCTTCTATCATTAAGAAGGGTAAGCAAGGTAT<br>TTCTACTAGAGAAACCGTCAAAAAGTTGGACTCCTTGGATGACATTTACACTCCAAACACTGCTGCTGA<br>TGAAGAAGCCAGAAAGTTCTGGTCTGAAGATGTTTCTAACGAATTGGATTCCTTACAAAAAATCGAAACT<br>GAAACTTCCGATGAATTATCCCCAGAAATGTTACAATTGATGATTGGTCAAGAAGAAGAAGACGATAAC<br>TTATTGGCTACCAAGAAGATCACCGTCAAGAAGCAA
She	ATGAAACCCGCCGCTGGACCTGCATCTAGTCCATTCGACCCATTTAACCAAACGTTTTACCTGACCGGT<br>CCAGATAATACCACTGTACCAGTCTCAGTCCCACAAGTTGACTATATCTGGCATTATATTATTGGAACAT<br>CCATCAACTATGGTTCTCAGATCGGAGCCTGTTTACTTATGCTTCTTGTGATGTTGACATTGACTTCAAA<br>GTCAAGATTTTCTCGTGCGGCCACTCTGATTAACGTAGCAAGCTTATTGATTGGAGTAATTCGTTGTGTT<br>CTTTTAGCTGTCTACTTTACTTCTTCTCTAACTGAATTGTATGCTCTGTTCGTTGGCGATTACAGCCAGG<br>TCCGTAGGTCTGATCTTTGTGTCTCTGCTGTGGCAACCTTCTTTAGTCTACCACAATTAGTTCTAATAGA<br>AGCTGCTTTGTTTCTACAGGCTTATAGTATGATCAAAATGTGGCCATCCCTGTGGAGAGCAGTGGTTTT<br>AGCTATGTCAGTGGTGGTGGCTGTGTGTGCAATCGGTTTTAAGTTCGCGTCCGTTGTTATGCGTATGAG<br>GTCAACATTAACATTGGACGATTCTTTGGATTTCTGGCTAGTGGAAGTCGATCTGGCTTTTACAGCAACT<br>ACTATTTTTTGGTTTTGTTTCATCTACATTATAAGGTTGGTTATTCATATGTGGGAATATAGAAGCATTTT<br>ACCACCAATGGGGTCTGTTTCTGCTATGGAGGTTCTTGTTATGACCAATGGAGCGTTGATGTTAGTTCC<br>AGTGATTTTCGCCGCAATAGAAATCAATGGTTTATCAAGCTTTGAATCAGGGTCACTGGTTCATACATCA<br>GTGATTGTATTATTACCTTTAGGTAGCTTGATAGCGCAAGCAATGACACGTCCAGATGGGTATGTCCAA<br>AGAACGAATACATCTGGAGCATCAGGCGCAAGTGGTGCACATCCTGGTAGAAATGGATCCGGACACG<br>GTGGTCATGGTGGTGCGTACTCAAGAGCCATGACTAATACCCTAAATACATTGGATACATTGGATACCG<br>TAGACAGTAAGACATCCATAATGCATCATCATCATCACCATCATAGAAACCACTCAAATGGCATGAGTAA<br>GACGAAGGCAAATAGTGGAACATGGAGCCATGCGTCAGATGCTAACTCCACCAATGCTATGATCAGCG<br>GTGGTATCGCAACTCAAGTTAGGATTCAAGCTAATCAGTCAACCTTAGGAAATACGGGGATGTCCGGG<br>GGCTCTGGAGCCCCTAATTCTCATACTCGTAATAACTCATTGGCTGCTATGGAACCAGTGGAGAAGCAA<br>CTGCATGATATCGATGCCACACCTTTAAGCGCATCTGATTGCAGGGTCTGGGTTGATCGTGAGGTCGA<br>GGTCAGAAGGGACATGGTC
Sj	ATGTACTCCTGGGACGAATTCAGATCCCCAAAGCAAGCTGAAGTTTTGAACCAAACCGTTACCTTGGAA<br>ACTATTGTTTCCACCATTCAATTGCCAATCTCTGAAATTGACTCCATGGAAAGAAACAGATTGTTGACCG<br>GTATGACTGTCGCTGTTCAAGTTGGTTTAGGTTCCTTCATTTTAGTTTTGATGTGTATTTTCTCTTCCTCT<br>GAAAAGAGAAAGAAGCCAGTCTTCATCTTCAACTTCGCTGGTAACTTGGTTATGACTTTGAGAGCTATTT<br>TCGAAGTTATCGTTTTGGCTTCTAACAACTACTCTATCGCTGTTCAATACGGTTTCGCTTTTGCTGCCGT<br>CAGACAATACGTTCACGCCTTCAACATTATCATCTTGTTGTTGGGTCCATTCATCTTGTTCATCGCTGAA<br>ATGTCTTTGATGTTGCAAGTTAGAATCATTTGTTCCCAACACAGACCAACTATGATTACCACCACTGTTA<br>TCTCTTGTATTTTCACTGTTGTTACCTTGGCCTTCTGGATCACCGACATGTCTCAAGAAATTGCTTACCA<br>ATTGTTCTTGAAAAACTACAACATGAAGCAAATTGTTGGTTACTCCTGGTTGTACTTTATCGCTAAGATC<br>ACCTTCGCTGCTTCCATTATCTTCCATTCCTCCGTCTTCTCCTTCAAATTGATGCGTGCTATTTACATTC<br>GTAGAAAGATCGGTCAATTCCCATTCGGTCCAATGCAATGTATCTTCATTGTTTCCTGTCAATGTTTGAT<br>CGTTCCAGCTATTTTCACTTTGATCGATTCTTTCACCCACACTTACGATGGTTTCTCCTCCATGACTCAA<br>TGTTTGTTGATCATCTCCTTACCATTGTCTTCCTTGTGGGCCACCCACACCGCTCAAAAGTTGCAAACC<br>ATGAAGGATAACACTAACCCACCATCTGGTACCCAATTAACCATCAGAGTTGATCGTACTTTCGACATG<br>AAGTTCGTTTCCGACTCCTCTGACGGTTCTTTCACTGAAAAGACCGAAGAAACTTTGCCA

Sk	ATGTCCGGTAAGCAAGACTTGTCTCCATTAGGTTTGTACTCTTCTTACGACCCTACCAAGGGTTTGATTT<br>CTTACACCTCCTTGTACGGTTCTGGTACTACTGTTACTTTCGAAGAATTGCAAATCTTTGTTAACAAGAA<br>AATTACCCAAGGTATTTTGTTCGGTACTAGAATCGGTGCCGCCGGTTTAGCTATCATCGTCTTATGGAT<br>GGTCTCTAAGAACAGAAAGACTCCAATTTTCATTATTAACCAAATCTCCTTGTTCTTGATCTTGTTGCACT<br>CCTCTTTGTTCTTGAGATACTTGTTGGGTGATTACGCTTCTGTCGTCTTCAACTTTACCTTATTCTCCCAA<br>TCCATCTCCAGAAACGATGTCCACGTCTACGGTGCCACCAACATGATTCAAGTCTTGTTGGTTGCCGCT<br>GTTGAAATTTCTTTGATTTTTCAAGTCAGAGTTATTTTCAAAGGTGATTCTTACAAAGGTGTCGGTAGAAT<br>CTTGACCTCTATCTCTGCCGTCTTGGGTTTCACTACCGTCGTCATGTACTTCATTACTGCCGTTAAGTCC<br>ATGACCTCCGTTTACTCTGATTTGACTAAGACTTCCGACCGTTACTTCTTTAATATCGCTTCTATTTTATT<br>GTCTTCTTCCGTTAACTTTATGACCTTGTTATTGACCGTCAAGTTAATTTTGGCCGTCAGATCTCGTAGA<br>TTCTTGGGTTTGAAGCAATTCGATTCCTTCCATGTTTTGTTGATTATGTCCTTCCAAACTTTGATCTTCCC<br>ATCTATCTTATTCATCTTGGCTTACGCCTTAAACCCAAACCAAGGTACCGACACTTTAACTTCCATTGCT<br>ACCTTGTTAGTCACTTTGTCTTTGCCTTTGTCTTCTATGTGGGCTACCTCTGCTAACAACTCCTCCCACC<br>CATCCTCTATCAACACCCAATTCCGTCAAAGAAACTATGACGACGTCTCCTTCAAGACCGGTATTACCT<br>CTTTCTACTCCGAATCTTCTAAGCCTTCTTCCAAGTACAGACATACTAACAACTTATATGACTTATACCCA<br>GTCTCCCGTACCTCTAACTCCAGATGTAACGGTTACCCAAACGACGGTTCTAAATTAGCTCCAAATCCA<br>AACTGTGTTGGTCACAACGGTTCTACTATGTCCGTTAACGACAAGAACGGTGCTCATGCTACCTGTGTT<br>CAAAATAACGTCACCTTGAACACCGACTCCACTTTGAACTACTCTAACGTTGACACCCAAGACACTTCC<br>AAGATCTTGATGACCACC
Sn	ATGGCTTCTATGGTTCCACCACCAGATTTTGACCCTTACACCCAAGAGTTCATGGTTTTAGGTCCAGAT<br>GGTCAAGAAATCCCAATCTCCATGCAAACCGTCAACGAATACCGTTTGTACACCGCTCGTTTGGGTTTG<br>GCTTATGGTTCCCAAATTGGTGCCACCTTATTGTTATTGTTGGTTTTGTCTTTGTTAACTAGAAGAGAAA<br>AGAGAAAGTCCGGTATTTTTATTGTTAACGCTTTGTGTTTGGTTACTAACACCATCAGATGTATTTTGTTG<br>TCCTGCTTTGTCACTTCCACCTTGTGGCACCCATACACCCAATTCTCTCAAGATACTTCCAGAGTTTCCA<br>AAACTGACGTTAACACCTCTATCGCTGCCTCTATTTTCACTTTGATTGTCACTGTTTTAATCATGATCTCC<br>TTATCTGTTCAAGTTTGGGTTGTTTGTATTACCACTGCTCCATACCAAAGATACATGATTATGGGTGCTA<br>CCACCGCTACTGCCATGGTCGCCGTTGGTTACAAGGCTGCTTTTGTTATCACTTCCATCATTCAAACTTT<br>AAACGGTCAAGACGGTGGTTCCTACTTGGATTTGGTCATGCAATCTTACATCACTCAAGCTGTCGCTAT<br>TTCTTTCTATTCCTGTATTTTCACTTACAAGTTAGGTCACGCTATTGTTCAAAGAAGAACCTTGAATATGC<br>CACAATTTGGTCCAATGCAAATTATCTTCATCATGGGTTCTTTATTCACTGGTTTACAATTCGTCAAGAAC<br>GTCGATGAATTGGGTATTATCACCCCTACCATTGTTTGTATCTTTTTGCCATTGTCCGCTATCTGGGCTG<br>GTGTCGTCAACGAAAAGGTTGTCGGTGCTAATGGTCCAGACGCTCATCACAGATTGTTGCAAGGTGAA<br>TTCTACAGAGCTGCTTCTAACTCCACTTACGGTTCTAACTCTTCCGGTACTGTTGTCGACAGATCCAGA<br>CAAATGTCTGTCTGTACTTGTGCTTCTTCTTCCCCATTTGTTAGAAAGAAGTCTGTTGCCGAATGGGAC<br>GATGAAGCTATTTTAGTTGGTAGAGAATTCGGTTTCTCCCGTGGTGAAGTCGGTGAAAGAGGT
So	ATGCGTGAACCATGGTGGAAGAACTACTACACCATGAACGGTACCCAAGTCCAAAACCAATCCATCCC<br>AATTTTGTCCACCCAAGGTTACATTCAAGTTCCATTGTCCACCATCGATAAGGCTGAAAGAAACAGAATT<br>TTGACTGGTATGACCGTTTCTGCTCAATTGGCCTTGGGTGTCTTGATCATGGTCATGTCTATTTTGTTGT<br>CCTCCCCAGAAAAGAGAAAGACCCCAGTTTTCATCGTCAACTCTGCCTCTATCATTTCCATGTGTATTAG<br>AGCTATCTTGATGATTGTCAACTTGTGTTCTGAATCCTACTCTTTGGCTGTTATGTACGGTTTCGTCTTC<br>GAATTGGTTGGTCAATACGTTCACGTTTTTGACATTTTGGTTATGATTATTGGTACCATCATCATTATTAC<br>CGCTGAAGTTTCCATGTTGTTGCAAGTCAGAATTATTTGTGCTCACGACAGAAAGACTCAAAGAATTGTT<br>ACCTGTATCTCTTCTGGTTTATCCTTGATCGTCGTTGCCTTCTGGTTCACTGATATGTGTCAAGAAATTA<br>AGTACTTGTTGTGGTTGACCCCATACAACAACCACCAAATCTCTGGTTACTACTGGGTTTACTTCGTCG<br>GTAAGATCTTGTTCGCCGTTTCCATTATGTTCCACTCTGCCGTCTTCTCCTACAAGTTGTTCCACGCTAT<br>CCAAATTAGAAAGAAGATTGGTCAATTCCCATTCGGTCCAATGCAATGTATTTTAATTATTTCCTGTCAAT<br>GTTTGTTCGTTCCAGCTATTTTCACTATCATCGACTCTTTCATCCACACTTACGACGGTTTTTCCTCCAT<br>GACCCAATGTTTGTTGATCGTCTCTTTGCCATTGTCCTCCTTGTGGGCCTCTTCCACTGCTTTAAAGTTG<br>CAATCTTTGAAGTCTACCACCTCTCCAGGTGACACTACTCAAGTTTCCATTAGAGTCGACAGAACCTAC<br>GACATCAAGAGAATCCCAACTGAAGAATTGTCTTCTGTTGACGAAACCGAAATCAAGAAGTGGCCA

Sp	ATGAGACAACCATGGTGGAAAGACTTTACTATTCCCGATGCATCCGCAATTATTCACCAAAATATTACCA<br>TTGTCTCTATTGTAGGAGAGATTGAAGTGCCAGTTTCAACAATTGATGCATATGAAAGAGATAGACTTTT<br>AACTGGAATGACTTTGTCTGCCCAACTTGCTTTAGGAGTCCTTACCATTTTGATGGTTTGTCTATTGTCA<br>TCATCCGAAAAACGAAAACACCCAGTTTTTGTTTTTAATTCGGCAAGTATTGTTGCAATGTGTCTTCGGG<br>CCATTTTGAATATAGTGACCATATGCAGCAATAGCTACAGTATCCTGGTTAATTACGGGTTTATCTTAAA<br>CATGGTTCATATGTATGTCCATGTGTTTAATATTTTAATTTTGTTGCTTGCACCGGTCATCATTTTTACTG<br>CTGAGATGAGCATGATGATTCAAGTTCGTATAATTTGTGCACATGATAGAAAGACACAAAGGATAATGA<br>CTGTTATTAGTGCCTGCTTAACTGTTTTGGTTCTCGCATTTTGGATTACTAACATGTGTCAACAGATTCA<br>GTATCTGTTATGGTTAACTCCACTTAGCAGCAAGACCATTGTTGGATACTCTTGGCCCTACTTTATTGCT<br>AAAATACTTTTTGCTTTTAGCATTATTTTTCACAGTGGTGTTTTTTCATACAAACTCTTTCGTGCCATATTA<br>ATACGGAAAAAAATTGGGCAATTTCCATTTGGTCCGATGCAGTGTATTTTAGTTATTAGCTGCCAATGTC<br>TTATTGTTCCAGCTACCTTTACTATAATAGATAGTTTTATCCATACGTATGATGGCTTTAGCTCTATGACT<br>CAATGTCTGCTAATCATTTCTCTTCCTCTTTCGAGTTTATGGGCGTCTAGTACAGCTCTGAAATTGCAAA<br>GCATGAAAACTTCATCTGCGCAAGGAGAAACCACCGAGGTTTCGATTAGAGTTGATAGAACGTTTGATA<br>TCAAACATACTCCCAGTGACGATTATTCGATTTCTGATGAATCTGAAACTAAAAAGTGGACG
Ss	ATGGATACTAGTATCAATACTCTCAACCCTGCGAATATCATTGTCAACTACACCTTGCCAAATGATCCTA<br>GAGTAATTAGTGTCCCATTTGGAGCTTTTGACGAATATGTTAACCAATCTATGCAAAAGGCCATTATCCA<br>TGGAGTTTCCATTGGTTCATGCACCATAATGCTTTTAATTATTTTGATCTTCAATGTCAAACGCAAGAAGT<br>CGCCAGCTTTCTATCTTAATTCGGTTACGTTGACTGCAATGATTATTCGGTCTGCTCTTAATTTGGCATA<br>TTTGCTAGGTCCTTTGGCTGGATTAAGTTTTACGTTCTCCGGCTTGGTAACTCCAGAAACCAATTTCTCT<br>GTCTCTGAAGCCACCAATGCTTTCCAGGTTATTGTTGTTGCTCTTATCGAGGCGTCCATGACATTTCAG<br>GTGTTCGTCGTCTTCCAATCACCAGAAGTGAAGAAGTTGGGTATAGCTCTTACCTCCATATCTGCATTC<br>ACGGGTGCTGCTGCTGTAGGATTTACTATCAATAGTACAATCCAACAATCGAGAATTTATCATTCAGTTG<br>TCAATGGAACTCCTACGCCAACGGTCGCTACCTGGTCTTGGGTTAGAGATGTGCCTACGATACTTTTTT<br>CTACTTCGGTTAACATAATGTCTTTCATCTTGATTCTCAAGTTAGGGTTTGCCATAAAGACAAGAAGATA<br>CCTTGGCCTTCGGCAATTTGGCAGTTTGCACATCTTATTGATGATGGCTACTCAAACATTATTGGCCCC<br>ATCTATTCTCATTCTTGTACATTACGGATATGGCACATCTCTGAATAGCCAGCTCATTCTTATAAGTTACT<br>TGCTTGTTGTTTTGTCTTTACCAGTATCCTCTATCTGGGCAGCAACAGCCAACAATTCTCCTCAACTTCC<br>ATCTTCCGCAACTCTTTCATTCATGAACAAAACGACCTCTCACTTTTCTGAAAGC
Td	ATGTCTGACTCCGCCCAAAACTTGTCCGATTTGGCCTTCAACTCTTCTTATAACCCATTGGACTCCTTTA<br>TTACCTTTACCTCTATCTACGGTGATAACACTGCTGTTAAGTTCTCCGTTTTACAAGACATGGTTGACGT<br>TAATACTAATGAAGCCATCGTTTACGGTACCCGTTGTGGTGCTTCTGTCTTGACCCAAATTATCATGTGG<br>ATGATTTCTAAAAACAGAAGAACCCCAGTCTTTATTATTAACCAAGTTTCTTTGACTTTGATTTTAATTCA<br>CTCTGCCTTGTACTTCAAGTACTTGTTGTCTGGTTTCGGTTCCGTTGTCTACGGTTTGACTGCTTTCCCA<br>CAATTGATTAAGCCAGGTGATTTGAGAGCTTTCGCTGCTGCTAACATCGTTATGGTCTTGTTGGTCGCT<br>TCTATTGAAGCTTCCTTAATCTTCCAAGTCAAAGTTATCTTCACCGGTGATAACATGAAGAGAGTCGGTT<br>TAATCTTGACTATTATTTGTACTTGTATGGGTTTAGCTACTGTTACCATGTACTTTATTACTGCCGTCAAG<br>TCTATTGTCTCTTTGTACCGTGACATGTCTGGTTCCTCCACCGTTTTATATAACGTTTCTTTAATTATGTT<br>GGCTTCCTCCATCCACTTTATGGCTTTGATCTTGGTTGTCAAATTGTTCTTGGCTGTTAGATCTAGAAGA<br>TTCTTGGGTTTGAAACAATTCGATTCTTTCCACATTTTGTTGATCATCTCTTGTCAAACTTTGTTGGTTCC<br>ATCTTTATTATTCATTATTGCTTACTCTTTTCCATCTTCTAAGAACATTGAATCTTTGAAGGCTATCGCTGT<br>TTTGACCGTCGTTTTGTCTTTGCCATTGTCTTCTATGTGGGCTACTGCTGCTAATAACTTCACTAACTCT<br>TCCTCCTCCGGTTCCGACTCCGCTCCAACCAATGGTGGTTTCTACGGTAGAGGTTCTTCCAACTTGTAT<br>CCTGAAAAGACTGATAACAGATCCCCAAAGGGTGCCAGAAACGCTTTATACGAATTAAGATCTAAGAAC<br>AATGCTGAGGGTCAAGCTGATATTTACACCGTTACCGATATTGAAAACGATATTTTCAACGATTTGTCCA<br>AGCCAGTTGAGCAAAACATTTTCTCTGATGTTCAAATTATTGATTCTCATTCTTTGCATAAGGCTTGTTCT<br>AAAGAAGACCCAGTCATGACTTTGTACACTCCAAACACTGCTATTGAAGGTGAGGAGAGAAAATTGTGG<br>ACTTCTGACTGTTCCTGTTCCACTAACGGTTCCACCCCAGTTAAGAAGAAGTCCACCGGTGAATACGCC<br>AATTTACCACCACACTTATTAAGATATGATGAAAACTACGATGAAGAAGCTGGTGGTAGACGTAAGGCC<br>TCCTTGAAATGG

Tm	ATGGAGCAAATCCCAGTCTACGAGCGTCCAGGTTTCAACCCACACAAGCAAAACATTACCTTGTTCAAG<br>CATGATGGTTCTACTGTTACTGTCGGTTTGCATGAGTTGGACGCCATGTTCACTCATTCCATCAGAGTT<br>GCTGTCGTCTTCGCCTCTCAAATTGGTGCTTGTGCTTTGTTGTCTGTTATCGTTGCTATGGTCACCAAG<br>AGAGAAAAGAGACGTGCTTTGTTCTTCTTGCACATTATTTCCTTGTTGTTGGTCGTTGTTCGTTCCGTCT<br>TGCAAATCTTGTACTTCGTCGGTCCATGGGCTGAAACTTATAATTACGTCGCCTACTACTATGAAGACAT<br>TCCTTTGTCTGACAAATTGATTTCCATTTGGGCTGGTATTATCCAATTGATTTTGAATATCTGTATTTTGT<br>TATCTTTGATCTTGCAAGTTCGTGTCGTTTACGCCACCTCTCCAAAATTGAACACTATTATGACTTTAGT<br>CTCTTGTGTTATCGCTTCTATTTCTGTCGGTTTCTTCTTTACTGTCATCGTTCAAATTTCTGAGGCTATTT<br>TAAACGGTGTTGGTTACGACGGTTGGGTTTACAAAGTCCATAGAGGTGTCTTCGCTGGTGCTATCGCCT<br>TCTTCTCTTTCATCTTCATCTTTAAGTTGGCCTTCGCTATCAGAAGAAGAAAGGCTTTGGGTTTGCAAAG<br>ATTCGGTCCATTGCAAGTTATCTTCATCATGGGTTGTCAAACTATGATTGTTCCAGCTATCTTTGCTACT<br>TTGGAAAACGGTGTTGGTTTCGAAGGTATGTCCTCTTTGACTGCTACCTTGGCTGTCATTTCCTTACCAT<br>TGTCTTCTATGTGGGCCGCCGCTCAAACCGACGGTCCATCTCCACAATCCACTCCAAGAGACGGTTAT<br>AGAAGATTCTCTACTCGTAGATCTGCCTTGAACAGATCTGACCCATCTGGTGGTAGATCTGTTGACATG<br>AACACCTTGGACTCTACCGGTAACGATTCCTTAGCTTTGCACGTTGATAAGACTTTTACTGTTGAATCTT<br>CCCCATCCTCCCAATCTCAAGCTGGTCCACACAAGGAAAGAGGTTTCGAATTCGCC
Vp1	ATGAGTTCCCAATCACACCCACCGCTAATCGATTTATTTTACGATTCCAGTTATGACCCTGGTGAAAGTT<br>TAATTTATTACACATCCATCTATGGTAATAATACATACATAACTTTTGATGAACTCCAGACGATAGTGAAC<br>AAGAAGGTCACACAAGGTATCTTATTTGGTGTCAGATGTGGTGCTGCTTTCCTGATGTTGGTAGCAATG<br>TGGTTGATTTCCAAAAATAAAAGATCTAGAATTTTCATTACCAACCAATGTTGTCTGGTCTTCATGATAAT<br>GCATTCTGGTCTTTATTTTAGGTACCTGCTTTCAAGGTACGGTTCAGTTACTTTCATTCTAACAGGGTTC<br>CAACAACTGCTTACAAGAAATGACATTCATATTTATGGAGCTACTGATTTTATCCAAGTAGCTTTGGTAG<br>CTTGCATAGAATTATCTCTTATTTTCCAAATAAAAGTGATATTCGCTGGTACAAACTATGGTAAGTTGGCT<br>AATTATTTCATCACTCTAGGTTCATTATTGGGTTTAGCCACCTTTGGTATGTACATGCTTACTGCTATTAA<br>CGGTACAATAAAATTATACAATAACGAATATGACCCAAACCAAAGGAAATACTTTAACATTTCTACAATAT<br>TGCTTGCATCATCAATTAATATGCTAACGCTGATACTTATATTGAAGCTGGTGGCAGCAATTAGAACAAG<br>ACGTTACTTAGGTTTGAAGCAATTCGATAGTTTTCACATCCTATTAATCATGTCGACTCAAACATTAATAA<br>TTCCTTCTATCTTATTTATTCTATCATACAGTTTGAGAGAGGATATGCATACTGATCAATTAATAATCATC<br>GGAAATCTGATCGTGGTATTGTCATTACCATTGTCCTCAATGTGGGCTTCGTCTCTAAACAATTCAAGTA<br>AACCTACATCTTTGAATACTGATTTCTCAGGGCCAAAATCAAGTGAAGAAGGGACAGCAATAAGTTTGC<br>TATCACAAAACATGGAACCATCAATAGTCACTAAATATACAAGAAGATCACCTGGGTTATACCCAGTAAG<br>CGTGGGTACACCAATTGAAAAAGAAGCATCATACACTCTTTTTGAAGCTACTGACATTGATTTTGAAAGC<br>AGTAGTAACGATATCACAAGGACTTCA
Vp2	ATGTCAGGAATTGATGATATGGGTGATAAACCAGATATTTTAGGTTTATTTTATGATGCTAACTATGATC<br>CAGGTCAAGGTATACTCACATTTATTTCAATGTACGGGAATACTACTATAACTTTTGATGAGTTACAGTT<br>AGAGGTCAATAGTTTAATTACAAGTGGTATTATGTTCGGCGTCAGATGTGGTGCTGCTTGTTTGACATT<br>GTTAATAATGTGGATGATTTCTAAGAATAAGAAGACTCCAATTTTTATTATTAATCAATGCTCGCTAATCC<br>TTATTATTATGCATTCAGGTTTATATTTTAAGAATATTCTATCAAATTTGAATTCTTTATCATATATCTTAAC<br>TGGGTTTACTCAAAATATCACTAAAAATAATATACATGTCTTTGGTGCCGCTAATATTATTCAAGTTTTAT<br>TAGTAGCAACCATTGAACTGTCGTTAGTGTTTCAAATTCGAGTCATGTTTAAAGGTGACAGTTTTAGAAA<br>AGCTGGTTACGGTTTGTTGTCAATTGCGTCTGGTTTGGGTATAGCTACTGTCGTCATGTATTTTTACTCT<br>GCCATTACAAATATGATTGCTGTTTATAATCAAACTTACAACTCCACTGCTAAATTATTTAACGTTGCAAA<br>CATTCTTCTGTCTACATCGATAAATTTTATGACGGTAGTATTAATTGTTAAATTATTTTTGGCTGTTAGAT<br>CAAGAAGATATTTGGGTTTAAAGCAGTTCGATAGTTTCCATATTTTATTGATTATGTCATGTCAAACATTG<br>ATTGTACCATCAATTCTTTTTATCTTATCATACGCTTTAAGTACTAAGCTGTACACTGATCATTTAGTTGT<br>CATTGCAACTTTATTAGTCGTTCTATCTTTACCATTATCTTCGATGTGGGCAAGCGCTGCAAATAATTCT<br>CCTAAACCAAGCTCGTTTACAACCGATTATTCAAACAAGAATCCTAGTGACACACCAAGCTTCTACAGTC<br>AAAGTATTAGTTCCTCGATGAAAAGCAAATTCCCAAGCAAATTCATACCCTTCAATTTCAAGTCTAAAGA<br>CAATTCTTCTGACACTAGATCAGAAAATACATATATTGGCAATTATGACATGGAAAAGAATGGATCACCA<br>AATCACTCTTATTCTTCCAAAGATCAAAGTGAAGTTTACACTATAGGTGTAAGCTCTATGCACACAGATA<br>TAAAGTCACAAAAGAATATCAGTGGACAGCATTTATATACCCCAAGTACAGAGATTGATGAAGAAGCTA<br>GAGACTTCTGGGCGGGCAGAGCTGTTAATAATTCAGTTCCAAATGACTATCAACCATCTGAGTTACCAG<br>CATCGATTCTTGAAGAATTGAATTCACTGGATGAAAATAATGAAGGTTTCTTGGAGACAAAAAGAATAAC<br>ATTTAGAAAACAA

Yl	ATGCAATTGCCACCACGTCCAGACTTCGACATTGCCACTTTGGTTGCCTCTATCACTGTTCCAGAAACT<br>GAATTGGTCTTGGGTCAAATGCCATTGGGTGCTTTAGAACAATTGTACCAAAACAGATTGCGTTTGGCT<br>ATTTTGTTCGGTGTCAGAGTCGGTGCTGCTGTTTTGACCTTGATTGCTATGCACTTAATCTCCAAGAAGA<br>ACAGAACCAAGATCTTGTTCTTGGCTAACCAAATGTCTTTGATCATGTTGATCATCCATGCTGCTTTGTA<br>CTTCAGATTCTTGTTGGGTCCATTCGCCTCCATGTTGATGATGGTTGCTTACATCGTTGATCCAAGATCT<br>AACGTCTCTAACGATATCTCTGTTTCTGTTGCCACCAACGTTTTCATGATGTTGATGATTATGTCCGTCC<br>AATTGTCTTTGGCTGTTCAAACCCGTTCTGTTTTCCACGCTTGGTTGAAGTCTCGTATTTACGTTACCGT<br>TGGTTTAATCTTGTTGTCCTTGGTCGTCTTCGTCTTCTGGACCACCCACACTATCGTTTCTTGTATCGTT<br>TTAACCCATCCAACTAGAGACTTGCCATCTATGGGTTGGACTAGATTAGCTTCTGACGTTTCCTTCGCTT<br>GTTCTATCTCTTTCGCTTCTTTGGTCTTGTTGGCTAAGTTGGTCACCGCCATCAGAGTTAGAAAGACCTT<br>GGGTAAGAAGCCATTGGGTTACACCAAGGTTTTGGTCATCATGTCCACTCAATCTTTAGTCGTTCCATC<br>TATCTTGATTATCGTTAACTACGCTTTGCCAGAAAAAAACTCTTGGATCTTGTCTGGTGTCGCTTACTTG<br>ATGGTTGTTTTGTCCTTACCATTGTCCTCCATTTGGGCTACCGCCGTCCATGACGACGAAATGCAATCC<br>AACTACTTGTTGTCTGCCTTGAAAGATGGTCACGTTCAACCATCCGAATCTAAGTTGAAGACTGTTTTCT<br>TGAACAGATTGAGACCATTCTCTACTACCACTAACAGAGACGATGAATCCTCTGTTGATTCCCCAGCCA<br>TGCCATCTCCAGAATCTGATGTTACCTTCTTGAACACTGGTTTCGAATGTGACGAAAAGATG
Zb	Sequence reported[11]
Zr	Sequence reported[11]

Supplementary Table 4 – Strains used in this study.

Strain name	Genotype	Comment	Reference
BY4741	*MAT***a** *leu2Δ0 met15Δ0 ura3Δ0 his3Δ1*	Parent of yNA899	[12]
BY4742	*MATα lys2Δ0 leu2Δ0 ura3Δ0 his3Δ1*	Parent of yNA903	[12]
yNA899	*MAT***a** *leu2Δ0 met15Δ0 ura3Δ0 his3Δ1 MFa1Δ MFa2Δ MFalpha1Δ MFalpha2Δ ste2Δ ste3Δ sst2Δ far1Δ bar1Δ*	Parent of JTy014	This study
yNA903	*MATα lys2Δ0 leu2Δ0 ura3Δ0 his3Δ1 MFa1Δ MFa2Δ MFalpha1Δ MFalpha2Δ ste2Δ ste3Δ sst2Δ far1Δ bar1Δ*	Used for validation of language functionality in α-type strain	This study
JTy014	*MAT***a** *leu2Δ0 met15Δ0 ura3Δ0 his3Δ1 MFa1Δ MFa2Δ MFalpha1Δ MFalpha2Δ ste2Δ ste3Δ sst2Δ far1Δ bar1Δ HO::FUS1p-coRFP-LEU2*	Used for GPCR characterization after transformation with the GPCR expression constructs. Parent of ySB98/99/100	This study
JTy015	*MAT***a** *leu2Δ0 met15Δ0 ura3Δ0 his3Δ1 MFa1Δ MFa2Δ MFalpha1Δ MFalpha2Δ ste2Δ ste3Δ sst2Δ far1Δ bar1Δ HO::FIG1p-coRFP-LEU2*		This study
ySB98	*MAT***a** *leu2Δ0 met15Δ0 ura3Δ0 his3Δ1 MFa1Δ MFa2Δ MFalpha1Δ MFalpha2Δ ste2Δ ste3Δ sst2Δ far1Δ bar1Δ HO::FUS1p-coRFP-LEU2 ste2::TDH3p-Ca.Ste2-STE2t*	Ca.Ste2,Sc.Ste2 or Bc.Ste2 under control of the constitutive *TDH3* promoter integrated into the Ste2 locus. Used for single cell analysis and GPCR activation-deactivation experiments	This study
ySB99	*MAT***a** *leu2Δ0 met15Δ0 ura3Δ0 his3Δ1 MFa1Δ MFa2Δ MFalpha1Δ MFalpha2Δ ste2Δ ste3Δ sst2Δ far1Δ bar1Δ HO::FUS1p-coRFP-LEU2 Ste2::TDH3p-Sc.Ste2-STE2t*		This study
ySB100	*MAT***a** *leu2Δ0 met15Δ0 ura3Δ0 his3Δ1 MFa1Δ MFa2Δ MFalpha1Δ MFalpha2Δ ste2Δ ste3Δ sst2Δ far1Δ bar1Δ HO::FUS1p-coRFP-LEU2 Ste2::TDH3p-Bc.Ste2-STE2t*		This study
ySB265	*MAT***a** *leu2Δ0 met15Δ0 ura3Δ0 his3Δ1 MFa1Δ MFa2Δ MFalpha1Δ MFalpha2Δ ste2Δ ste3Δ sst2Δ far1Δ bar1Δ*	Ste12 replaced by Ste12*. *TDH3*p-Bc.Ste2, Ca.Ste2 or Vp1.Ste2 integrated into the *STE2* locus. *SEC4* under control of *OSR1* promoter and insulated by an upstream *CYC1* terminator or under control of the *OSR4* promoter without	This study

	ste12::ste12 ste2::TDH3p-Bc.Ste2 sec4::CYC1t-OSR1p-Sec4*	insulation. Used for rendering strains dependent on peptide sensing.	
ySB270	*MAT**a** leu2Δ0 met15Δ0 ura3Δ0 his3Δ1 MFa1Δ MFa2Δ MFalpha1Δ MFalpha2Δ ste2Δ ste3Δ sst2Δ far1Δ bar1Δ ste12::ste12* ste2::TDH3p-Ca.Ste2 sec4::OSR4p-Sec4*		This study
ySB188	*MAT**a** leu2Δ0 met15Δ0 ura3Δ0 his3Δ1 MFa1Δ MFa2Δ MFalpha1Δ MFalpha2Δ ste2Δ ste3Δ sst2Δ far1Δ bar1Δ ste12::ste12* ste2::TDH3p-Vp1.Ste2 sec4::OSR4p-Sec4*		This study
yJB416	*MAT**a** leu2Δ0 met15Δ0 ura3Δ0 his3Δ1 MFa1Δ MFa2Δ MFalpha1Δ MFalpha2Δ ste2Δ ste3Δ sst2Δ far1Δ bar1Δ ste2::TDH3p-Kp.Ste2*	Parent GPCR integration strains for constructing the 2-yeast linker strains, ring, bus -and tree topologies; derived from yNA899.	This study
yJB418	*MAT**a** leu2Δ0 met15Δ0 ura3Δ0 his3Δ1 MFa1Δ MFa2Δ MFalpha1Δ MFalpha2Δ ste2Δ ste3Δ sst2Δ far1Δ bar1Δ ste2::TDH3p-Cl.Ste2*		This study
yJB421	*MAT**a** leu2Δ0 met15Δ0 ura3Δ0 his3Δ1 MFa1Δ MFa2Δ MFalpha1Δ MFalpha2Δ ste2Δ ste3Δ sst2Δ far1Δ bar1Δ ste2::TDH3p-Cgu.Ste2*		This study
yJB422	*MAT**a** leu2Δ0 met15Δ0 ura3Δ0 his3Δ1 MFa1Δ MFa2Δ MFalpha1Δ MFalpha2Δ ste2Δ ste3Δ sst2Δ far1Δ bar1Δ ste2::TDH3p-Bc.Ste2*		This study
yJB423	*MAT**a** leu2Δ0 met15Δ0 ura3Δ0 his3Δ1 MFa1Δ MFa2Δ MFalpha1Δ MFalpha2Δ ste2Δ ste3Δ sst2Δ far1Δ bar1Δ ste2::TDH3p-Ca.Ste2*		This study
yJB523	*MAT**a** leu2Δ0 met15Δ0 ura3Δ0 his3Δ1 MFa1Δ MFa2Δ MFalpha1Δ MFalpha2Δ ste2Δ ste3Δ sst2Δ far1Δ bar1Δ ste2::TDH3p-Hj.Ste2*		This study
ySB315	*MAT**a** leu2Δ0 met15Δ0 ura3Δ0 his3Δ1 MFa1Δ MFa2Δ MFalpha1Δ MFalpha2Δ ste2Δ ste3Δ sst2Δ far1Δ bar1Δ ste2::TDH3p-Cl.Ste2 ste3::TDH3p-Sj.Ste2*	Strain encoding two GPCRs for the implementation of branches in the tree-topologies. Derived from yJB418	This study
ySB316	*MAT**a** leu2Δ0 met15Δ0 ura3Δ0 his3Δ1 MFa1Δ*	Strain encoding two GPCRs for the implementation of branches in the tree-	This study

	MFa2Δ MFalpha1Δ MFalpha2Δ ste2Δ ste3Δ sst2Δ far1Δ bar1Δ ste2::TDH3p-Bc.Ste2 ste3::TDH3p-So.Ste2	topologies. Derived from yJB422	

Supplementary Table 5 – peptide/GPCR pair characteristics. Parameters were extracted from the dose response curves given in **Supplementary Figure 4** by fitting them to a four-parameter model using Prism GraphPad. Errors represent the standard error of the curve generated from triplicate values, except for fold change error, which was propagated from the Top and Bttm errors. Peptide/GPCR pairs are ordered alphabetically according to the two-letter species code.

Code	EC50	EC50 error	Top	Top error	Bttm	Bttm error	Span	Span error	Fold Change	Fold Change error	Hill Slope	Hill Slope error
Bb	-8.5	0.0	244.1	2.5	25.2	2.8	218.9	3.9	9.7	1.1	1.0	0.1
Bc	-8.1	0.1	351.9	5.8	28.6	5.3	323.3	8.6	12.3	2.3	0.7	0.1
Bm	-6.7	0.1	158.8	3.3	30.3	1.9	128.4	3.9	5.2	0.3	1.2	0.2
Ca	-7.7	0.0	271.6	3.8	38.9	3.1	232.8	5.1	7.0	0.6	1.0	0.1
Cau	-8.1	0.1	336.9	6.7	50.6	6.2	286.3	9.8	6.7	0.8	0.8	0.1
Cg	-5.9	0.0	213.6	4.0	30.5	1.9	183.0	4.5	7.0	0.5	2.4	0.5
Cgu	-7.4	0.0	211.7	2.7	41.2	2.0	170.5	3.5	5.1	0.3	1.1	0.1
Cl	-7.5	0.1	225.8	4.4	39.8	3.2	186.0	5.8	1.4	0.1	0.9	0.1
Cn	-7.4	0.1	152.2	4.2	29.7	3.0	122.5	5.4	5.1	0.5	1.1	0.2
Cp	-8.5	0.0	254.0	2.7	36.2	3.0	217.8	4.3	7.0	0.6	0.8	0.1
Ct	-8.2	0.2	166.7	10.1	32.0	10.0	134.6	14.7	5.2	1.6	1.2	0.6
Fg	-7.1	0.0	232.2	2.5	29.2	1.6	203.0	3.0	8.0	0.4	1.3	0.1
Gc	-6.9	0.0	187.2	2.8	22.9	1.8	164.3	3.4	8.2	0.7	1.8	0.2
Hj	-7.8	0.1	429.5	9.3	53.0	7.3	376.5	13.2	8.1	1.1	0.6	0.1
Kl	-7.3	0.0	223.1	2.8	37.2	1.8	185.9	3.6	6.0	0.3	0.8	0.0
Kp	-8.2	0.1	269.1	4.4	44.8	4.2	224.3	6.5	6.0	0.6	0.8	0.1
Le	-7.7	0.1	412.5	6.4	22.9	4.7	389.6	8.8	18.0	3.7	0.7	0.1
Mo	-5.3	0.1	97.6	5.5	29.9	1.0	67.7	5.7	3.3	0.2	1.2	0.2
Nc	-6.3	0.1	286.7	6.4	27.6	1.7	259.2	7.2	10.4	0.7	0.6	0.0
Pb	-6.0	0.1	217.1	9.3	20.2	1.6	196.9	10.1	10.8	1.0	0.5	0.0
Pd	-7.7	0.1	190.0	5.2	28.8	4.0	161.2	7.2	6.6	0.9	0.7	0.1
Pr	-5.8	0.1	207.3	7.3	27.9	1.1	179.4	7.7	7.4	0.4	0.6	0.0
Sc	-8.9	0.0	253.1	2.2	36.2	2.8	217.0	3.8	7.0	0.5	1.0	0.1
Sca	-8.1	0.0	155.4	1.9	24.3	1.7	131.1	2.8	6.4	0.5	0.7	0.1
Sj	-7.8	0.0	311.3	3.7	21.2	3.1	290.0	5.1	14.7	2.2	1.2	0.1
So	-7.8	0.1	263.4	6.2	23.7	5.5	239.7	5.5	11.1	2.6	1.5	0.4
Sp	-6.2	0.2	224.3	16.7	29.6	3.9	194.7	3.9	7.6	1.1	0.5	0.1
Ss	-7.9	0.1	318.0	5.0	23.0	4.4	295.0	7.0	13.8	2.6	0.9	0.1
Vp1	-8.6	0.0	243.1	1.7	28.8	1.9	214.2	2.6	8.4	0.5	1.4	0.1
Vp2	-7.7	0.0	215.2	1.8	28.0	1.5	187.2	2.4	7.7	0.4	1.1	0.1
Zb	-5.8	0.0	292.5	3.5	39.1	1.3	253.4	3.9	7.5	0.3	1.7	0.1
Zr	-7.4	0.1	109.9	1.4	57.2	1.2	52.7	1.9	1.9	0.0	2.4	0.6

Supplementary Table 6 – DNA sequences of peptide ligand expression cassettes. Peptide expression cassettes were cloned into vector pRS423 under control of the constitutive *ADH1* promoter or the peptide inducible *FUS1*p promoter. The first row shows the amino acid sequence of the designed generic peptide ligand precursor. The second row shows its DNA sequence. This precursor was used to clone in all other peptide ligand sequences. The sequences were ordered as oligonucleotides codon-optimized for expression in yeast and inserted into the cassette by Gibson assembly[1]. The secretion signal is highlighted in green, the Kex2 processing site is marked in bold grey, the Ste13 processing site encoding sequence is marked in bold. Peptide sequences are ordered alphabetically according to their 2-letter species code.

**Amino acid sequence of peptide precursors**
RFPSIFTAVLFAASSALAAPVNTTTEDETAQIPAEAVIGYLDLEGDFDVAVLPFSNSTNNGLLFINTTIASIAAKEEGVSLD **KR(EAEA)-**peptide-Stop
**DNA sequence of peptide pre-pro precursor**
**Without Ste13 processing site (EAEA)**
AGATTTCCTTCAATTTTTACTGCAGTTTTATTCGCAGCATCCTCCGCATTAGCTGCTCCAGTCAACACTACAACAG AAGATGAAACGGCACAAATTCCGGCTGAAGCTGTCATCGGTTACTTAGATTTAGAAGGGGATTTCGATGTTGCTG TTTTGCCATTTTCCAACAGCACAAATAACGGGTTATTGTTTATAAATACTACTATTGCCAGCATTGCTGCTAAAGAA GAAGGGGTATCTTTGGAT**AAAAGA** – peptide sequence - TAG
**Plus Ste13 processing site**
AGATTTCCTTCAATTTTTACTGCAGTTTTATTCGCAGCATCCTCCGCATTAGCTGCTCCAGTCAACACTACAACAG AAGATGAAACGGCACAAATTCCGGCTGAAGCTGTCATCGGTTACTTAGATTTAGAAGGGGATTTCGATGTTGCTG TTTTGCCATTTTCCAACAGCACAAATAACGGGTTATTGTTTATAAATACTACTATTGCCAGCATTGCTGCTAAAGAA GAAGGGGTATCTTTGGAT**AAAAGAGAGGCTGAAGCT**- peptide sequence - TAG

**Code**	**DNA sequence**
Bb	ggtgtatgagaccaggtcaaccatgttgg
Bc	tggtgtggtagaccaggtcaaccatgt
Ca	ggtttcagattgaccaacttcggttacttcgaaccaggt
Cgu	aagaagaactctagattcttgacctactggttcttccaaccaatcatg
Cl	aagtggaagtggatcaagttcagaaacaccgacgttatcggtTAG
Gc	ggtgactggggttggttctggtacgttccaagaccaggtgacccagctatg
Hj	tggtgttacagaatcggtgaaccatgttgg
Kp	cagatggagaaacaacgaaaagaaccaaccattcggt
Le	ggatgtggaccagatacggtagattctctccagtt
Pb	ggtgtaccagaccaggtcaaggttgt
Pd	ttctgttggagaccaggtcaaccatgtggt
Sc	tggcactggttgcaattgaagccaggtcaaccaatgtac
Sj	gtttctgacagagttaagcaaatgttgtctcactggtggaacttcagaaacccagacaccgctaacttg
So	acctacgaagacttcttgagagtttacaagaactggtggtctttccaaaacccagacagaccagacttg
Vp	tggcactggttggaattggacaacggtcaaccaatctac
Zr	cacttcatcgaattggacccaggtcaaccaatgttc

Supplementary Table 7 – gRNAs used for genome engineering

Target gene or locus	gRNA Sequence	
	**5' gRNA**	**3' gRNA**
STE2	CAGAATCAAAAATGTCTGATG	ATGAGGAAGCCAGAAAGTT
STE3	CATACAAGTCAGCAATAATA	ATAGTTCAGAAAATACTGC
MFalpha1	AAAACTGCAGTAAAAATTGA	ATTGGTTGCAGTTAAAACC
MFalpha2	CGCTAAAATAAAAGTGAGAA	ACTGGTTGCAACTCAAGCC
MFa1	AAAGACCAGCAGTGAAAAGA	
MFa2	TTCCACACAAGCCACTCAGA	
FAR1	AAAATACACACTCCACCAAG	GCAAAGAATTCATCAGACCC
BAR1	TCTTTGTTTGAAACTTATTT	TTGTACATGAAACTAAATAT
SST2	GTAAGATGGTGGATAAAAAT	CATCTTTGTATACGTCTGAC
STE12	AATAACCAATAGTAGAACAG	CTGTTCTACTATTGGTTATT
ΔSTE2 (insertion of *TDH3*p-xySte2)	ATATTCAAGATTTTTTTCTG	
ΔSTE3 (insertion of *TDH3*p-xySte2)	ATGTGTAAATGAAGGAATAA	
STE12 (replacement by Ste12*)	TGAAGTCAGTAAAGCTACTC	
SEC4 (replacement of SEC4 promoter by OSRs)	TCCTCGTGGGCCAGGACTAG	
SEC4 (replacement of *SEC4* promoter by *CYCt*-OSRs)	CATTCTACCTCTAGGGAAGC	

Supplementary Table 8 – Amino acid sequences of GPCRs

Code	Sequence
Sc	MSDAAPSLSNLFYDPTYNPGQSTINYTSIYGNGSTITFDELQGLVNSTVTQAIMFGVRCGAAALTLIVMW<br>MTSRSRKTPIFIINQVSLFLIILHSALYFKYLLSNYSSVTYALTGFPQFISRGDVHVYGATNIIQVLLVASIETS<br>LVFQIKVIFTGDNFKRIGLMLTSISFTLGIATVTMYFVSAVKGMIVTYNDVSATQDKYFNASTILLASSINFMS<br>FVLVVKLILAIRSRRFLGLKQFDSFHILLIMSCQSLLVPSIIFILAYSLKPNQGTDVLTTVATLLAVLSLPLSSM<br>WATAANNASKTNTITSDFTTSTDRFYPGTLSSFQTDSINNDAKSSLRSRLYDLYPRRKETTSDKHSERTF<br>VSETADDIEKNQFYQLPTPTSSKNTRIGPFADASYKEGEVEPVDMYTPDTAADEEARKFWTEDNNNL
Scas1	MSDAPPPLSELFYNSSYNPGLSIISYTSIYGNGTEVTFNELQSIVNKKITEAIMFGVRCGAAILTIIVMWMISK<br>KKKTPIFIINQVSLFLILLHSAFNFRYLLSNYSSVTFALTGFPQFIHRNDVHVYAAASIFQVLLVASIEISLMFQ<br>IRVIFKGDNFKRIGTILTALSSSLGLATVAMYFVTAIKGIIATYKDVNDTQQKYFNVATILLASSINFMTLILVIK<br>LILAIRSRRFLGLKQFDSFHILLIMSFQSLLAPSILFILAYSLDPNQGTDVLVTVATLLVVLSLPLSSMWATAA<br>NNASRPSSVGSDWTPSNSDYYSNGPSSVKTESVKSDEKVSLRSRIYNLYPKSKSEFEQSSEHTYVDKV<br>DLENNFYELSTPITERSPSSIIKKGKQGISTRETVKKLDSLDDIYTPNTAADEEARKFWSEDVSNELDSLQK<br>IETETSDELSPEMLQLMIGQEEEDDNLLATKKITVKKQ
Vp2	MSGIDDMGDKPDILGLFYDANYDPGQGILTFISMYGNTTITFDELQLEVNSLITSGIMFGVRCGAACLTLLI<br>MWMISKNKKTPIFIINQCSLILIIMHSGLYFKNILSNLNSLSYILTGFTQNITKNNIHVFGAANIIQVLLVATIELS<br>LVFQIRVMFKGDSFRKAGYGLLSIASGLGIATVVMYFYSAITNMIAVYNQTYNSTAKLFNVANILLSTSINFM<br>TVVLIVKLFLAVRSRRYLGLKQFDSFHILLIMSCQTLIVPSILFILSYALSTKLYTDHLVVIATLLVVLSLPLSS<br>MWASAANNSPKPSSFTTDYSNKNPSDTPSFYSQSISSSMKSKFPSKFIPFNFKSKDNSSDTRSENTYIGN<br>YDMEKNGSPNHSYSSKDQSEVYTIGVSSMHTDIKSQKNISGQHLYTPSTEIDEEARDFWAGRAVNNSVP<br>NDYQPSELPASILEELNSLDENNEGFLETKRITFRKQ
Vp1	MSSQSHPPLIDLFYDSSYDPGESLIYYTSIYGNNTYITFDELQTIVNKKVTQGILFGVRCGAAFLMLVAMWL<br>ISKNKRSRIFITNQCCLVFMIMHSGLYFRYLLSRYGSVTFILTGFQQLLTRNDIHIYGATDFIQVALVACIELS<br>LIFQIKVIFAGTNYGKLANYFITLGSLLGLATFGMYMLTAINGTIKLYNNEYDPNQRKYFNISTILLASSINML<br>TLILILKLVAAIRTRRYLGLKQFDSFHILLIMSTQTLIIPSILFILSYSLREDMHTDQLIIIGNLIVVLSLPLSSMWA<br>SSLNNSSKPTSLNTDFSGPKSSEEGTAISLLSQNMEPSIVTKYTRRSPGLYPVSVGTPIEKEASYTLFEAT<br>DIDFESSSNDITRTS
Td	MSDSAQNLSDLAFNSSYNPLDSFITFTSIYGDNTAVKFSVLQDMVDVNTNEAIVYGTRCGASVLTQIIMW<br>MISKNRRTPVFIINQVSLTLILIHSALYFKYLLSGFGSVVYGLTAFPQLIKPGDLRAFAAANIVMVLLVASIEA<br>SLIFQVKVIFTGDNMKRVGLILTIICTCMGLATVTMYFITAVKSIVSLYRDMSGSSTVLYNVSLIMLASSIHFM<br>ALILVVKLFLAVRSRRFLGLKQFDSFHILLIISCQTLLVPSLLFIIAYSFPSSKNIESLKAIAVLTVVLSLPLSSM<br>WATAANNFTNSSSSGSDSAPTNGGFYGRGSSNLYPEKTDNRSPKGARNALYELRSKNNAEGQADIYTV<br>TDIENDIFNDLSKPVEQNIFSDVQIIDSHSLHKACSKEDPVMTLYTPNTAIEGEERKLWTSDCSCSTNGST<br>PVKKKSTGEYANLPPHLLRYDENYDEEAGGRRKASLKW
Sk	MSGKQDLSPLGLYSSYDPTKGLISYTSLYGSGTTVTFEELQIFVNKKITQGILFGTRIGAAGLAIIVLWMVS<br>KNRKTPIFIINQISLFLILLHSSLFLRYLLGDYASVVFNFTLFSQSISRNDVHVYGATNMIQVLLVAAVEISLIF<br>QVRVIFKGDSYKGVGRILTSISAVLGFTTVVMYFITAVKSMTSVYSDLTKTSDRYFFNIASILLSSSVNFMTL<br>LLTVKLILAVRSRRFLGLKQFDSFHVLLIMSFQTLIFPSILFILAYALNPNQGTDTLTSIATLLVTLSLPLSSM<br>WATSANNSSHPSSINTQFRQRNYDDVSFKTGITSFYSESSKPSSKYRHTNNLYDLYPVSRTSNSRCNGY<br>PNDGSKLAPNPNCVGHNGSTMSVNDKNGAHATCVQNNVTLNTDSTLNYSNVDTQDTSKILMTT
Kl	MSEEIPSLNPLFYNETYNPLQSVLTYSSIYGDGTEITFQQLQNLVHENITQAIIFGTRIGAAGLALIIMWMVS<br>KNRKTPIFIINQSSLVLTIVQSALYLSYLLSNFGGVPFALTLFPQMIGDRDKHLYGAVTLIQCLLVACIEVSLV<br>FQVRVIFKADRYRKIGIILTGVSASFGAATVAMWMITAIKSIIVVYDSPLNKVDTYYYNIAVILLACSINFITLLL<br>SVKLFLAFRARRHLGLKQFDSFHILLIMSTQTLIGPSVLYILAYALNNKGVKSLTSIATLLVVLSLPLTSIWAA<br>AANDAPSASTFYRQFNPYSAQNRDDSSSYSYGKAFSDKYSFSNSPQTSDGCSSKELELSTQLEMDLES<br>GESFMDRAKRSDFVSSPGSTDATVIKQLKASNIYTSETDADEEARAFWVNAIHENKDDGLMQSKTVFKE<br>LR
Zr	MSEINNSTYNPMNAYVTFTSIYGDDTMVRFKDVELVVNKRVTEAIMFGVKVGAASLTLIIMWMISKKRTTP<br>IFIINQSSLVFTIIHASLYFGYLLSGFGSIVYNMTSFPQLISSNDVRVYAATNIFEVLLVASIEISLVFQVKVMF<br>ANNNGRRWTWCLMVVSIGMALATVGLYFATAVELIRAAYSNDTVSRHVFYNVSLILLASSVNLMTLMLVV<br>KLVLAIRSRRFLGLKQFDSFHILLIMSCQTLIAPSILFILGWTLDPHTGNEVLITVGQLLIVLSLPLSSMWATT<br>ANNTSSSSSSVSCNDSSFGNDNLCSKSSQFRRTFMNRFRPKSVNGDGNSENTFVTIDDLEKSVFQELST<br>PVSGESKIDHDHASSISCQKTCNHVHASTVNSDKGSWSSDGSCGSSPLRKTSTVNSEDLPPHILSAYDD<br>DRGIVESKKIILKKL

Zb	MSGLANNTSYNPLESFIIFTSVYGGDTMVKFEDLQLVFTKRITEGILFGVKVGAASLTMIVMWMISRRRTS<br>PIFIMNQLSLVFTILHASFYFKYLLDGFGSIVYTLTLFPQLITSSDLHVFATANVVEVLLVSSIEASLVFQVNV<br>MFAGSNHRKFAWLLVGFSLGLALATVALYFVTAVKMIASAYASQPPTNPIYFNVSLFLLAASVFLMTLMLT<br>VKLILAIRSRRFLGLKQFDSFHILLIMSCQTLIAPSVLYILGFILDHRKGNDYLITVAQLLVVLSLPLSSMWAT<br>TANDASSGTSMSSKESVYGSDSLYSKSKCSQFTRTFMNRFSTKPTKNDEISDSAFVAVDSLEKNAPQGI<br>SEHVCEFPQSDLSDQATSISSRKKEAVVYASTVDEDKGSFSSDINGYTVTNMPLASAASANCENSPCHV<br>PRPYEENEGVVETRKIILKKNVKW
Cg	MEMGYDPRMYNPRNEYLNFTSVYDVNDTIRFSTLDAIVKGLLRIAIVHGVRLGAIFMTLIIMFISSNTWKKPI<br>FIINMVSLMLVMIHSALSFHYLLSNYSSISYILTGFPQLITSNNKRIQDAASIVQVLLVAAIEASLVFQIHVMFT<br>IENIKLIREIVLSISIAMGLATVATYLAAAIKLIRGLHDEVMPQTHLIFNLSIILLASSINFMTFILVIKLFFAIRSRR<br>YLGLRQFDAFHILLIMFCQSLLIPSVLYIIVYAVDSRSNQDYLIPIANLFVVLSLPLSSIWANTSNNSSRSPKY<br>WKNSQTNKSNGSFVSSISVNSDSQNPLYKKIVRFTSKGDTTRSIVSDSTLAEVGKYSMQDVSNSNFECR<br>DLDFEKVKHTCENFGRISETYSELSTLDTTALNETRLFWKQQSQCDK
Ag	MGEEVSSFVEQYYDPNYDPSQSMLTYMSKFSNESTIKFEDLQEYINENVMLGVFTGAKIAAAALALIILWM<br>VTKRKRTPIYIVNQISLLLTVIHGILVLSGLLGGFSSSIFTLTLFPQCVNRSDIRLFVATNISMVSLIASIQVSLV<br>LQVHVIFRAGTHRRLGIFLTAVSAIIGFTTVCFYLVSAVLSVMAVYQDIDNIGDTFFLSIAYICMAISVNFIFLL<br>LSVKLLLAIRLRRFLGLKQFDGLHILFIMSTQTIICPSILFILAFACEKNITDSLVYIAVLLVSLSLPLSSVWATA<br>ANNATVPPFLNAHSLTSRYKAESWYTDSKNDAGSFSSSENCGSGYRHGRYSNNGGSSPHQCTGGDNT<br>VIDIEKCQYRVNPTPHTSGQFAFNQDSLETEFSEDTVVQIRTPNTEVEEEAKIFWARASITHENSSSGVEC<br>GAHDMQTNVFKTPTSQTGSDCN
Ss	MDTSINTLNPANIIVNYTLPNDPRVISVPFGAFDEYVNQSMQKAIIHGVSIGSCTIMLLIILIFNVKRKKSPAF<br>YLNSVTLTAMIIRSALNLAYLLGPLAGLSFTFSGLVTPETNFSVSEATNAFQVIVVALIEASMTFQVFVVFQS<br>PEVKKLGIALTSISAFTGAAAVGFTINSTIQQSRIYHSVVNGTPTPTVATWSWVRDVPTILFSTSVNIMSFILI<br>LKLGFAIKTRRYLGLRQFGSLHILLMMATQTLLAPSILILVHYGYGTSSNSQLILISYLLVVLSLPVSSIWAAT<br>ANNSPQLPSSATLSFMNKTTSHFSES
Kp	MEEYSDSFDPSQQLLNFTSLYGETDATFAELDDYHFYVVKYAIVYGARIGVGMFCTLMLFVVSKSWKTPI<br>FVLNQSSLILLIIHSGFYIHYLTNQFSSLTYMFTRIPNETHAGVDLRINVVTNTLYALLILSIEISLIYQVFVIFKG<br>VYENSLRWIVTIFTALFAAAVVAINFYVTTLQSVSMYNSNVDFPRWASNVPLILFASSVNWACLLLSLKLFF<br>AIKVRRSLGLRQFDTFHILAIMFSQTLIIPSILIVLGYTGTRDRDSLASLGFLLIVVSLPFSSMWAATANNSNI<br>PTSTGSFAWKNRYSPSTYSDDTTAVSKSFTIMTAKDECFTTDTEGSPRFIKGDRTSEDLHF
Cgu	MKSCSIGFGIPFINEPNFETVSILTMDVSFIDADVNPDNILLNFTIPGYQNGFSVPMVVINELQKSQMKYAIV<br>YGCGVGASLILLFVVWILCSRKTPLFIMNNIPLVLYVISSSLNLAYITGPLSSVSVFLTGILTSHDAINVVYAS<br>NALQMLLIFSIQSTMAYHVYVMFKSPQIKYLRYMLVGFLGCLQIVTTCLYINYNVLYSRRMHKLYETGQTY<br>QDGTVMTFVPFILFQCSVNFSSIFLVLKLIMAIRTRRYLGLRQFGGFHILMIVSLQTMLVPSILVLVNYAAHK<br>AVPSNLLSSVSMMIIVLSLPASSMWAAAANASSAPSSAASSLFRYTTSDSDRTLETKSDHFIMKHESHNS<br>SPNSSPLTLVQKRISDATLELPKELEDLIDSTSI
Cp	MNKIVSKLSSSDVIVTVTIPNEEDGTYEVPFYAIDNYHYSRMENAVVLGATIGACSMLLIMLIGILFKNFQRL<br>RKSLLFNINFAILLMLILRSACYINYLMNNLSSISFFFTGIFDDESFMSSDAANAFKVILVALIEVSLTYQIYVM<br>FKTPMLKSWGIFASVLAGVLGLATLATQIYTTVMSHVNFVNGTTGSPSQVTSAWMDMPTILFSVSINVLS<br>MFLVCKLGLAIRTRRYLGLKQFDAFHILFIMSTQTMIIPSIILFVHYFDQNDSQTTLVNISLLLVVISLPLSSLW<br>AQTANNVRRIDTSPSMSFISREASNRSGNETLHSGATISKYNTSNTVNTTPGTSKDDSLFILDRSIPEQRIV<br>DTGLPKDLEKFINNDFYEDDGGMIAREVTMLKTAHNNQ
Cau	MEFTGDIVLKYTLGGEEYLSTFEQLDSSVNRSLELGVVHGIAIACGVLLMVLAWVIIIKKKNPIFVLNQLTLL<br>LMVIKSSLYLAFLFGPLSSLTYKFTRVLPHDKWHAFHVYIATNVIHTLLIATVEMTLVFQIYIIFKSPEVRHLG<br>YILTGAASALALTIVALYIHSTVISAVQLKEQLLMHEIKITNSWVNNVPIILFSASLNVVCIILIAKLALAIKTRRY<br>LGLKQFDGLHILMITSTQTFIVPSVLMIVNYKQSSSYLTLLANISVILVVCNLPLSSLWAASANNSSTPTSSA<br>NTVFSRWDSKFSDTETIAHELPLIPGKAEKLQLVSPITEKGDTHTMCESHGDQDLIDKMLDDIEGAVMTTE<br>FN LNNRTV
Yl	MQLPPRPDFDIATLVASITVPETELVLGQMPLGALEQLYQNRLRLAILFGVRVGAAVLTLIAMHLISKKNRT<br>KILFLANQMSLIMLIIHAALYFRFLLGPFASMLMMVAYIVDPRSNVSNDISVSVATNVFMMLMIMSVQLSLA<br>VQTRSVFHAWLKSRIYVTVGLILLSLVVFVFWTTHTIVSCIVLTHPTRDLPSMGWTRLASDVSFACSISFAS<br>LVLLAKLVTAIRVRKTLGKKPLGYTKVLVIMSTQSLVVPSILIIVNYALPEKNSWILSGVAYLMVVLSLPLSSI<br>WATAVHDDEMQSNYLLSALKDGHVQPSESKLKTVFLNRLRPFSTTTNRDDESSVDSPAMPSPESDVTFL<br>NTGFECDEKM
Cl	MNPADINIEYTLGDTAFSSTFADFEAWKTRNTQFAIVNGVALACGIILMVVSWIIIVNKRAPIFAMNQTMLVI<br>MVIKSAMYLKHIMGPLNSLTFRFTGLMEESWAPYNVYVTINVLHVLLVAAVESSLVFQIHVVFKSSRARVA<br>GRAIVSAMSTLALLIVSLYLYSTVRHAQTLRAELSHGDTTTVEPWVDNVPLILFSASLNVLCLLLALKLVFA<br>VRTRRHLGLRQFDSFHILIIMATQTFVIPSSLVIANYRYASSPLLSSISIIVAVCNLPLCSLWACSNNNSSYPT<br>SSQNTILSRYETETSQATDASSTTCAGIAEKGFDKSPDSPTFGDQDSVSISHILDSLEKDVEGVTTHRLT

Ca	MNINSTFIPDKPGDIIISYSIPGLDQPIQIPFHSLDSFQTDQAKIALVMGITIGSCSMTLIFLISIMYKTNKLTNL<br>KLKLKLKYILQWINQKIFTKKRNDNKQQQQQQQQQIESSSYNNTTTTTSGSYKLFLFYLNSLILLIGIIRSGC<br>YLNYNLGPLNSLSFVFTGWYDGSSFISSDVTNGFKCILYALVEISLGFQVYVMFKTSNLKIWGIMASLLSIG<br>LGLIVVAFQINLTILSHIRFSRAISTNRSEEESSSSLSSDSVGYVINSIWMDLPTILFSISINIMTILLIGKLIIAIR<br>TRRYLGLKQFDSFHILLIGFSQTLIIPSIILVVHYFYLSQNKDSLLQQISLLLIILMLPLSSLWAQTANNTHNIN<br>SSPSLSFISRHHSSDSSRSGGSNTIVSNGGSNGGGGGGGNFPVSGIDAQLPPDIEKILHEDNNYKLLNSN<br>NESVNDGDIIINDEGMITKQITIKRV
Ct	MDINNTIQSSGDIIITYTIPGIEEPFELPFEVLNHFQSEQSKNCLVMGVMIGSCSVLLIFLVGILFKTNKFSTIG<br>KSKNLSKNFLFYLNCLITFIGIIRAACFSNYLLGPLNSASFAFTGWYNGESYASSEAANGFRVILFALIETSM<br>VFQVFVMFRGAGMKKLAYSVTILCTALALVVVGFQINSAVLSHRRFVNTVNEIGDTGLSSIWLDLPTILFSV<br>SVNLMSVLLIGKLIMAIKTRRYLGLKQFDSFHVLLICSTQTLLVPSLILFVHYFLFFRNANVMLINISILLIVLML<br>PFSSLWAQTANTTQYINSSPSFSFISREPSANSTLHSSSGHYSEKSYGINKLNTQGSSPATLKDDHNSVIL<br>EATNPMSGFDAQLPPDIARFLQDDIRIEPSSTQDFVSTEVTYKKV
Cn	MDSYLLNHPGDISLNFALPLSDEVYTITFNDLDSQSSFSIQYLVIHSCAITVCLTLLVLLNLFIRNKKTPVFVL<br>NQVILFFAIVRSSLFIGFMKSPLSTITASFTGIISDDQKHFYKVSVAANAALIILVMLIQVSFTYQIYIIFRSPEV<br>RKFGVFMTSALGVLMAVTFGFYVNSAVASTKQYQHIFYSTDPYIMDSWVTGLPPILYSASVIAMSLVLVLK<br>LVAAVRTRRYLGLKQFSSYHILLIMFTQTLFVPTILTILAYAFYGYNDILIHISTTITVVLLPFTSIWASIANNSR<br>SLMSAASLYFSGSNSSLSELSSPSPSDNDTLNENVFAFFPDKLQKMNSSEAVSAVDKVVVHDHFDTISQ<br>KSIPHDILEILQGNEGGQMKEHISVYSDDSFSKTTPPIVGGNLLITNTDIGMK
Le	MDEAINANLVSGDIIVSFNIPGLPEPVQVPFSEFDSFHKDQLIGVIILGVTIGACSLLLILLLGMLYKSREKYW<br>KSLLFMLNVCILAATILRSGCFLDYYLSDLASISYTFTGVYNGTSFASSDAANVFKTIMFALIETSLTFQVYV<br>MFQGTTWKNWGHAVTALSGLLSVASVAFQIYTTILSHNNFNATISGTGTLTSGVWMDLPTLLFAASINFM<br>TILLLFKLGMAIRQRRYLGLKQFDGFHILFIMFTQTLFIPSILLVIHYFYQAMSGPFIINMALFLVVAFLPLSSL<br>WAQTANTTKKIESSPSMSFITRRKSEDESPLAANDEDRLRKFTTTLDLSGNKNNTTNNNNNSNNINNNM<br>SNINYPSTGLGEDDKSFIFEMEPSRERAAIEEIDLGARIDTGLPRDLEKFLVDGFDDSDDGEGMIAREVTM<br>LKK
Gc	MAEDSIFPNNSTSPLTNPIVVETIKGTAYIPLHYLDDLQYEKMLLASLFSVRIATSFVVIIWYFVAVNKAKRS<br>KFLYIVNQVSLLIVFIQSILSLIYVFSNFSKMSTILTGDYTGITKRDINVSCVASVFQFLFIACIELALFIQATVV<br>FQKSVRWLKFSVSLIQGSVALTTTALYMAIIVQSIYATLNPYAGNLIKGRFGYLLASLGKIFFSISVTSCMCIF<br>VGKLVFAIHQRRTLGIKQFDGLQILVIMSTQSMIIPTIIVLMSFLRRNAGSVYTMATLLVALSLPLSSLWAEA<br>KTTRDSASYTAYRPSGSPNNRSLFAIFSDRLACGSGRNNRHDDDSRGNGSVNARKADVESTIEMSSCY<br>TDSPTYSKFEAGLDARGIVFYNEHGLPVVSGEVGGSSSNGTKLGSGHKYEVNTTVVLSDVDSPSPTDVT<br>RK
Bm	MASNGWQNNATFDPYAQTFVLLQPDGLTPFPALLGDVLALNTVSVTQGIIYGTQVGISGLLLLILLIMTKPD<br>KRRSLVFILNSLSLLLIFARNVLSCVQLTTIFYNFYNWELHWYPESPALSRAMDLSAATEVLNIPIDVAIFSS<br>LVVQVHIVCCTIHTLVRTSALLSSAAVGLAAVAVRFALAVVNIKYSIFGINTLTEPQFNLIVHLKRVSDILTVV<br>AIAFFSSIFVAKLGVAIHTRRTLNLKNFGAIQIIFIMGCQTMLIPLIFVIVSFYASRGSQIGSMVPTVVATFLPL<br>SGMWASAQTNNEKMGRADQRFHRAVPVGATDFSVTKARSAKASDTLDTLIGDD
So	MREPWWKNYYTMNGTQVQNQSIPILSTQGYIQVPLSTIDKAERNRILTGMTVSAQLALGVLIMVMSILLSS<br>PEKRKTPVFIVNSASIISMCIRAILMIVNLCSESYSLAVMYGFVFELVGQYVHVFDILVMIIGTIIIITAEVSMLL<br>QVRIICAHDRKTQRIVTCISSGLSLIVVAFWFTDMCQEIKYLLWLTPYNNHQISGYYWVYFVGKILFAVSIM<br>FHSAVFSYKLFHAIQIRKKIGQFPFGPMQCILIISCQCLFVPAIFTIIDSFIHTYDGFSSMTQCLLIVSLPLSSL<br>WASSTALKLQSLKSTTSPGDTTQVSIRVDRTYDIKRIPTEELSSVDETEIKKWP
Tm	MEQIPVYERPGFNPHKQNITLFKHDGSTVTVGLHELDAMFTHSIRVAVVFASQIGACALLSVIVAMVTKRE<br>KRRALFFLHIISLLLVVVRSVLQILYFVGPWAETYNYVAYYYEDIPLSDKLISIWAGIIQLILNICILLSLILQVRV<br>VYATSPKLNTIMTLVSCVIASISVGFFFTVIVQISEAILNGVGYDGWVYKVHRGVFAGAIAFFSFIFIFKLAFAI<br>RRRKALGLQRFGPLQVIFIMGCQTMIVPAIFATLENGVGFEGMSSLTATLAVISLPLSSMWAAAQTDGPS<br>PQSTPRDGYRRFSTRRSALNRSDPSGGRSVDMNTLDSTGNDSLALHVDKTFTVESSPSSQSQAGPHKE<br>RGFEFA
Ao	MDSKFDPYSQNLTFHAADGTPFQVPVMTLNDFYQYCIQICINYGAQFGASVIIFIILLLLTRPDKRASSVFFL<br>NGGALLLNMGRLLCHMIYFTTDFVKAYQYFSSDYSRAPTSAYANSILGVVLTTLLLVCIETSLVLQVQVVC<br>ANLRRRYRTVLLCVSILVALIPVGLRLGYMVENCKTIVQTDTPLSLVWLESATNIVITISICFFCSIFIIKLGFAI<br>HQRRRLGVRDFGPMKVIFVMGCQTLTVPALLSILQYAVSVPELNSNIMTLVTISLPLSSIWAGVSLTRSSS<br>TENSPSRGALWNRLTDSTGTRSNQTSSTDTAVAMTYPSNKSSTVCYADQSSVKRQYDPEQGHGISVEH<br>DVSVHSCQRL
Sp	MRQPWWKDFTIPDASAIIHQNITIVSIVGEIEVPVSTIDAYERDRLLTGMTLSAQLALGVLTILMVCLLSSSE<br>KRKHPVFVFNSASIVAMCLRAILNIVTICSNSYSILVNYGFILNMVHMYVHVFNILILLLAPVIIFTAEMSMMIQ<br>VRIICAHDRKTQRIMTVISACLTVLVLAFWITNMCQQIQYLLWLTPLSSKTIVGYSWPYFIAKILFAFSIIFHS<br>GVFSYKLFRAILIRKKIGQFPFGPMQCILVISCQCLIVPATFTIIDSFIHTYDGFSSMTQCLLIISLPLSSLWAS<br>STALKLQSMKTSSAQGETTEVSIRVDRTFDIKHTPSDDYSISDESETKKWT

Af	MNSTFDPWTQNITLTQSDGTTVISSLALADDYLHYMIRLGINYGAQLGACAVLLLVLLLLTRPEKRVSSVF<br>VLNVAALLANIIRLGCQLSYFSTGFARMYALLAGDFSRVSRGAYAGQVMASVFFTIVFICVEASLVLQVQV<br>VCSNLRRQYRILLLGASTLAALVPIGVRLTYSVLNCMVIMHAGTMDHLDWLESATNIVTTVSICFFCAVFVV<br>KLGLAIKMRKRLGVKQFGPMRVIFIMGCQTMTIPAIFAICQYFSRIPEFSHNVLTLVIISLPLSSIWAGFALVQ<br>ANSTARSTESRHHLWNILSSDGATRDKPSQCVSSPMTSPTTTCYSEQSTSKPQQDPENGFGISVAHDISI<br>HSFRKDAHGDI
Pd	MSTANVHLPADFDPTRQNITIYTPDGTPVVATLPMINLFNRQNNEICVVYGCQLGASLIMFLVVLLTTRVS<br>KRKSPIFVLNVLSLIISCLRSLLQILYYIGPWTEIYRYLSFDYSTVPASAYANSVAATLLTLFLLITIEASLVLQT<br>NVVCKSMSSHIRWPVTALSMVVSLLAISFRFGLTIRNIEGILGATVKSDSLMFSGASLISETASIWFFCTIFVI<br>KLGWTLYQRKKMGLKQWGPMQIITIMAGCTMLIPSLFTVLEFFPEETFYEAGTLAICLVAILLPLSSVWAAA<br>AIDGDEPVRPHGSTPKFASFNMGSDYKSSSAHLPRSIRKASVPAEHLSRTSEEELGDDGTLNRGGAYG<br>MDRMSGSISPRGVRIERTYEVHTAGRGGSIEREDIF
Sj	MYSWDEFRSPKQAEVLNQTVTLETIVSTIQLPISEIDSMERNRLLTGMTVAVQVGLGSFILVLMCIFSSSEK<br>RKKPVFIFNFAGNLVMTLRAIFEVIVLASNNYSIAVQYGFAFAAVRQYVHAFNIIILLLGPFILFIAEMSLMLQ<br>VRIICSQHRPTMITTTVISCIFTVVTLAFWITDMSQEIAYQLFLKNYNMKQIVGYSWLYFIAKITFAASIIFHSS<br>VFSFKLMRAIYIRRKIGQFPFGPMQCIFIVSCQCLIVPAIFTLIDSFTHTYDGFSSMTQCLLIISLPLSSLWAT<br>HTAQKLQTMKDNTNPPSGTQLTIRVDRTFDMKFVSDSSDGSFTEKTEETLP
Pb	MAPSFDPFNQNVVFHKADGTPFNVSIHELDDFVQYNTRVCINYSSQLGASVIAGLMLAMLTHSEKRRLPV<br>FFLNTFALAMNFARLLCMTIYFTTGFNKSYAYFGQDYSQVPGSAYAASVLGVVFTTLLVISMEMSLLIQTR<br>VVCTTLPDIQRYLLMAVSSAISLMAIGFRLGLMVENCIAIVQASNFAPFIWLQSASNITITISTCFFSAVFVTK<br>LAYALVTRIRLGLTRFGAMQVMFIMSCQTMVIPAIFSILQYPLPKYEMNSNLFTLVAIFLPLSSLWASVATK<br>SSFETSSSGRHQYLWPSEQSNNVTNSEIKYQVSFSQNHTTLRSGGSVATTLSPDRLDPVYSEVEAGTKA
Mg	MVVTAPPSVDRTYFIPNSTFDPYQQDLTLVYPDGVHALVANVDDIVYFMGLAVKSTLIFAIQIGISFVLMLVI<br>ALLTKPERRVTLVFFLNMTALFTIFIRAILMCTTFVGTYYNFYNWIMGNYPNSGLADRVSIAAEVFAFLIILSL<br>ELSMMFQVRIVCINLSSFRRRIITFSSIVVAMIVCTVRFALMVLSCDWRIVNIGDATQEKNRIINRVASGYNI<br>CTIASIIFFNTIFVSKLAVAIKHRRSMGMKQFGPMQIIFVMGCQTLLIPAIFGIISYFALASTQVYSLMPMVVAI<br>FLPLSSMWASFNTNKTNSVTNMRQPNVYRPNMIIGQDTTQNSGKNTNISGTSNSTATTSSFASDKRRLN<br>LSFNTQGTLVNSISEEEVNNPQKLGPSATVAVMDRDSLELEMRQHGIAQGRSYSVRSD
Pr	MATSSPIQPFDPFTQNVTFRLQDGTEFPVSVKALDVFVMYNVRVCINYGCQFGASFVLLVILVLLTQSDK<br>RRSAVFILNGLALFLNSSRLLFQVIHFSTAFEQVYPYVSGDYSSVPWSAYAISIVAVVLTTLVVVCIEASLVI<br>QVHVVCSTLRRRYRHPLLAISILVALVPIGFRCAWMVANCKAIIKLTYTNDVWWIESATNICVTISICFFCVIF<br>VTKLGFAIKQRRRLGVREFGPMKVIFVMGCQTMVVPAIFSITQYYVVVPEFSSNVVTLVVISLPLSSIWAG<br>AVLENARRTGSQDRQRRRNLWRALVGGAESLLSPTKDSPTSLSAMTAAQTLCYSDHTMSKGSPTSRDT<br>DAFYGISVEHDISINRVQRNNSIV
An	MATHNQISDQCQWSYPEVFTTQAVEEPTAEPASYHLHSTLTIMASNFDPWNQTITFRLEDGTPFDISVDY<br>LDGILQYSIRACVNYAAQLGASVILFVILVLLTRAEKRASCLFWLNSLALLLNFARLLCDVLFFTGNFVRIYT<br>LISADESRVTASDLATSIVGAIMTALLLTTIEISLVLQVQVVCSNLRRIYRRALLCVSAVVATATIAIRYSLLAV<br>NIRAILEFSDPTTYNWLESLATVALTISICYFCVIFVTKLGFAIRLRRKLGLSELGPMKVVFIMGCQTLVIPGK<br>RTLSSLIPPVIVSITHYVSDVPELQTNVLTIVALSLPLSSIWAGTTIDKPVTHSNVRNLWQILSFSGYRPKQS<br>TYIATTTTATTNAKQCTHCYSESRLLTEKESGRNNDTSSKSSSQYGIAVEHDISVRSARRESFDV
Sn	MASMVPPPDFDPYTQEFMVLGPDGQEIPISMQTVNEYRLYTARLGLAYGSQIGATLLLLLVLSLLTTRREK<br>RKSGIFIVNALCLVTNTIRCILLSCFVTSTLWHPYTQFSQDTSRVSKTDVNTSIAASIFTLIVTVLIMISLSVQV<br>WVVCITTAPYQRYMIMGATTATAMVAVGYKAAFVITSIIQTLNGQDGGSYLDLVMQSYITQAVAISFYSCIF<br>TYKLGHAIVQRRTLNMPQFGPMQIIFIMGSLFTGLQFVKNVDELGIITPTIVCIFLPLSAIWAGVVNEKVVGA<br>NGPDAHHRLLQGEFYRAASNSTYGSNSSGTVVDRSRQMSVCTCASSSPFVRKKSVAEWDDEAILVGRE<br>FGFSRGEVGERG
Hj	MSSFDPYTQNITILVSPSSPPISIPIPVIDAFNDETASIITNYAAQLGAALAMLLVLLAATPTARLLRADGPSLL<br>HALALLVCVVRTVLLIYFFLTPFSHFYQVWTGDFSQVPAWNYRASIAGTVLSTLLTVVTDAALVNQAWTM<br>VSLFAPRTKRAVCVLSLLITLLAISFRVAYTVIQCEGIAELAAPRQYAWLIRATLIFNICSIAWFCALFNSKLV<br>AHLVTNRGVLPSRRAMSPMEVLIMANGILMIVPVVFAILEWHHFINFEAGSLTPTSIAIILPLSSLAAQRIANT<br>SSS
Bc	MASNSSNFDPLTQSITILMADGITTVSFTPLDIDFFYYYNVACCINYGAQAGACLLMFFVVVVLTKAVKRKT<br>LLFVLNVLSLIFGFLRAMLYAIYFLQGFNDFYAAFTFDFSRVPRSSYASSVAGSVIPLCMTITVNMSLYLQA<br>YTVCKNLDDIKRIILTTLSAIVALLAIGFRFAATVVNSVAILATSASSVPMQWLVKGTLVTETISIWFFSLIFTG<br>KLVWTLYNRRRNGWRQWSAVRILAAMGGCTMVIPSIFAILEYVTPVSFPEAGSIALTSVALLLPISSLWAG<br>MVTDEETSAIDVSNLTGSRTMLGSQSGNFSRKTHASDITAQSSHLDFSSRKGSNATMMRKGSNAMDQV<br>TTIDCVVEDNQANRGLRDSTEMDLEAMGVRVNKSYGVQKA

Bb	MDGSSAPSSPTPDPTFDRFAGNVTFFLADHITTTSVPMPVLNAYYDESLCTTMNYGAQLGACLVMLVVV VALTPAAKLARRPASALHLVGLLLCAVRSGLLFAYFVSPISHFYQVWAGDFSAVSRRYWDASLAANTLAF PLVVVVEAALINQAWTMVAFWPRAAKAAACACSAVIVLLTIGTRLAYTIVQNHAIVTAVPPEHFLWAIQWS AVMGAVSIFWFCAVFNVKLVCHLVANRGILPSISVVNPMEVLVMTNGTLMIIPSIFAGLEWAKFTNFESGS LTLTSVIIILPLGTLAAQRISGQGSQGYQAGHLFHEQQQQQARTRSGAFGSASQQSHPTNKVPSSITLSTS GTPITPQISAGSRPELPLVDRSERLDPIDLELGRIDAFRGSSDFSPSTARPKRMQRDNFA
Nc	MASSSSPPADIFSGITQSLNSTHATLTLPIPPADRDHLENQVLFLFDNHGQLLNVTTTYIDAFNNMLVSTTI NYATQIGATFIMLAIMLLMTPRRRFKRLPTIISLLALCINLIRVVLLALFFPSHWTDFYVLYSGDWQFVPPGD MQISVAATVLSIPVTALLLSALMVQAWSMMQLWTPLWRALVVLVSGLLSLVTVAMSFANCIFQAKNILYAD PLPSYWVRKLYLALTTGSISWFTFLFMIRLVMHMWTNRSILPSMKGLKAMDVLIITNSILMLIPVLFAGLEFL DSASGFESGSLTQTSVVIVLPLGTLVAQRIATRGYMPDSLEASSGPNGSLPLSNLSFAGGGGGGSGGHK DKENGGGIIPPTTNNTAATNFSSSIACSGISCLPKVKRMTASSASSSQRPLLTMTNSTIASNDSSGFPSPG IHNTTTTTTQYQYSMGMNMPNFPPVPFPGYQSRTTGVTSHIVSDGRHHQGMNRHPSVDHFDRELARID DEDDDGYPFASSEKAVMHGDDDDDVERGRRRALPPSLGGVRVERTIETRSEERMPSPDPLGVTKPRSF E
She	MKPAAGPASSPFDPFNQTFYLTGPDNTTVPVSVPQVDYIWHYIIGTSINYGSQIGACLLMLLVMLTLTSKS RFSRAATLINVASLLIGVIRCVLLAVYFTSSLTELYALFVGDYSQVRRSDLCVSAVATFFSLPQLVLIEAALF LQAYSMIKMWPSLWRAVVLAMSVVVAVCAIGFKFASVVMRMRSTLTLDDSLDFWLVEVDLAFTATTIFW FCFIYIIRLVIHMWEYRSILPPMGSVSAMEVLVMTNGALMLVPVIFAAIEINGLSSFESGSLVHTSVIVLLPLG SLIAQAMTRPDGYVQRTNTSGASGASGAHPGRNGSGHGGHGGAYSRAMTNTLNTLDTLDTVDSKTSIM HHHHHHHRNHSNGMSKTKANSGTWSHASDANSTNAMISGGIATQVRIQANQSTLGNTGMSGGSGAPN SHTRNNSLAAMEPVEKQLHDIDATPLSASDCRVWVDREVEVRRDMV
Mo	MDQTLSATGTATSPPGPALTVDPRFQTITMLTPALMGQGFEEVQTTPAEINDVYFLAFNTAIGYSTQIGAC FIMLLVLLTMTAKARFARIPTIINTAALVVSIIRCTLLVIFFTSTMMEFYTIFSDDFSFVHPNDIRRSVAATVFA PLQLALVEAALMVQAWAMVELWPRAWKVSGIAFSLILATVTVAFKCASAAVTVKSALEPLDPRPYLWIRQ TDLAFTTAMVTWFCFLFNVRLIMHMWQNRSILPTVKGLSPMEVLVMANGLLMVFPVLFAGLYYGNFGQF ESASLTITSVVLVLPLGTLVAQRLAVNNTVAGSSANTDMDDKLAFLGNATTVTSSAAGFAGSSASATRSR LASPRQNSQLSTSVSAGKPRADPIDLELQRIDDEDDDFSRSGSAGGVRVERSIERREERL
Dh	MDHNTQHFNRPEYIEIPVPPSKGFNPHTNPAFFIYPDGSNMTFWFGQIDDFRRDQLFTNTIFSIQIGAALVI LCVMFCVTHADKRKTIVYLLNVSNLFVVIIRGVFFVHYFMGGLARTYTTFTWDTSDVQQSEKATSIVSSIC SLILMIGTQISLLLQVRICYALNPRSKTAILVTCGSISGIATTAYLLLGAYTIQLREKPPDMKFMKWAKPVVN ALVALSIVSFSGIFSWRMFQSVRNRRRMGFTGIGSLESLLASGFQCLVFPGLVTTALTVAGSTWYIAVNLT TPSDLTAIYNCSAFFAYAFSIPLLKERAQVEKTISVVIAIAGVLVVAYGDGADDGSTSNGEKARLGGNVLIGI GSVLYGLYEVLYKKLLCPPSGASPGRSVVFSNTVCACIGAFTLLFLWIPLPLLHWSGWEIFELPTGKTAKL LGISIAANATFSGSFLILISLTGPVLSSVAALLTIFLVAITDRILFGRELTSAAILGGLLIIAAFALLSWATWKEMI EENEKDTIDSISDVGDHDD
Fg	MSKEAFDPFTQNVTFFAPDGKTEINIPVAAIDQVRRMMVNTTINYATQLGACLIMLVVILVMVPKEKFRRP FMILQIASLVICCCRMLLLSIFHSSQFLDFYVFWGDDHSRIPRSAYAPSVAGNTMSLCLVISVETMLMSQA WTMVRLWPNVWKYIIAGISLVVSIVAISVRLAYTIIQNNAVLKLEPAFHMFWLIKWTVIMNVASISWWCAIF NIKLVWHLISNRGILPSYKTFTPMEVLIMTNGILMIIPVIFASLEWAHFVDFESASLTLTSVAVILPLGTLAAQ RIASSAPNSANSTGASSGIRYGVSGPSSFTGFKAPSFSTGTTDRPHVSIYARCEAGTSSREHINPQDVEL AKLDPETDHHVRVDRAFLQREERIRAPL
Cc	MAARIIPALTLTAPTSYPTAGVGGYYYDTAFGVPTYSSAAFNQTTWRLLDNWDHINVNYASSEGLAAGLG WATLIYLLALTPSHKRTTPFHCFLLVGLIFLLGHLMVNIIAALTPGLNTTSAYTYVTLDTSSSVWPRKYIAVY AVNAVASWFAFIFATICLWLQAKGLMTGIRVRFIIVYKIILMYLIVAAVIALAICMAFNIQQILYIGKPVELADGT ALLRLRNAYLITYAISIGSFSLVSICSIMDIIWRRPSRVIKGHNIFASALNLVGLLCAQSFVVPCEYKRALGQV PDCTTFADHIFHTVIFCILQVIPNSSGVMLPEIMLLPSVYVILPLGSLFMTVNSPESDVNKTSFPPKSSPGPF DRSPTLTSGTLPGSRPESYVLDMASDKNSGNRKSVCSQFDRELNLIDSLDTLSGREGDSMLHAQSNNN NQTREQDKQPRADTTHVGSENMV

Supplementary Table 9 – Primers used in this study

Primer	Primer Sequence 5'→3'	Application
BAR1_delta_C	GATATTTATATGCTATAAAGAAATTGTACTCCAGATTTCccaTATATGACCCTTCTAGAC	CRISPR deletion of BAR1 gene and verification
BAR1_delta_W	TCATACCAAAATAAAAAGAGTGTCTAGAAGGGTCATATAtggGAAATCTGGAGTACAATT	
BAR1_FWD	GGCTGCACTCATTCCGGTAC	
BAR1_RVS	ACGGACGTTTAGGATGACGTATTG	
BAR1.3_C	GCTATTTCTAGCTCTAAAACatatttagtttcatgtacaaCTGCCAATCGCAGCTCCCAG	
BAR1.3_W	CTGGGAGCTGCGATTGGCAGttgtacatgaaactaaatatGTTTTAGAGCTAGAAATAGC	
BAR1.5_C	GCTATTTCTAGCTCTAAAACaaataagtttcaaacaaagaGATCATTTATCTTTCACTGC	
BAR1.5_W	GCAGTGAAAGATAAATGATCtctttgtttgaaacttatttGTTTTAGAGCTAGAAATAGC	
FAR1_delta_C	AGCAAAAGCCTCGAAATACGGGCCTCGATTCCCGAACTAccaTAATAGATTGCCTTCTTA	CRISPR deletion of FAR1 gene and verification
FAR1_delta_W	CCACTGGAAAGCTTCGTGGGCGTAAGAAGGCAATCTATTAtggTAGTTCGGGAATCGAGG	
FAR1_FWD	GTTAGGCGGGCAAGAGAGAC	
FAR1_RVS	CGGAACAAATTAGCCACATCGACG	
FAR1.3_C	GCTATTTCTAGCTCTAAAACgggtctgatgaattctttgcCTGCCAATCGCAGCTCCCAG	
FAR1.3_W	CTGGGAGCTGCGATTGGCAGgcaaagaattcatcagacccGTTTTAGAGCTAGAAATAGC	
FAR1.5_C	GCTATTTCTAGCTCTAAAACcttggtggagtgtgtattttGATCATTTATCTTTCACTGC	
FAR1.5_W	GCAGTGAAAGATAAATGATCaaaatacacactccaccaagGTTTTAGAGCTAGAAATAGC	
MF_Bb_C	AAAAGGGGCCTGTctcaCTAccaacatggttgacctggtctcatacaccaAGCTTCAGCCTCTCTTTTAT	Homology primers for construction of Peptide expression vectors via Gibson Assembly
MF_Bb_W	ATAAAAGAGAGGCTGAAGCTtggtgtatgagaccaggtcaaccatgttggTAGtgagACAGGCCCCTTTT	
MF_Bc_C	AAAAGGGGCCTGTCTCACTAacatggttgacctggtctaccacaccaAGCTTCAGCCTCTCTTTTAT	
MF_Bc_W	ATAAAAGAGAGGCTGAAGCTtggtgtggtagaccaggtcaaccatgtTAGTGAGACAGGCCCCTTTT	
MF_Ca_C	AAAAGGGGCCTGTCTCACTAacctggttcgaagtaaccgaagttggtcaatctgaaaccAGCTTCAGCCTCTCTTTTAT	
MF_Ca_W	ATAAAAGAGAGGCTGAAGCTggtttcagattgaccaacttcggttacttcgaaccaggtTAGTGAGACAGGCCCCTTTT	
MF_Cl_C	AAAAGGGGCCTGTCTCACTAaccgataacgtcggtgtttctgaacttgatccacttccacttAGCTTCAGCCTCTCTTTTAT	
MF_Cl_W	ATAAAAGAGAGGCTGAAGCTaagtggaagtggatcaagttcagaaacaccgacgttatcggtTAGTGAGACAGGCCCCTTTT	
MF_EAEA_Bb_C	AGGAAAAGGGGCCTGTcTCAccaacatggttgacctggtctcatacaccaTCTTTTATCCAAAGATACCC	
MF_EAEA_Bb_W	GGGTATCTTTGGATAAAAGAtggtgtatgagaccaggtcaaccatgttggTGAgACAGGCCCCTTTTCCT	

MF_EAEA_Cl_C	AGGAAAAGGGGCCTGTcTCAaccgataacgtcggtgtttctgaacttgatccacttccactt TCTTTTATCCAAAGATACCC	
MF_EAEA_Cl_W	GGGTATCTTTGGATAAAAGAaagtggaagtggatcaagttcagaaacaccgacgttatc ggtTGAgACAGGCCCCTTTTCCT	
MF_EAEA_Hj_C	AGGAAAAGGGGCCTGTcTCAccaacatggttcaccgattctgtaacaccaTCTTTTA TCCAAAGATACCC	
MF_EAEA_Hj_W	GGGTATCTTTGGATAAAAGAtggtgttacagaatcggtgaaccatgttggTGAgACA GGCCCCTTTTCCT	
MF_EAEA_Kp_C	AGGAAAAGGGGCCTGTcTCAaccgaatggttggttcttttcgttgtttctccatctgaaTCTT TTATCCAAAGATACCC	
MF_EAEA_Kp_W	GGGTATCTTTGGATAAAAGAttcagatggagaaacaacgaaaagaaccaaccattcgg tTGAgACAGGCCCCTTTTCCT	
MF_EAEA_Le_C	AAAAGGGGCCTGTCTCACTaaactggagagaatctaccgtatctggtccacatccaAG CTTCAGCCTCTCTTTTAT	
MF_EAEA_Le_Cnew	AGGAAAAGGGGCCTGTcTCAaactggagagaatctaccgtatctggtccacatccaTC TTTTATCCAAAGATACCC	
MF_EAEA_Le_W	ATAAAAGAGAGGCTGAAGCTtggatgtggaccagatacggtagattctctccagtttAGT GAGACAGGCCCCTTTT	
MF_EAEA_Le_Wnew	GGGTATCTTTGGATAAAAGAtggatgtggaccagatacggtagattctctccagttTGAg ACAGGCCCCTTTTCCT	
MF_EAEA_Pd_C	AGGAAAAGGGGCCTGTcTCAaccacatggttgacctggtctccaacagaaTCTTTTA TCCAAAGATACCC	
MF_EAEA_Pd_W	GGGTATCTTTGGATAAAAGAttctgttggagaccaggtcaaccatgtggtTGAgACAG GCCCCTTTTCCT	
MF_EAEA_Zr_C	AAAAGGGGCCTGTCTCACTAgaacattggttgacctgggtccaattcgatgaagtgAGC TTCAGCCTCTCTTTTAT	
MF_EAEA_Zr_Cnew	AGGAAAAGGGGCCTGTcTCAgaacattggttgacctgggtccaattcgatgaagtgTCT TTTATCCAAAGATACCC	
MF_EAEA_Zr_W	ATAAAAGAGAGGCTGAAGCTcacttcatcgaattggacccaggtcaaccaatgttcTAG TGAGACAGGCCCCTTTT	
MF_EAEA_Zr_Wnew	GGGTATCTTTGGATAAAAGAcacttcatcgaattggacccaggtcaaccaatgttcTGA gACAGGCCCCTTTTCCT	
MF_Hj_C	AAAAGGGGCCTGTctcaCTAccaacatggttcaccgattctgtaacaccaAGCTTCAG CCTCTCTTTTAT	
MF_Hj_W	ATAAAAGAGAGGCTGAAGCTtggtgttacagaatcggtgaaccatgttggTAGtgagA CAGGCCCCTTTT	
MF_Kp_C	AAAAGGGGCCTGTctcaCTAaccgaatggttggttcttttcgttgtttctccatctgaaAGCTT CAGCCTCTCTTTTAT	
MF_Kp_W	ATAAAAGAGAGGCTGAAGCTttcagatggagaaacaacgaaaagaaccaaccattcg gtTAGtgagACAGGCCCCTTTT	
MF_Le_C	AAAAGGGGCCTGTCTCACTAaactggagagaatctaccgtatctggtccacatccaAG CTTCAGCCTCTCTTTTAT	
MF_Le_W	ATAAAAGAGAGGCTGAAGCTtggatgtggaccagatacggtagattctctccagttTAG TGAGACAGGCCCCTTTT	
MF_Pb_C	AAAAGGGGCCTGTCTCACTAacaaccttgacctggtctggtacaccaAGCTTCAG CCTCTCTTTTAT	
MF_Pb_W	ATAAAAGAGAGGCTGAAGCTtggtgtaccagaccaggtcaaggttgtTAGTGAGAC AGGCCCCTTTT	
MF_Pd_C	AAAAGGGGCCTGTctcaCTAaccacatggttgacctggtctccaacagaaAGCTTCA GCCTCTCTTTTAT	
MF_Pd_W	ATAAAAGAGAGGCTGAAGCTttctgttggagaccaggtcaaccatgtggtTAGtgagA CAGGCCCCTTTT	

MF_Sc_C	AAAAGGGGCCTGTCTCACTAgtacattggttgacctggcttcaattgcaaccagtgccaAGCTTCAGCCTCTCTTTTAT	
MF_Sc_W	ATAAAAGAGAGGCTGAAGCTtggcactggttgcaattgaagccaggtcaaccaatgtacTAGTGAGACAGGCCCCTTTT	
MF_Vp_C	AAAAGGGGCCTGTCTCACTAgtagattggttgaccgttgtccaattccaaccagtgccaAGCTTCAGCCTCTCTTTTAT	
MF_Vp_W	ATAAAAGAGAGGCTGAAGCTtggcactggttggaattggacaacggtcaaccaatctacTAGTGAGACAGGCCCCTTTT	
MF_Zr_C	AAAAGGGGCCTGTCTCACTAgaacattggttgacctgggtccaattcgatgaagtgAGCTTCAGCCTCTCTTTTAT	
MF_Zr_W	ATAAAAGAGAGGCTGAAGCTcacttcatcgaattggacccaggtcaaccaatgttcTAGTGAGACAGGCCCCTTTT	
MF-EAEA_Bc_C	AGGAAAAGGGGCCTGTCTCAacatggttgacctggtctaccacaccaTCTTTTATCCAAAGATACCC	
MF-EAEA_Bc_W	GGGTATCTTTGGATAAAAGAtggtgtggtagaccaggtcaaccatgtTGAGACAGGCCCCTTTTCCT	
MF-EAEA_Ca_C	AGGAAAAGGGGCCTGTCTCAacctggttcgaagtaaccgaagttggtcaatctgaaaccTCTTTTATCCAAAGATACCC	
MF-EAEA_Ca_W	GGGTATCTTTGGATAAAAGAggtttcagattgaccaacttcggttacttcgaaccaggtTGAGACAGGCCCCTTTTCCT	
MF-EAEA_Pb_C	AGGAAAAGGGGCCTGTCTCAacaaccttgacctggtctggtacaccaTCTTTTATCCAAAGATACCC	
MF-EAEA_Pb_W	GGGTATCTTTGGATAAAAGAtggtgtaccagaccaggtcaaggttgtTGAGACAGGCCCCTTTTCCT	
MF-EAEA_Sc_C	AGGAAAAGGGGCCTGTCTCAgtacattggttgacctggcttcaattgcaaccagtgccaTCTTTTATCCAAAGATACCC	
MF-EAEA_Sc_W	GGGTATCTTTGGATAAAAGAtggcactggttgcaattgaagccaggtcaaccaatgtacTGAGACAGGCCCCTTTTCCT	
MF-EAEA_Vp_C	AGGAAAAGGGGCCTGTCTCAgtagattggttgaccgttgtccaattccaaccagtgccaTCTTTTATCCAAAGATACCC	
MF-EAEA_Vp_W	GGGTATCTTTGGATAAAAGAtggcactggttggaattggacaacggtcaaccaatctacTGAGACAGGCCCCTTTTCCT	
MFa.5_C	GCTATTTCTAGCTCTAAAACgaagacacctttgataatatGATCATTTATCTTTCACTGC	CRISPR deletion of MFa1 gene and verification
MFa.5_W	GCAGTGAAAGATAAATGATCatattatcaaaggtgtcttcGTTTTAGAGCTAGAAATAGC	
MFa1_FWD	CTGCTACGGTTGGCCCATAC	
MFa1_RVS	ACTTCACGGTAGGTGGTAAGC	
MFa1.5_C	GCTATTTCTAGCTCTAAAACtcttttcactgctggtctttGATCATTTATCTTTCACTGC	
MFa1.5_W	GCAGTGAAAGATAAATGATCaaagaccagcagtgaaaagaGTTTTAGAGCTAGAAATAGC	
MFa1delta_C	AAGATAAAGGAGGGAGAACAACGTTTTTGTACGCAGAAATTCTATTCGATGGCTTTGTACTTATTTTGGTTTTATCCG	
MFa1delta_W	TCGGATAAAACCAAAATAAGTACAAAGCCATCGAATAGAATTTCTGCGTACAAAAACGTTGTTCTCCCTCCTTTATCT	
MFa2_FWD	TTCCATCCACTTCTTCTGTCGTTC	CRISPR deletion of MFa2 gene and verification
MFa2_RVS	GGGTGGTTCATCTTTCATTTCCTGC	
MFa2.3_C	GCTATTTCTAGCTCTAAAACtctgagtggcttgtgtggaaCTGCCAATCGCAGCTCCCAG	

MFa2.3_W	CTGGGAGCTGCGATTGGCAGttccacacaagccactcagaGTTTTAGAGCTAGAAATAGC	
MFa2.5_C	GCTATTTCTAGCTCTAAAACtctgagtggcttgtgtggaaGATCATTTATCTTTCACTGC	
MFa2.5_W	GCAGTGAAAGATAAATGATCttccacacaagccactcagaGTTTTAGAGCTAGAAATAGC	
MFa2delta_C	AGGGTAGATATTGATTTGACCTCTTGGTTGTCGTCAAAAATAAGGTTGGTAGTTATTGTTGTATGAAGATGATAGCTCG	
MFa2delta_W	GCGAGCTATCATCTTCATACAACAATAACTACCAACCTTATTTTTGACGACAACCAAGAGGTCAAATCAATATCTACC	
MFalpha1_FWD	TGCGCTAAATAGACATCCCGTTC	CRISPR deletion of MFalpha1 gene and verification
MFalpha1_RVS	CAGAGGCATCATAATCAGGGAGTG	
MFalpha1.3_C	gctatttctagctctaaaacggttttaactgcaaccaatgCTGCCAATCGCAGCTCCCAG	
MFalpha1.3_W	CTGGGAGCTGCGATTGGCAGcattggttgcagttaaaaccgttttagagctagaaatagc	
MFalpha1.5_C	GCTATTTCTAGCTCTAAAACTCAATTTTTACTGCAGTTTTGATCATTTATCTTTCACTGC	
MFalpha1.5_W	GCAGTGAAAGATAAATGATCAAAACTGCAGTAAAAATTGAGTTTTAGAGCTAGAAATAGC	
MFalpha1delta_C	GTCGACTTTGTTACATCTACACTGTTGTTATCAGTCGGGCTCTTTTAATCGTTTATATTGTGTATGAAATTGATAGTTT	
MFalpha1delta_W	CAAACTATCAATTTCATACACAATATAAACGATTAAAAGAGCCCGACTGATAACAACAGTGTAGATGTAACAAAGTCGA	
MFalpha2_FWD	GGCGACGCCTGTAGTGATTG	CRISPR deletion of MFalpha2 gene and verification
MFalpha2_RVS	GGGAACCTTGCTTGCAGACAG	
MFalpha2.3_C	gctatttctagctctaaaacGGCTTGAGTTGCAACCAGTGCTGCCAATCGCAGCTCCCAG	
MFalpha2.3_W	CTGGGAGCTGCGATTGGCAGCACTGGTTGCAACTCAAGCCgttttagagctagaaatagc	
MFalpha2.5_C	GCTATTTCTAGCTCTAAAACttctcacttttattttagcgGATCATTTATCTTTCACTGC	
MFalpha2.5_W	GCAGTGAAAGATAAATGATCcgctaaaataaaagtgagaaGTTTTAGAGCTAGAAATAGC	
MFalpha2delta_C	AAGAAATCGAGAGGGTTTAGAAGTAGTTTAGGGTCATTTTTTTCTCCAATATGTGAATTTACTGGAATTTGATGCAGGT	
MFalpha2delta_W	CACCTGCATCAAATTCCAGTAAATTCACATATTGGAGAAAAAAATGACCCTAAACTACTTCTAAACCCTCTCGATTTCT	
SST2_donor_C	GTGCAATTGTACCTGAAGATGAGTAAGACTCTCAATGAAAccaCTTACAAC	CRISPR deletion of SST2 gene and verification
SST2_donor_W	GTTATAGGTTCAATTTGGTAATTAAAGATAGAGTTGTAAGtggTTTCATTGA	
SST2_FWD	TGACTAGGACTTGGATTTGGTTGC	
SST2_RVS	GCGCTCACGTTAGTCACATCTC	
sst2.3_C	GCTATTTCTAGCTCTAAAACgtcagacgtatacaaagatgCTGCCAATCGCAGCTCCCAG	
sst2.3_W	CTGGGAGCTGCGATTGGCAGcatctttgtatacgtctgacGTTTTAGAGCTAGAAATAGC	
sst2.5_C	GCTATTTCTAGCTCTAAAACatttttatccaccatcttacGATCATTTATCTTTCACTGC	
sst2.5_W	GCAGTGAAAGATAAATGATCgtaagatggtggataaaaatGTTTTAGAGCTAGAAATAGC	
STE12_FWD	ACTCTTCGCGGTCAGGTCTC	CRISPR

STE12_RVS	GGCAATACTACGTTGGTATCAAAATAGTGG	deletion of STE12 gene and verification
STE12.3_C	gctatttctagctctaaaactcgattggtatctacctcaaCTGCCAATCGCAGCTCCCAG	
STE12.3_W	CTGGGAGCTGCGATTGGCAGttgaggtagataccaatcgagttttagagctagaaatagc	
STE12.5_C	GCTATTTCTAGCTCTAAAACctgttctactattggttattGATCATTTATCTTTCACTGC	
STE12.5_W	GCAGTGAAAGATAAATGATCaataaccaatagtagaacagGTTTTAGAGCTAGAAATAGC	
STE12delta_C	TTTTTAATTCTTGTATCATAAATTCAAAAATTATATTATACCTTGGTGAACAAGACAATTCAAATAAAGAAAGCGGTTC	
STE12delta_W	GGAACCGCTTTCTTTATTTGAATTGTCTTGTTCACCAAGGTATAATATAATTTTTGAATTTATGATACAAGAATTAAAA	
STE2_FWD	TAGGACCTGTGCCTGGCAAG	CRISPR deletion of STE2 gene and verification
STE2_RVS	CATCACAATATACTAGCAGTGGCACC	
STE2.3_C	gctatttctagctctaaaacgaactttctggcttcctcatCTGCCAATCGCAGCTCCCAG	
STE2.3_W	CTGGGAGCTGCGATTGGCAGatgaggaagccagaaagttcgttttagagctagaaatagc	
STE2.5_C	GCTATTTCTAGCTCTAAAACcatcagaCATttttgattctGATCATTTATCTTTCACTGC	
STE2.5_W	GCAGTGAAAGATAAATGATCagaatcaaaaATGtctgatgGTTTTAGAGCTAGAAATAGC	
STE2delta_C	GAAGGTCACGAAATTACTTTTTCAAAGCCGTAAATTTTGATTTTGATTCTTGGATATGGTTCTTAACGGTGCATTTTTA	
STE2delta_W	TTAAAAATGCACCGTTAAGAACCATATCCAAGAATCAAAATCAAAATTTACGGCTTTGAAAAAGTAATTTCGTGACCTT	
STE3_FWD	TGCGTTTCATTTGGCCGTTATCAC	CRISPR deletion of STE3 gene and verification
STE3_RVS	CTTGGTGTGCAGAATAGTGATAGAGC	
STE3.3_C	gctatttctagctctaaaacGCAGTATTTTCTGAACTATGCTGCCAATCGCAGCTCCCAG	
STE3.3_W	CTGGGAGCTGCGATTGGCAGCATAGTTCAGAAAATACTGCgttttagagctagaaatagc	
STE3.5_C	GCTATTTCTAGCTCTAAAACTATTATTGCTGACTTGTATGGATCATTTATCTTTCACTGC	
STE3.5_W	GCAGTGAAAGATAAATGATCCATACAAGTCAGCAATAATAGTTTTAGAGCTAGAAATAGC	
STE3delta_C	AATACTCCTAGTCCAGTAAATATAATGCGACACTCTTGTGGAAAATTTTGATAGTATTTTGCCTTTCCTACACAAATTT	
STE3delta_W	TAAATTTGTGTAGGAAAGGCAAAATACTATCAAAATTTTCCACAAGAGTGTCGCATTATATTTACTGGACTAGGAGTAT	
ScSte2_FWD	ACCAAGAACTTAGTTTCGACGGATACTAGTAAAatgtctgatgcggctccttc	Homology primers for construction of GPCR expression vectors via Gibson Assembly
ScSte2_RVS	ACGAAATTACTTTTTCAAAGCCGTCTCGAGCTAtaaattattattatcttcagtccagaa	
CaSte2_FWD	gtgtcgTCTAGAAAAatgaatatcaattcaactttcatacc	
CaSte2_RVS	gcaagtCTCGAGCtacactcttttgatggtgatttg	
AgSte2_FWD	ACCAAGAACTTAGTTTCGACGGATACTAGTAAAatgggtgaagaggtatctagc	
AgSte2_RVS	ACGAAATTACTTTTTCAAAGCCGTCTCGAGctagttgcaatcacttccggt	
BcSte2_FWD	ACCAAGAACTTAGTTTCGACGGATACTAGTAAAatggcttctaactcttctaacttc	
BcSte2_RVS	ACGAAATTACTTTTTCAAAGCCGTCTCGAGctaagccttttgaacaccgtaag	
CgSte2_FWD	ACCAAGAACTTAGTTTCGACGGATACTAGTAAAatggagatgggctacgatcc	

CgSte2_RVS	ACGAAATTACTTTTTCAAAGCCGTCTCGAGctatttgtcacactgactttgttg	
FgSte2_FWD	ACCAAGAACTTAGTTTCGACGGATACTAGTAAAatgtctaaggaagttttcgacc ca	
FgSte2_RVS	ACGAAATTACTTTTTCAAAGCCGTCTCGAGctacaatggagctctgattctttc	
KlSte2_FWD	ACCAAGAACTTAGTTTCGACGGATACTAGTAAAatgtcagaagagatacccagt ttg	
KlSte2_RVS	ACGAAATTACTTTTTCAAAGCCGTCTCGAGctatcttaattctttgaatacggttttc	
LeSte2_FWD	ACCAAGAACTTAGTTTCGACGGATACTAGTAAAatggacgaagcaatcaatgc aaac	
LeSte2_RVS	ACGAAATTACTTTTTCAAAGCCGTCTCGAGctatttttcaacatagtcacttc	
MoSte2_FWD	ACCAAGAACTTAGTTTCGACGGATACTAGTAAAatggaccaaactttgtctgcta c	
MoSte2_RVS	ACGAAATTACTTTTTCAAAGCCGTCTCGAGctacaatctttcttctcttctttcga	
PbSte2_FWD	ACCAAGAACTTAGTTTCGACGGATACTAGTAAAatggcaccctcattcgacc	
PbSte2_RVS	ACGAAATTACTTTTTCAAAGCCGTCTCGAGctaggcctttgtgccagcttc	
SpSte2_FWD	ACCAAGAACTTAGTTTCGACGGATACTAGTAAAatgagacaaccatggtggaa ag	
SpSte2_RVS	ACGAAATTACTTTTTCAAAGCCGTCTCGAGctacgtccactttttagtttcagattc	
Vp1Ste2_FWD	ACCAAGAACTTAGTTTCGACGGATACTAGTAAAatgagttcccaatcacaccca	
Vp1Ste2_RVS	ACGAAATTACTTTTTCAAAGCCGTCTCGAGctatgaagtccttgtgatatcgttac	
Vp2Ste2_FWD	ACCAAGAACTTAGTTTCGACGGATACTAGTAAAatgtcaggaattgatgatatgg gt	
Vp2Ste2_RVS	ACGAAATTACTTTTTCAAAGCCGTCTCGAGctattgttttctaaatgttattcttttg	
ZbSte2_FWD	ACCAAGAACTTAGTTTCGACGGATACTAGTAAAatgtctggtttggctaacaaca c	
ZbSte2_RVS	ACGAAATTACTTTTTCAAAGCCGTCTCGAGctaccatttgacgttcttcttcaaa	
ZrSte2_FWD	ACCAAGAACTTAGTTTCGACGGATACTAGTAAAatgagtgagattaacaattcta cctac	
ZrSte2_RVS	ACGAAATTACTTTTTCAAAGCCGTCTCGAGctataatttctttaggataattttttact	
SsSte2_FWD	ACACCAAGAACTTAGTTTCGACGGATACTAGTAAAatggatactagtatcaata ctctcaaccct	
SsSte2_RVS	ACGAAATTACTTTTTCAAAGCCGTCTCGAGCTAgctttcagaaaagtgagaggt cgtt	
SjSte2_FWD	ACCAAGAACTTAGTTTCGACGGATACTAGTAAAatgtactcctgggacgaattc	
SjSte2_RVS	ACGAAATTACTTTTTCAAAGCCGTCTCGAGCTAtggcaaagtttcttcggtctt	
ScaSte2_FWD	ACCAAGAACTTAGTTTCGACGGATACTAGTAAAatgtctgacgctccaccac	
ScaSte2_RVS	ACGAAATTACTTTTTCAAAGCCGTCTCGAGCTAttgcttcttgacggtgatctt	
PrSte2_FWD	ACCAAGAACTTAGTTTCGACGGATACTAGTAAAatggcttctatggttccacca	
PrSte2_RVS	ACGAAATTACTTTTTCAAAGCCGTCTCGAGCTAgacgatggagttgttacgttg	
MgSte2_FWD	ACCAAGAACTTAGTTTCGACGGATACTAGTAAAatggtggtaacagctccacct	
MgSte2_RVS	ACGAAATTACTTTTTCAAAGCCGTCTCGAGCTAgtcggaacggactgagtatg	
CguSte2_FWD	ACCAAGAACTTAGTTTCGACGGATACTAGTAAAatgaagtcctgctccatcgg	
CguSte2_RVS	ACGAAATTACTTTTTCAAAGCCGTCTCGAGCTAgatggaggtggagtcgatca	
CtSte2_FWD	ACCAAGAACTTAGTTTCGACGGATACTAGTAAAatggacatcaacaacaccatc	
CtSte2_RVS	ACGAAATTACTTTTTCAAAGCCGTCTCGAGCTAgaccttcttgtaggtgactt	
CpSte2_FWD	ACCAAGAACTTAGTTTCGACGGATACTAGTAAAatgaacaagattgtctccaagt t	
CpSte2_RVS	ACGAAATTACTTTTTCAAAGCCGTCTCGAGCTAttggttgttgtgagcggtct	
SoSte2_FWD	ACCAAGAACTTAGTTTCGACGGATACTAGTAAAatgcgtgaaccatggtggaag	

SoSte2_RVS	ACGAAATTACTTTTTCAAAGCCGTCTCGAGCTAtggccacttcttgatttcggt	
SnSte2_FWD	ACCAAGAACTTAGTTTCGACGGATACTAGTAAAatggcttctatggttccacca	
SnSte2_RVS	ACGAAATTACTTTTTCAAAGCCGTCTCGAGCTAacctctttcaccgacttcac	
CcSte2_FWD	ACCAAGAACTTAGTTTCGACGGATACTAGTAAAatggctgctagaattatccca	
CcSte2_RVS	ACGAAATTACTTTTTCAAAGCCGTCTCGAGCTAgaccatgttttcagaaccaac	
GcSte2_FWD	ACCAAGAACTTAGTTTCGACGGATACTAGTAAAatggccgaagactccatcttc	
GcSte2_RVS	ACGAAATTACTTTTTCAAAGCCGTCTCGAGCTActtacgggtgacgtcggtt	
SkSte2_FWD	ACCAAGAACTTAGTTTCGACGGATACTAGTAAAatgtccggtaagcaagacttg	
SkSte2_RVS	ACGAAATTACTTTTTCAAAGCCGTCTCGAGCTAggtggtcatcaagatcttgga	
AnSte2_FWD	ACCAAGAACTTAGTTTCGACGGATACTAGTAAAatggctacccacaaccaaatc	
AnSte2_RVS	ACGAAATTACTTTTTCAAAGCCGTCTCGAGCTAgacgtcaaaagattcacgacg	
AoSte2_FWD	ACCAAGAACTTAGTTTCGACGGATACTAGTAAAatggactctaagttcgaccca	
AoSte2_RVS	ACGAAATTACTTTTTCAAAGCCGTCTCGAGCTAcaatctttgacaggagtggac	
BbSte2_FWD	ACCAAGAACTTAGTTTCGACGGATACTAGTAAAatggatggttcttctgctcca	
BbSte2_RVS	ACGAAATTACTTTTTCAAAGCCGTCTCGAGCTAggcgaagttatcacgttgcat	
ClSte2_FWD	ACCAAGAACTTAGTTTCGACGGATACTAGTAAAatgaacccagctgacatcaac	
ClSte2_RVS	ACGAAATTACTTTTTCAAAGCCGTCTCGAGAGCTAtcaatctatgggtggtgac	
CnSte2_FWD	ACCAAGAACTTAGTTTCGACGGATACTAGTAAAatggactcctacttgttgaacc	
CnSte2_RVS	ACGAAATTACTTTTTCAAAGCCGTCTCGAGCTActtcataccgatgtcggtgtt	
AfSte2_FWD	ACCAAGAACTTAGTTTCGACGGATACTAGTAAAatgaactccaccttcgaccca	
AfSte2_RVS	ACGAAATTACTTTTTCAAAGCCGTCTCGAGCTAaatatcaccgtgggcgtcctt	
PdSte2_FWD	ACCAAGAACTTAGTTTCGACGGATACTAGTAAAatgtccactgccaacgttcat	
PdSte2_RVS	ACGAAATTACTTTTTCAAAGCCGTCTCGAGCTAgaagatgtcctctctctcgat	
HjSte2_FWD	ACCAAGAACTTAGTTTCGACGGATACTAGTAAAatgtcttccttcgacccatac	
HjSte2_RVS	ACGAAATTACTTTTTCAAAGCCGTCTCGAGCTAagaggaagaagtgttggcgat	
TmSte2_FWD	ACCAAGAACTTAGTTTCGACGGATACTAGTAAAatggagcaaatcccagtctac	
TmSte2_RVS	ACGAAATTACTTTTTCAAAGCCGTCTCGAGCTAggcgaattcgaaacctctttc	
DhSte2_FWD	ACCAAGAACTTAGTTTCGACGGATACTAGTAAAatggaccacaacacccaaca c	
DhSte2_RVS	ACGAAATTACTTTTTCAAAGCCGTCTCGAGCTAgtcatcgtggtcaccaacgt	
SheSte2_FWD	ACCAAGAACTTAGTTTCGACGGATACTAGTAAAatgaaacccgccgctggac	
SheSte2_RVS	ACGAAATTACTTTTTCAAAGCCGTCTCGAGCTAgaccatgtcccttctgacct	
YlSte2_FWD	ACCAAGAACTTAGTTTCGACGGATACTAGTAAAatgcaattgccaccacgtcca	
YlSte2_RVS	ACGAAATTACTTTTTCAAAGCCGTCTCGAGCTAcatcttttcgtcacattcgaaac	
TdSte2_FWD	ACCAAGAACTTAGTTTCGACGGATACTAGTAAAatgtctgactccgcccaaaac	
TdSte2_RVS	ACGAAATTACTTTTTCAAAGCCGTCTCGAGCTAccatttcaaggaggccttacg	
KpSte2_FWD	ACCAAGAACTTAGTTTCGACGGATACTAGTAAAatggaagaatactccgactcc	
KpSte2_RVS	ACGAAATTACTTTTTCAAAGCCGTCTCGAGCTAgaagtgcaaatcttcggaggt	
CauSte2_FWD	ACCAAGAACTTAGTTTCGACGGATACTAGTAAAatggaattcactggtgacat	
CauSte2_RVS	ACGAAATTACTTTTTCAAAGCCGTCTCGAGCTActaaacagttctgttgttcaagtt	
NcSte2_FWD	ACCAAGAACTTAGTTTCGACGGATACTAGTAAAatggcgtcctcttcctcac	
NcSte2_RVS	ACGAAATTACTTTTTCAAAGCCGTCTCGAGCTActcgaatgatctaggcttcgt	
BmSte2_FWD	ACCAAGAACTTAGTTTCGACGGATACTAGTAAAatggcctcaaacggctg	
BmSte2_RVS	ACGAAATTACTTTTTCAAAGCCGTCTCGAGCTAgtcgtcaccgattagtgtatcta	

Ste2_Int_Hom_FWD	GAATTTAAGCAGGCCAACGTCCATACTGCTTAGGACCTGTGCCTGGCAAGTCGCAGATTGAAGagtttatcattatcaatactgc	Integration of GPCRs into Ste2Δ locus
Ste2_Int_Hom_RVS	CTCGTAAAAGCAAAGGTGG	
Ste2_Int_ColPCR_FWD	GTCTCGTGCATTAAGACAGGC	Verification of GPCR integration into Ste2Δ locus
Ste2_Int_ColPCR_RVS	CCTGAGAGTTCTAGATCATGGCAAG	
CaSte2_ColPCR_FWD	TCCAGGATTAGATCAACCAATTC	Determination of strain ratios in mixed culture (Supplementary Figure 21)
CaSte2_ColPCR_RVS	GATTTGAAAGGCAACAACAATC	
KpSte2_ColPCR_FWD	GGACGACTACCACTTCTACGTC	
KpSte2_ColPCR_RVS	AGTATCTGTTCTTCCAGGCGA	
BcSte2_ColPCR_FWD	CTTGATGGCTGACGGTATCA	
BcSte2_ColPCR_RVS	CTCTTGATGTCGTCCAAGTTCTTAC	
Ste3_Int_Hom_FWD	GGTATGGGTGCTAATTTTCGTTAGAAGCGCTGGTACAATTTTCTCTGTCATTGTGACACTA AGTTTATCATTATCAATACTGC	Integration of GPCRs into Ste3Δ locus
Ste3_Int_Hom_RVS	GTAAAAATAAAATACTCCTAGTCCAGTAAATATAATGCGACACTCTTGTGGAAATTACTTTTTCAAAGCCG	
Ste3_Int_ColPCR_FWD	CCTATATTATTGTACCACATTGC	Verification of GPCR integration into Ste3Δ locus
Ste3_Int_ColPCR_RVS	CTGATGAGCTCATCGTTAC	
Ste12*_Int_FWD	CGAAGAAAACACACTTTTATAGCGGAACCGCTTTCTTTATTTGAATTGTCTTGTTCACCAAGGATGGATACTAGTGACTACAAGGACCAC	Replacing the DNA binding domain of Ste12 by ZF43-8 (Ste12*)
Ste12*_Int_RVS	CTTCTTCGTCTCTGCCC	
Ste12*_Int_ColPCR_FWD	CGGAGAGCTCGTTTCAAAATG	Verification of Ste12*
Ste12*_Int_ColPCR_RVS	CTTCTTCGTCTCTGCCC	
CYC1t_Int_Hom_FWD	GTAGACATACTGTATATACACGAGGGCGTATCGTTCACCAGAAAGAATATAAACATAACAAGATAAACATGTAATTAGTTATGTCAC	Replacing Sec4 promoter with CYC1t-OSR2
CYC1t_Int_Hom_RVS	GAGTCCTCACTCTATTAATATTTTCGAGTCCTCACTCTGTCGACCTCGAGGGGGGGCCCGGTACCCAATTCGCCGGCCGCAAATTAAAGC	
OSR2_Int_Hom_FWD	GTACGCATGTAACATTATACTGAAAACCTTGCTTGAGAAGGTTTTGGGACGCTCGAAGGCTTTAATTTGCGGCCGGCGAATTGGGTACC	
OSR2_Int_Hom_RVS	GAGTCATAGCTCTTTCCATTACCTGAGGACGCTGAGACAGTTCTCAAGCCTGACATTTTTTATCTAGATTAGTGTGTGTATTTGTGTTTG	
OSR4_Int_Hom_FWD	CGAGAGATTTGCAAAGGGTCTCGACGTCAACAAATACACGTCGAAAGAAAGACAAAAGTTATCCAAAACGGATggcgaattgggtac	Replacing Sec4 promoter with OSR4
OSR4_Int_Hom_RVS	ATAAAATTTTCATAATAGAGTCATAGCTCTTTCCATTACCGGATGAAGCAGAAACAGTTCTCAAGCCTGACATCTAGATTTTTTCGATGC	
Sec4_Int_ColPCR_FWD	GGAATTTGTTGTCAGC	Verification of Sec4 promoter replacement with OSRs
Sec4_Int_ColPCR_RVS	GATACCCATAGCACCAC	

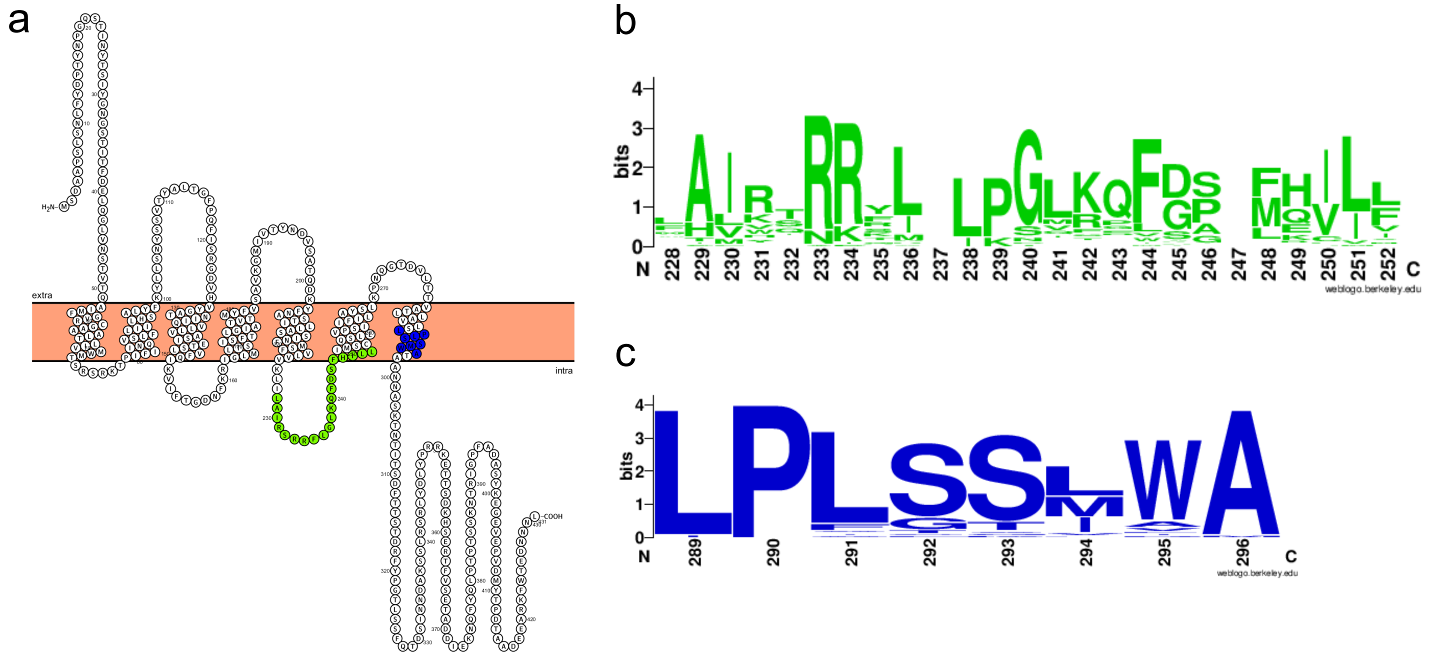

Supplementary Figure 1. Conserved motifs reported to be important for signaling. a: Detailed view of the receptor topology with seven transmembrane helixes. Key regions involved in signaling are highlighted in green and blue. **b and c:** Residue conservation among the herein reported fungal GPCRs for the regions highlighted in green and blue in panel a. Sequence logos were generated using multiple sequence alignments generated with Clustal Omega[13] and using the WebLogo online tool.[14] Numbering refers to the amino acid residue in the *S. cerevisiae* Ste2.

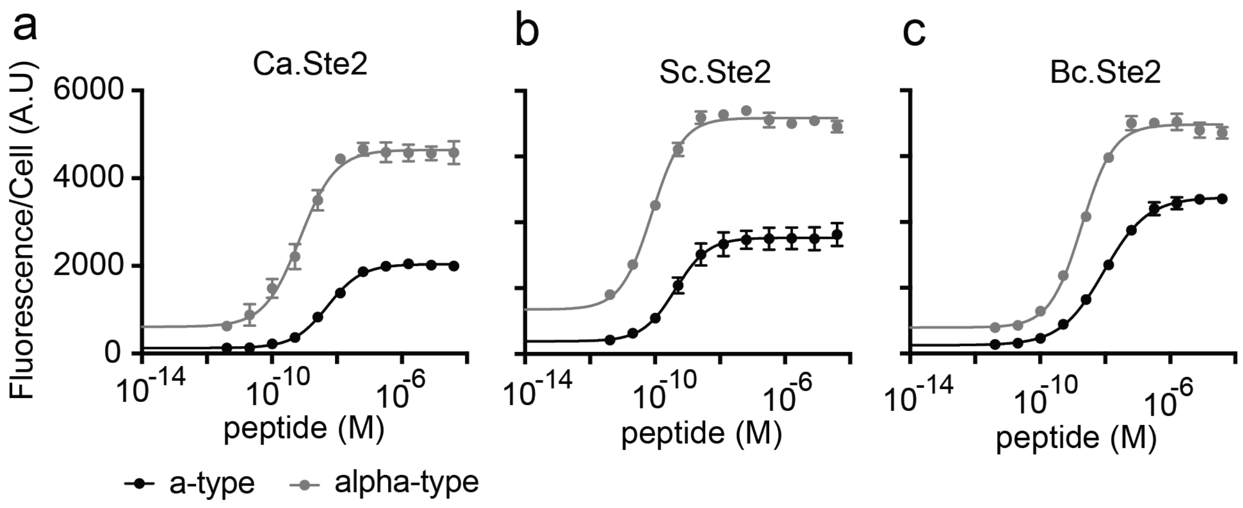

Supplementary Figure 2. Verification of the peptide/GPCR language in a- and alpha-mating types. Functionality of three peptide/GPCR pairs was verified in both mating-types. **a:** Ca.Ste2; **b:** Sc.Ste2; **c:** Bc.Ste2**.** Strain yNA899 (a-type) and yNA903 (alpha-type) were transformed with the appropriate GPCR expression constructs as well as with a plasmid encoding for a *FUS1p*-controlled red fluorescent read-out. Dose response of both strains to the appropriate synthetic peptide is shown. Fluorescence was recorded after 12 hours of incubation. Experiments were run in triplicates and error bars represent the standard deviation.

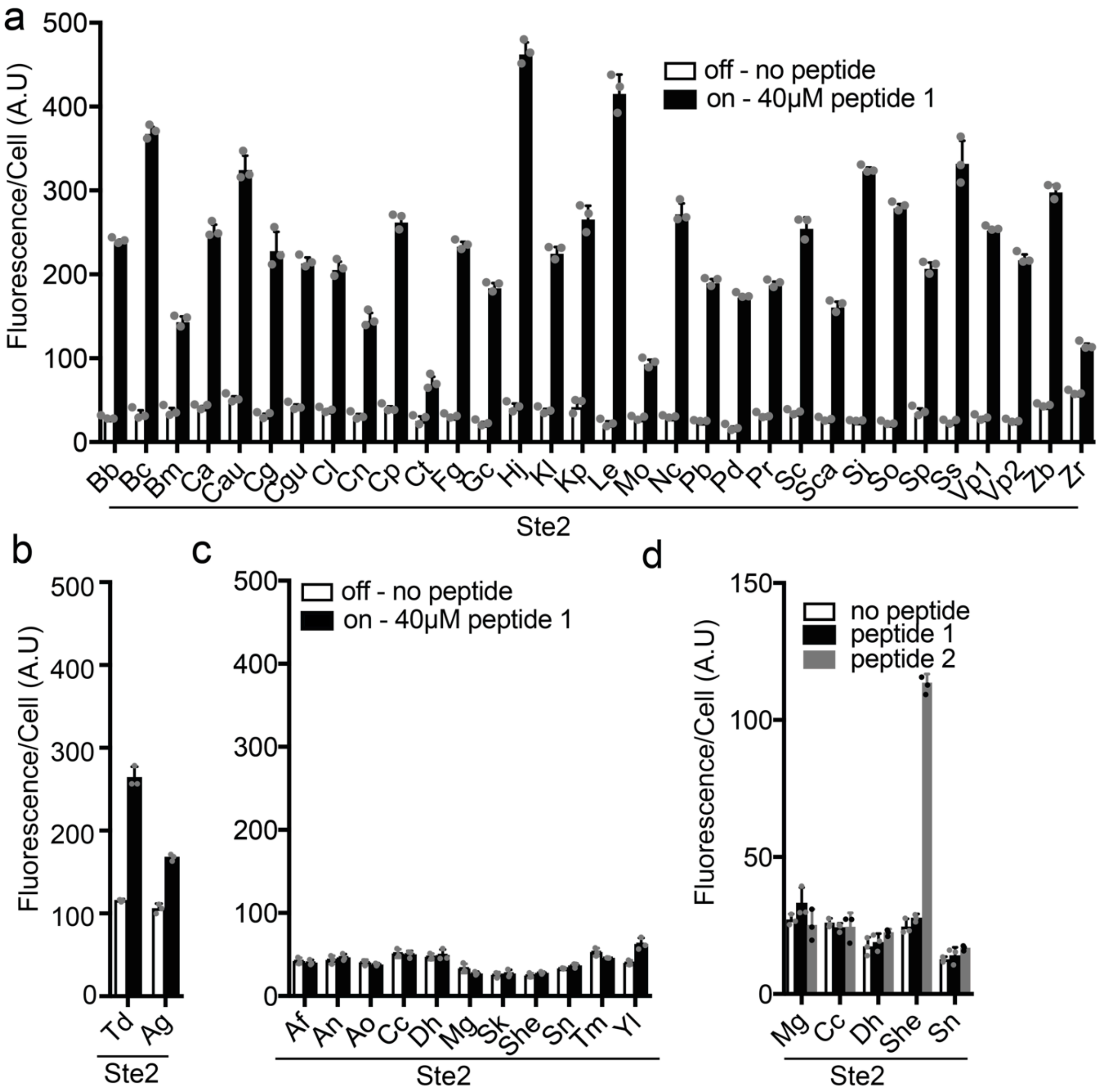

Supplementary Figure 3. Basal and maximal activation levels of all functional, constitutive and non-functional peptide/GPCR pairs. JTy014 was transformed with the appropriate GPCR expression construct. Cells were cultured in the absence or presence of 40 µM cognate synthetic peptide ligand. The peptide sequence #1 (**Supplementary Table 1, Supplementary Table 2**) was used for each GPCR. OD_{600} and Fluorescence was recorded after 8 hours. Experiments were performed in 96-well plates (200 µl total culture volume), run in triplicates, and error bars indicate the standard deviation. **a:** Functional peptide/GPCR pairs. **b:** constitute GPCRs and their additional activation by cognate peptide ligand. **c:** Non-functional peptide/GPCR pairs. **d:** Activation of non-functional GPCRs by alternative peptide ligands (**Supplementary Table 1, Supplementary Table 2**).

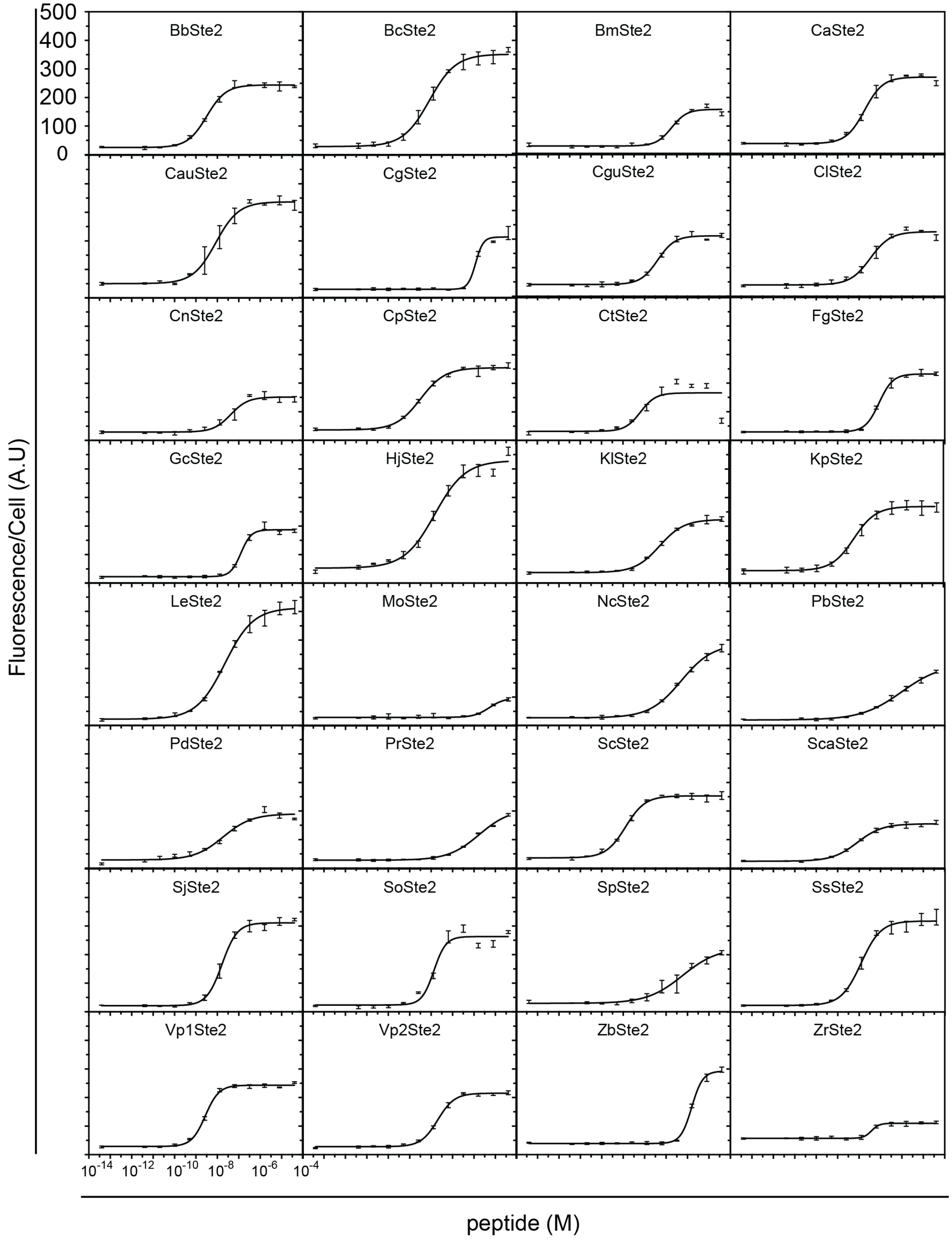

Supplementary Figure 4. Dose response curves for all functional peptide/GPCR pairs. Strain JTy014 was transformed with the appropriate GPCR expression constructs. Each strain was tested with its cognate synthetic peptide. GPCR activation was monitored by activation of a red fluorescent reporter gene under the control of the *FUS1* promoter. Data were collected after 8 hours. Experiments were run in triplicates, and error bars represent the standard deviation.

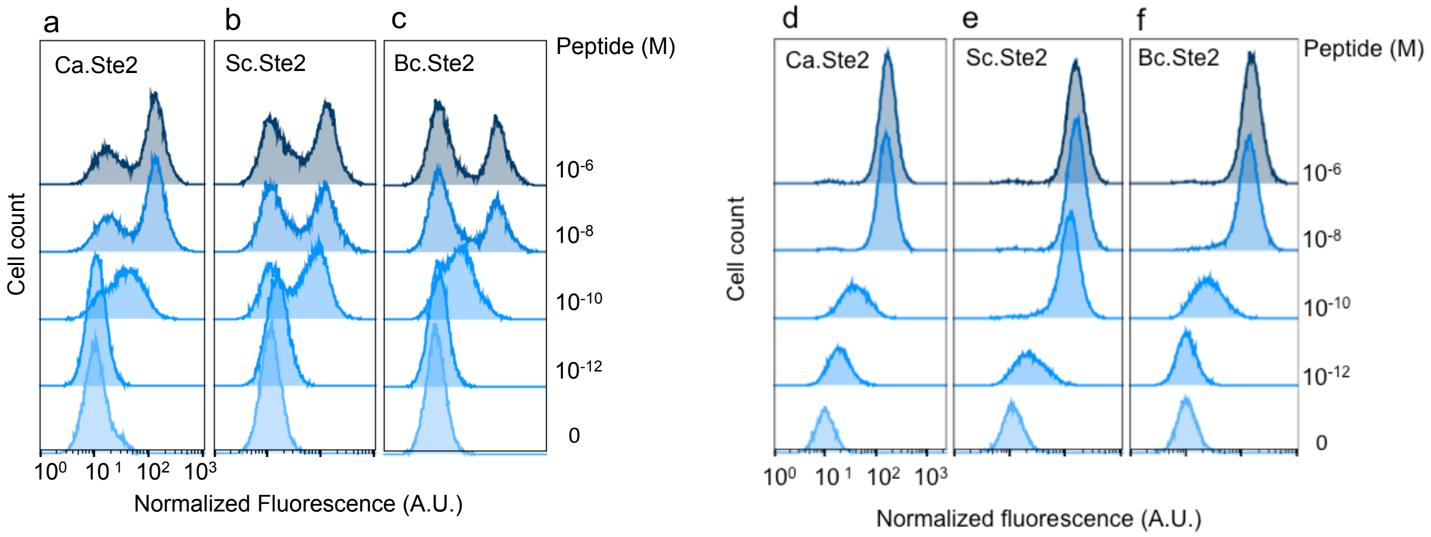

Supplementary Figure 5. GPCR response behavior on single cell level when expressed from plasmids or when integrated into the chromosome (Ste2 locus). Flow cytometry was used to investigate the response behavior for three GPCRs on single cell level when exposed to increasing concentrations of their corresponding peptide ligand. **a-c:** GPCRs are encoded on low copy plasmids and the fluorescent read-out is integrated on the chromosome (HO locus). **a:** JTy014 with pMJ90 (Ca.Ste2). **b:** JTy014 with pMJ93 (Sc.Ste2). **c:** JTy014 with pMJ95 (Bc.Ste2). **d-f:** Both, GPCRs and the red fluorescent readout are integrated on the chromosome. **d**: ySB98 with chromosomally integrated Ca.Ste2. **e:** ySB99 with chromosomally integrated Sc.Ste2. **f:** ySB100 with chromosomally integrated Bc.Ste2. For each sample 50,000 cells were analysed using a BD LSRII flow cytometer (excitation: 594nm, emission: 620nm). The fluorescence values were normalized by the forward scatter of each event to account for different cell size using FlowJo Software. Data of a single experiment are shown, but data were reproduced several times.

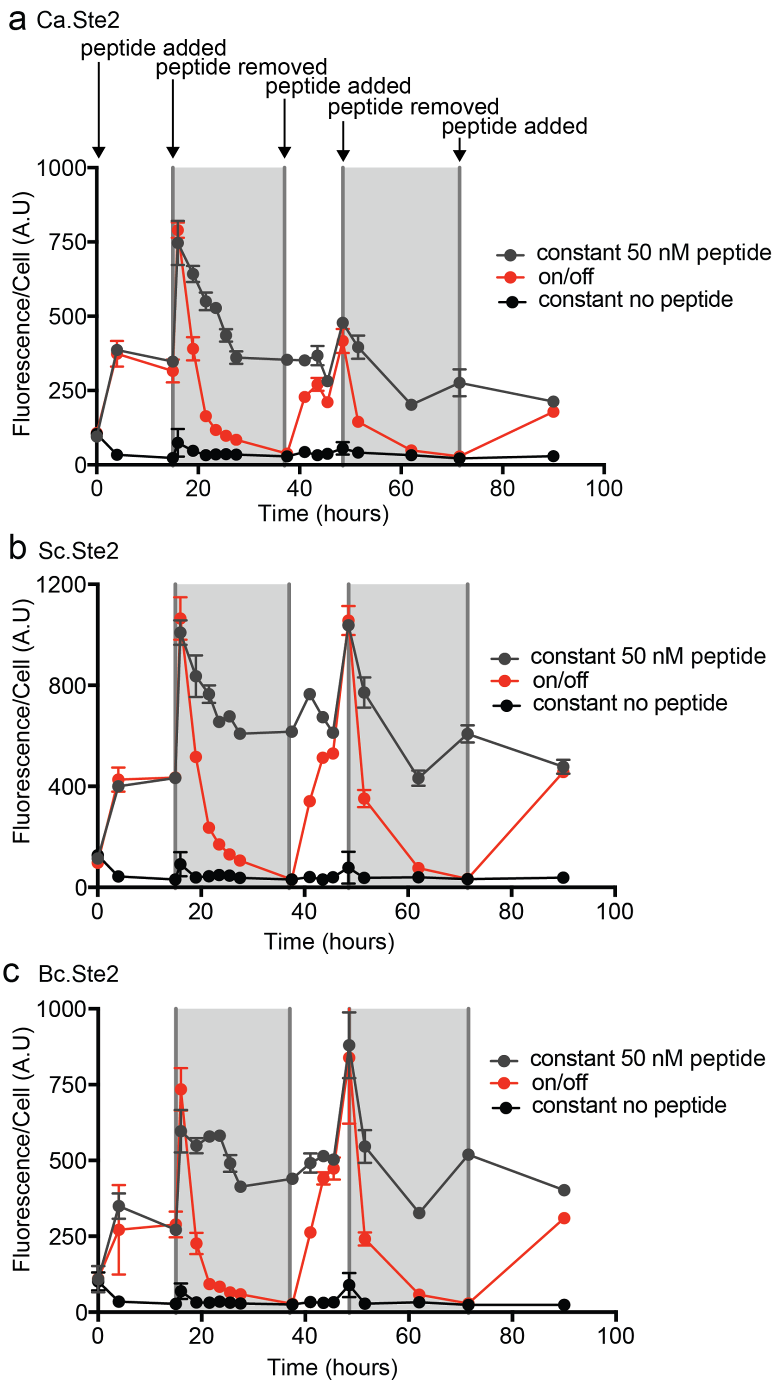

Supplementary Figure 6. Reversibility and re-inducibility of GPCR signaling. All strains carry the indicated GPCR and a *FUS1*p-controlled red fluorescent read-out on the chromosome. **a**: ySB98 with chromosomally integrated Ca.Ste2. **b:** ySB99 with chromosomally integrated Sc.Ste2. **c:** ySB100 with chromosomally integrated Bc.Ste2. At time point zero, GPCRs were activated with 50 nM peptide. After reaching sufficient induction, cells were washed with water to remove the peptide. Cells were re-seeded and grown until the fluorescence level went back to baseline. After reaching baseline, cells were re-induced with 50 nM peptide. Positive and negative controls using cells constantly exposed to 50 nM peptide and cells not exposed to peptide were run simultaneously. Experiments were performed in 96-well plates (200 µl total culturing volume) and run in triplicates; error bars indicate the standard deviation.

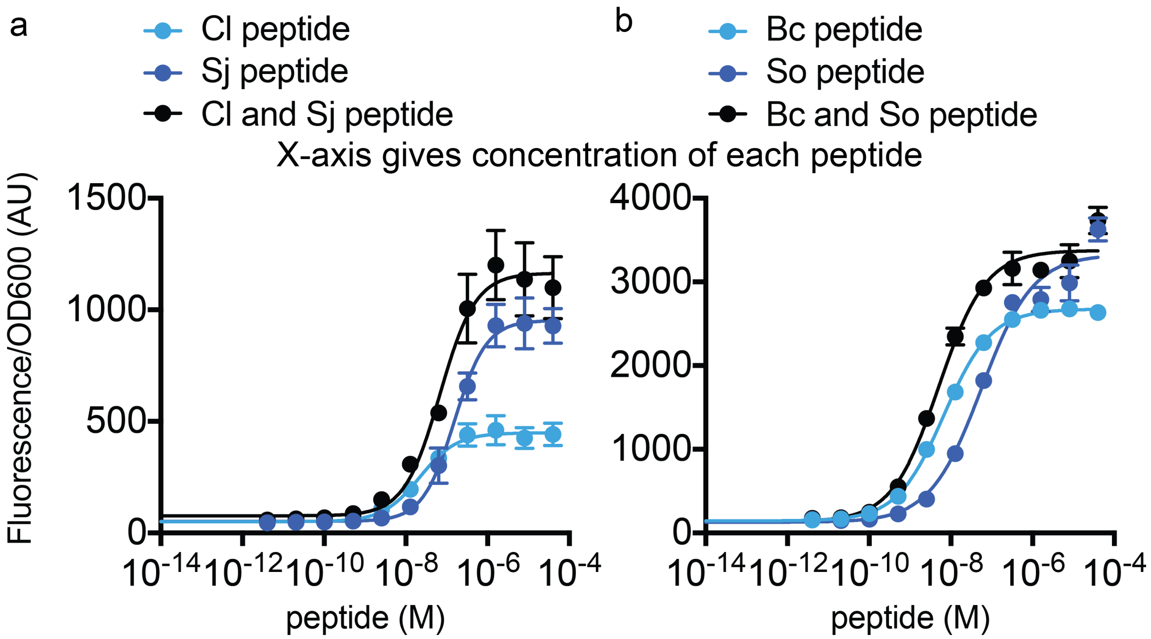

Supplementary Figure 7. Co-expression of two orthogonal GPCRs and single/dual response characteristics. Strain ySB315 (Cl.Ste2 and Sj.Ste2) **(a)** and ySB316 (Bc.Ste2 and So.Ste2) **(b)** were transformed with pSB14 (encoding for a *FUS1* promoter-controlled yEmRFP read out). Each strain was tested with each individual cognate synthetic peptide as well as concurrent activation with both cognate peptides. GPCR activation was monitored by induction of a red fluorescent reporter gene under the control of the *FUS1* promoter. Data were collected after 8 hours. Experiments were run in triplicates; error bars indicate the standard deviation.

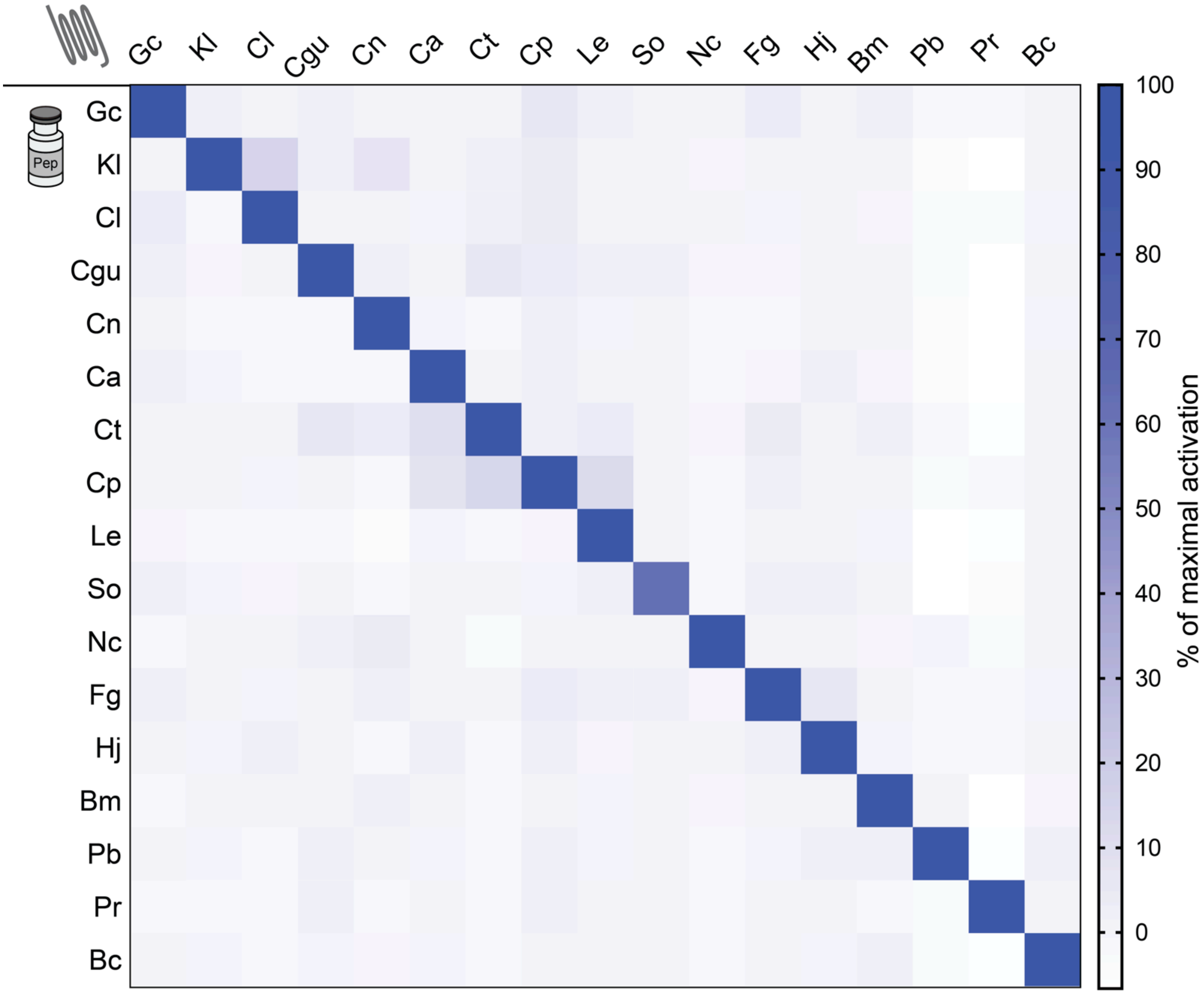

Supplementary Figure 8. 17 receptors are fully orthogonal and not activated by the other 16 non-cognate peptide ligands. Data shown in this Figure were extracted from **Figure 2c**.

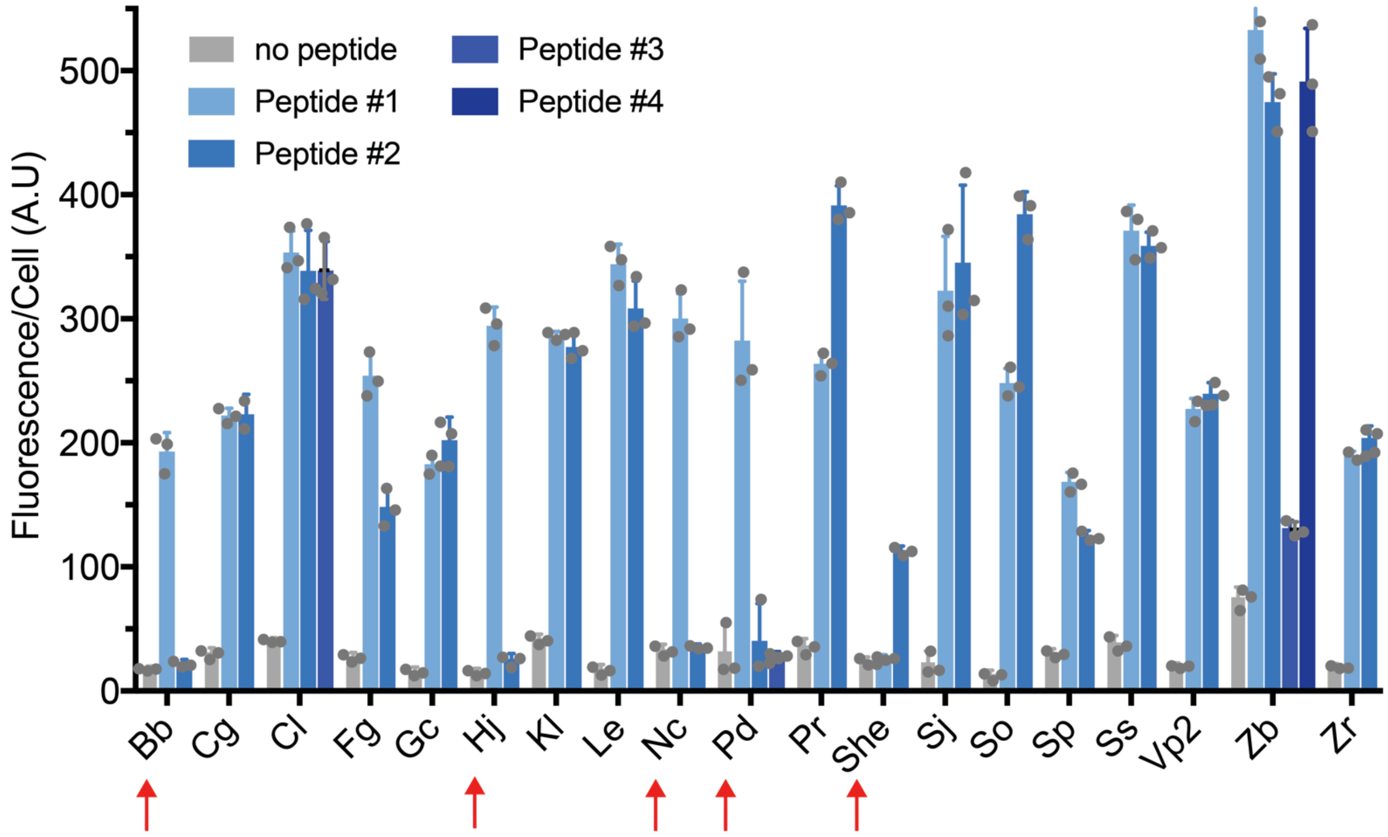

Supplementary Figure 9. On/off screen for 19 GPCRs and their alternative near-cognate peptide ligand candidates. JTy014 was transformed with the appropriate GPCR expression construct. Cells were cultured in the absence or presence of 40 μM synthetic peptide ligand. Numbering of the near-cognate peptide ligand candidates corresponds to **Supplementary Table 2**. OD_{600} and red fluorescence was recorded after 8 hours. Experiments were performed in 96-well plates (200 μl total culture volume), run in triplicates, and error bars indicate standard deviation. Red arrows indicate GPCRs that were not activated by all tested alternative peptide ligand candidates.

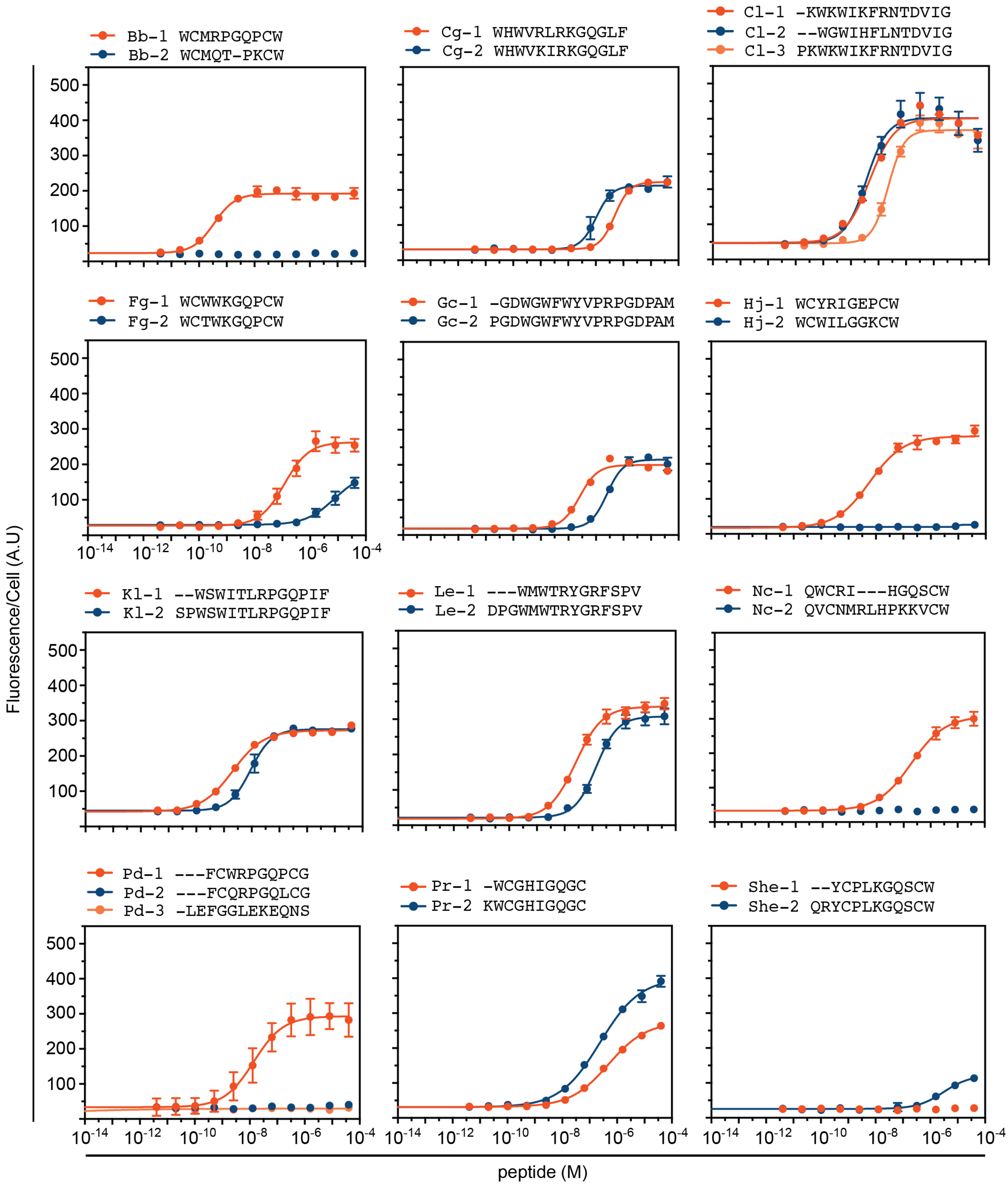

Supp. Figure 10 (continues on next page). Dose response of GPCRs to their alternative near-cognate peptide ligand candidates

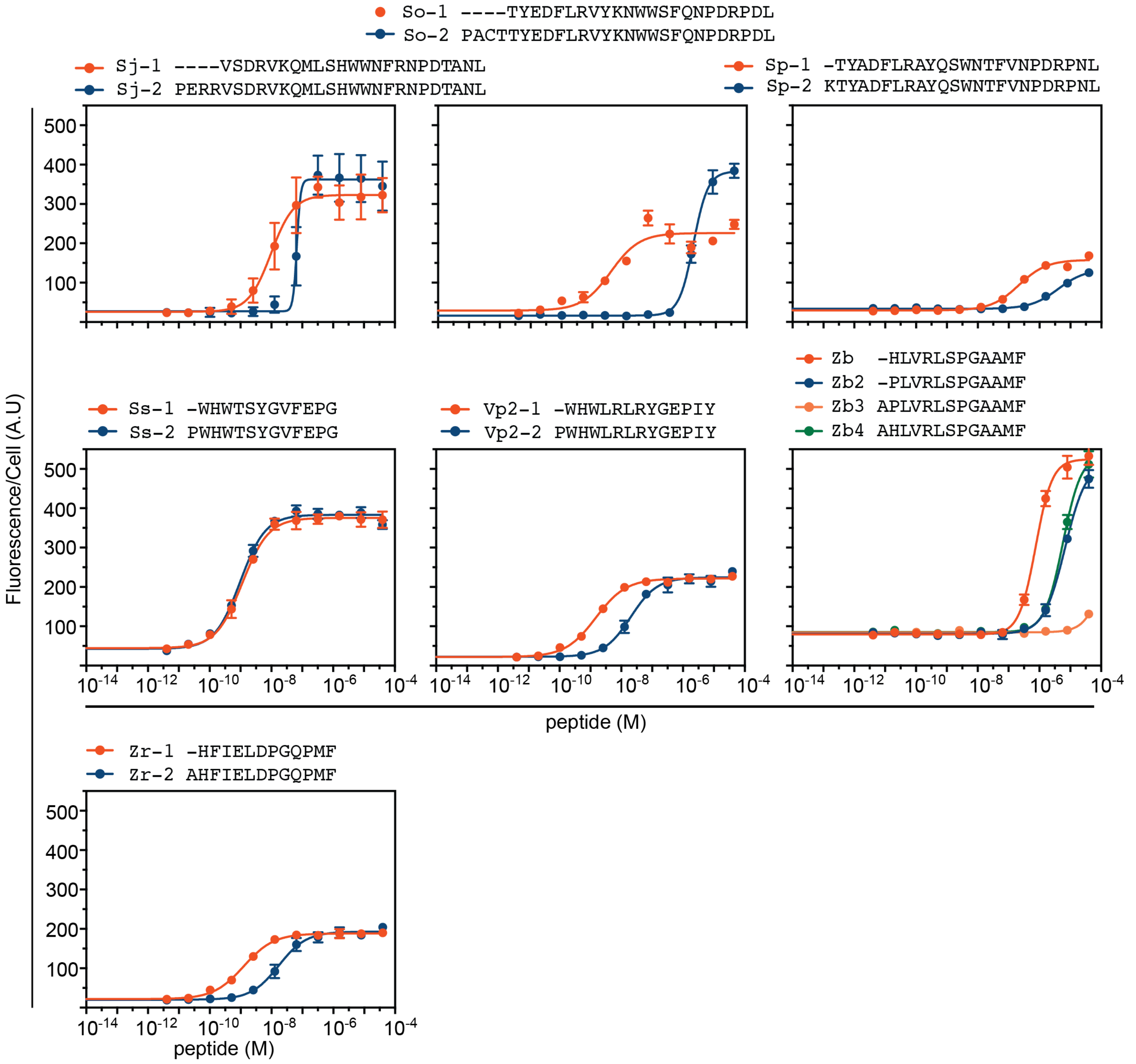

Supplementary Figure 10 (continued). Dose response of GPCRs to their alternative near-cognate peptide ligand candidates. Strain JTy014 was transformed with the appropriate GPCR expression constructs. Each strain was tested with the indicated synthetic peptide ligands. GPCR activation was monitored by activation of a red fluorescent reporter gene under the control of the *FUS1* promoter. Data were collected after 12 hours. Experiments were run in triplicates; error bars indicate standard deviation.

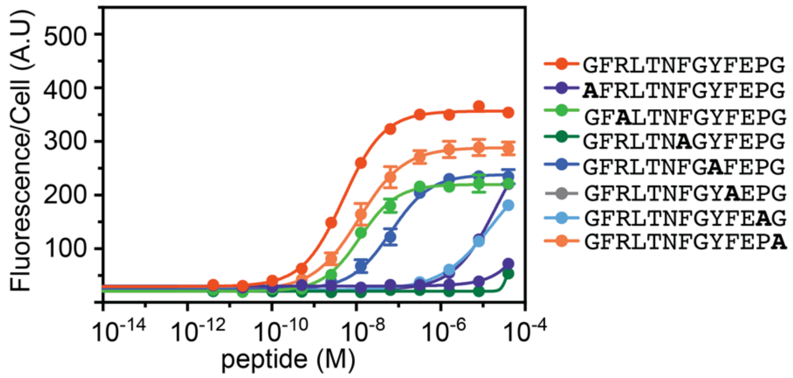

Supplementary Figure 11. Dose response of Ca.Ste2 using alanine-scanned peptide ligands. Strain JTy014 was transformed with the Ca.Ste2 expression construct. The resulting strain was tested with the indicated synthetic peptide ligands. GPCR activation was monitored by activation of a red fluorescent reporter gene under the control of the *FUS1* promoter. Data were collected after 12 hours. Experiments were run in triplicates; error bars indicate standard deviation.

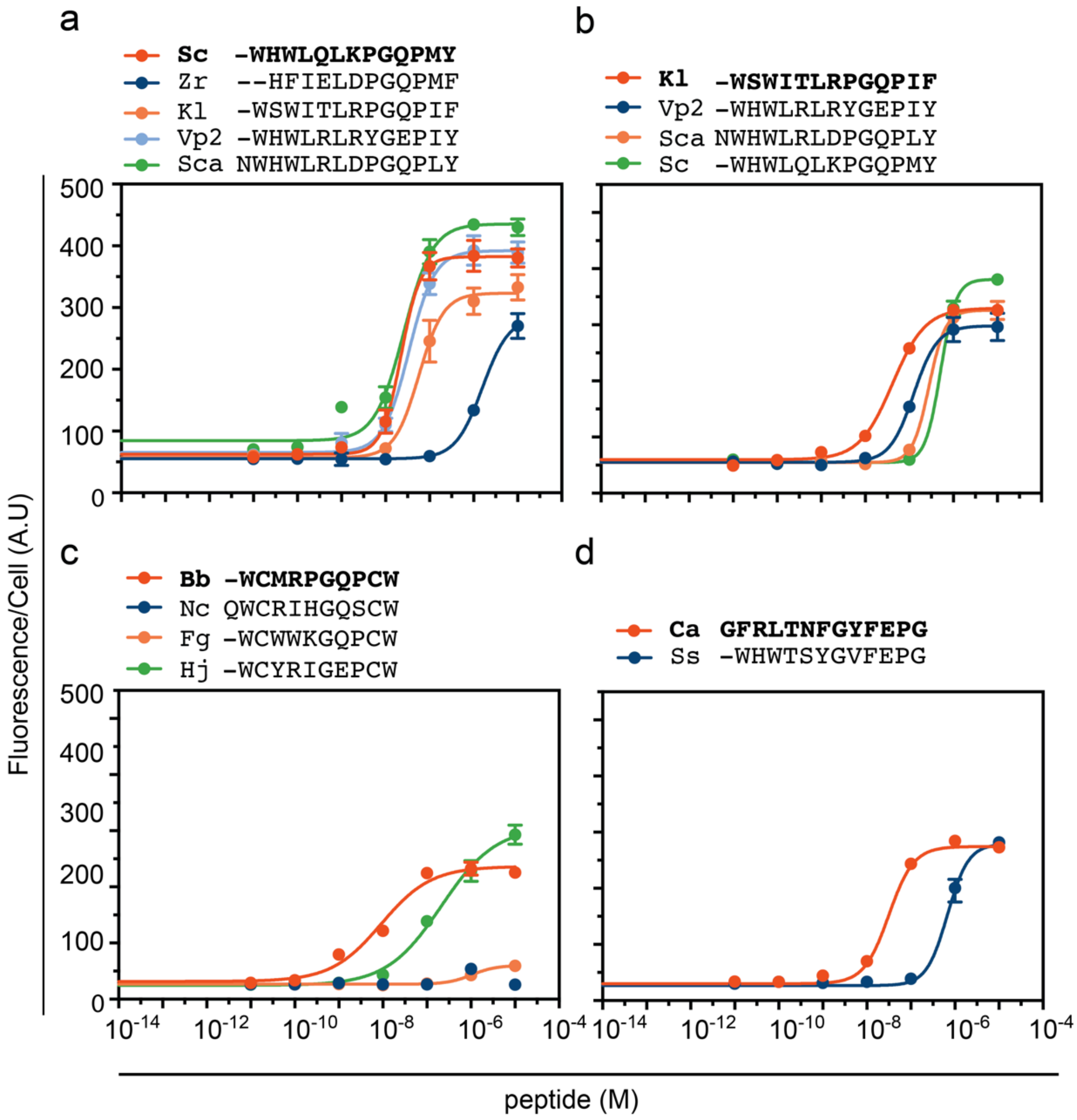

Supplementary Figure 12. Dose responses of promiscuous GPCRs and their cognate or non-cognate peptide ligands. Strain JTy014 was transformed with the appropriate GPCR expression constructs. Each strain was tested with its cognate synthetic peptide ligand #1 and its non-orthogonal non-cognate peptide ligands as indicated. GPCR activation was monitored by activation of a red fluorescent reporter gene under the control of the *FUS1* promoter. Data were collected after 12 hours. Experiments were run in triplicates; error bars indicate standard deviation.

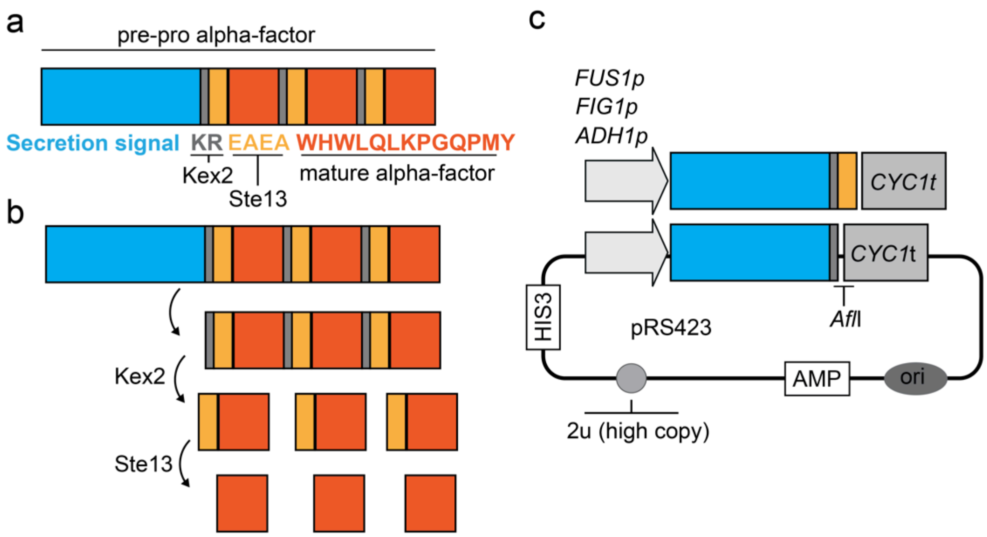

Supplementary Figure 13. Peptide acceptor vector design. a: Schematic representation of the *S. cerevisiae* alpha-factor precursor architecture with the secretion signal (blue), Kex2 (grey) and Ste13 (orange) processing sites and three copies of the peptide sequence (red). **b:** Overview on pre-pro-peptide processing, resulting in mature alpha-factor. **c:** Schematic representation of the peptide acceptor vector. The peptide expression cassette includes either a constitutive promoter (*ADH1p*) or a peptide-dependent promoter (*FUS1p or FIG1p*), the alpha-factor pro sequence with or without the Ste13 processing site, a unique (AflII) restriction site for peptide swapping and a *CYC1* terminator.

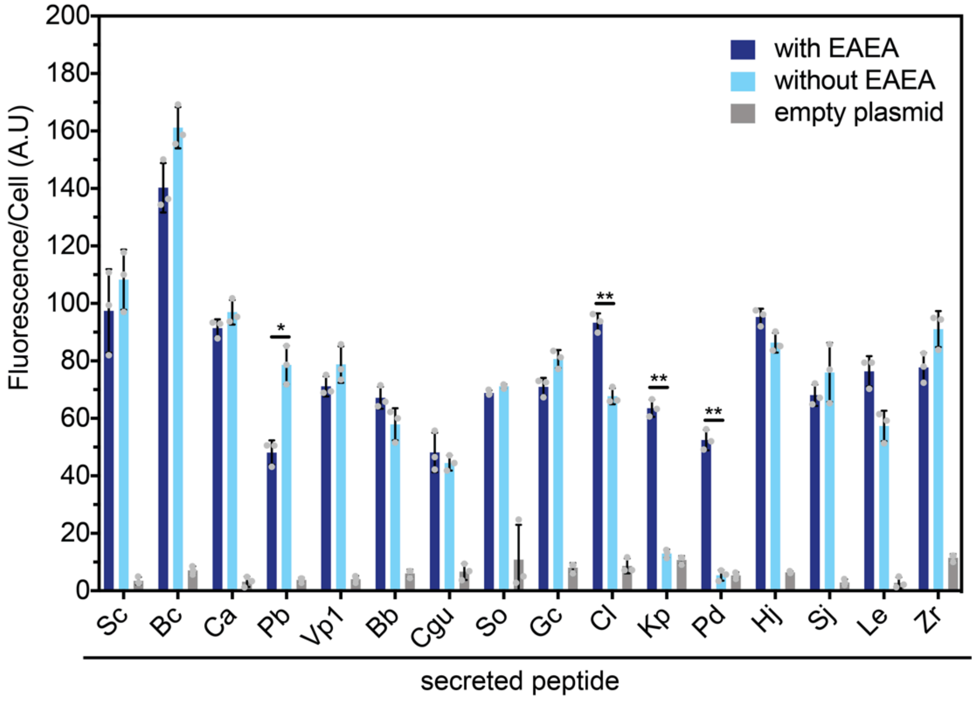

Supplementary Figure 14. Secretion of peptide ligands with and without Ste13 processing site. Peptide expression cassettes with and without the Ste13 processing site (EAEA) were cloned under control of the constitutive *ADH1* promoter. Peptide expression constructs were used to transform strain yNA899 and the resulting strains were co-cultured with a sensing strain expressing the cognate GPCR and a fluorescent read-out. Secretion and Sensing strains were co-cultured 1:1 in 96-well plates (200 µl total culturing volume) and fluorescence was measured after 12 hours. Experiments were run in triplicates; error bars indicate standard deviation. An unpaired t-test was performed for each peptide with an alpha value=0.05. A single asterisk indicates a P value <0.05; a double asterisk indicates a P value <0.01. For simplicity, all peptide constructs eventually used herein contained the Ste13 processing site.

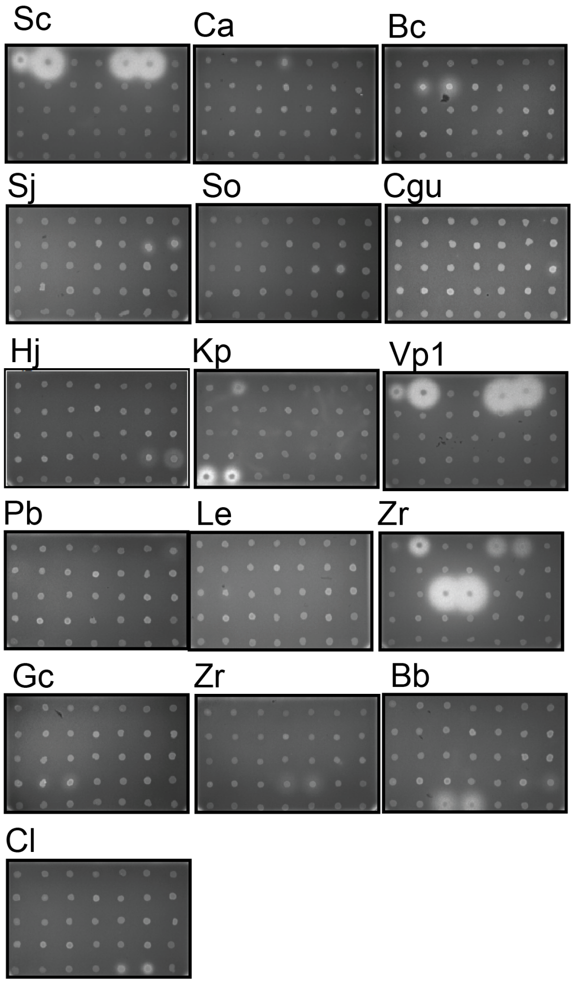

	1	2	3	4	5	6	7
A	Sc	**Sc**	Ca	**Ca**	Vp1	**Vp1**	Pb
B	**Pb**	Bc	**Bc**	BcPb	**PbBc**	Sj	**Sj**
C	Le	**Le**	Zr	**Zr**	So	**So**	Cgu
D	**Cgu**	Gc	**Gc**	Pd	**Pd**	Hj	**Hj**
E	Kp	**Kp**	Bb	**Bb**	Cl	**Cl**	blank

Supplementary Figure 15. Fluorescent halo assay for 16 peptide-secreting strains. Sensing strains for all 16 peptides carrying a pheromone induced red fluorescent reporter, were spread on SC plates. Secreting strains were dotted on the sensing strains in the pattern depicted in scheme bellow. The appearance of a halo around the dot is an indication for secretion of the peptide. All peptides except for Le show a halo. Data of a single experiment are shown.

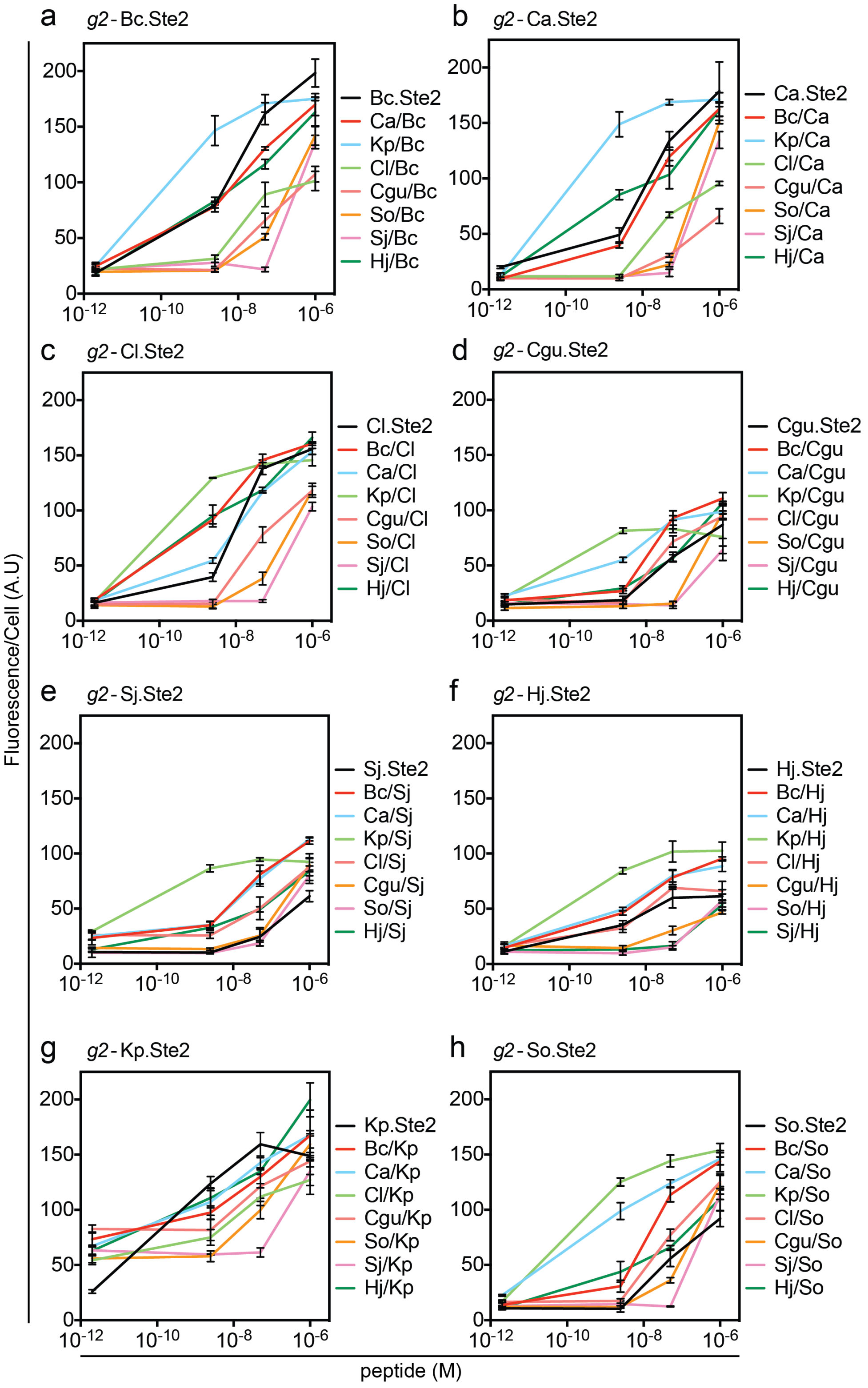

Supplementary Figure 16. Full data set including error bars for Figure 3b. Transfer function strains were co-cultured in a 96-well plate (200 µl total culturing volume) with the appropriate fluorescent reporter strain and experiments were run in triplicate; error bars represent standard deviation. The transfer function strain was induced with synthetic peptide at the following concentrations: 0 µM (H_2O blank), 0.0025 µM, 0.05 µM, 1.0 µM. The black curve for each GPCR represents a control in which the reporter strain was co-cultured with a non-GPCR strain (to maintain the 1:1 strain ratio) and directly induced with the same concentrations of the synthetic peptide.

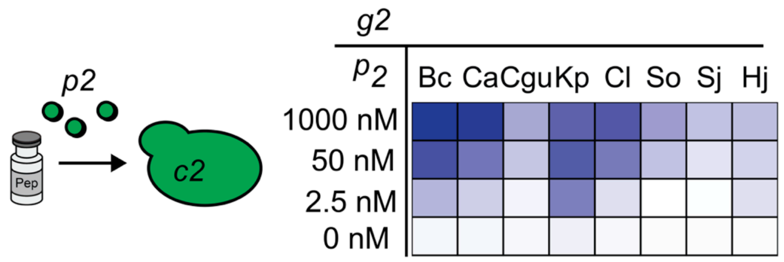

Supplementary Figure 17. Control for Figure 3b. Reference heat maps showing fluorescence values resulting from *c2* being exposed to the indicated doses of synthetic *p2*.

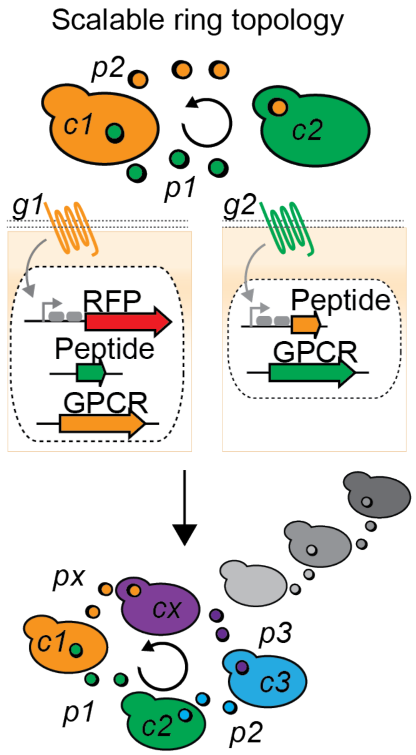

Supplementary Figure 18. Illustration of the scalable communication ring topology. *c1* serves as ring start and closing node. Signaling is started by *c1* secreting *p1* constitutively. Measuring fluorescence read-out in *c1* allows the assessment of functional signal transmission through the ring.

	Yeast 1	Yeast 2	Yeast 3	Yeast 4	Yeast 5	Yeast 6
**Two-Yeast**	ySB98 pFUS1-RFP Ca.Ste2/pADH1-KpPep	yJB416 Kp.Ste2/pFIG1-CaPep				
**Three-Yeast**	ySB98 pFUS1-RFP Ca.Ste2/pADH1-KpPep	yJB416 Kp.Ste2/pFIG1-BcPep	yJB422 Bc.Ste2/pFIG1-CaPep			
**Four-Yeast**	ySB98 pFUS1-RFP Ca.Ste2/pADH1-KpPep	yJB416 Kp.Ste2/pFIG1-BcPep	yJB422 Bc.Ste2/pFIG1-ClPep	yJB418 Cl.Ste2/pFIG1-CaPep		
**Five-Yeast**	ySB98 pFUS1-RFP Ca.Ste2/pADH1-KpPep	yJB416 Kp.Ste2/pFIG1-BcPep	yJB422 Bc.Ste2/pFIG1-ClPep	yJB418 Cl.Ste2/pFIG1-HjPep	yJB523 Hj.Ste2/pFIG1-CaPep	
**Six-Yeast**	ySB98 pFUS1-RFP Ca.Ste2/pADH1-KpPep	yJB416 Kp.Ste2/pFIG1-BcPep	yJB422 Bc.Ste2/pFIG1-ClPep	yJB418 Cl.Ste2/pFIG1-HjPep	yJB523 Hj.Ste2/pFIG1-CguPep	yJB421 Cgu.Ste2/pFIG1-CaPep
**Two-Yeast Dropout**	ySB98 pFUS1-RFP Ca.Ste2/pADH1-KpPep					
**Three-Yeast Dropout**	ySB98 pFUS1-RFP Ca.Ste2/pADH1-KpPep		yJB422 Bc.Ste2/pFIG1-CaPep			
**Four-Yeast Dropout**	ySB98 pFUS1-RFP Ca.Ste2/pADH1-KpPep		yJB422 Bc.Ste2/pFIG1-ClPep	yJB418 Cl.Ste2/pFIG1-CaPep		
**Five-Yeast Dropout**	ySB98 pFUS1-RFP Ca.Ste2/pADH1-KpPep		yJB422 Bc.Ste2/pFIG1-ClPep	yJB418 Cl.Ste2/pFIG1-HjPep	yJB523 Hj.Ste2/pFIG1-CaPep	
**Six-Yeast Dropout**	ySB98 pFUS1-RFP Ca.Ste2/pADH1-KpPep		yJB422 Bc.Ste2/pFIG1-ClPep	yJB418 Cl.Ste2/pFIG1-HjPep	yJB523 Hj.Ste2/pFIG1-CguPep	yJB421 Cgu.Ste2/pFIG1-CaPep

Supplementary Figure 19. Key to the strains used to create the two- to six-yeast paracrine communication rings **(Figure 3d)**. The first linker yeast strain (dropout) was removed to serve as a control for complete signal propagation through the communication ring.

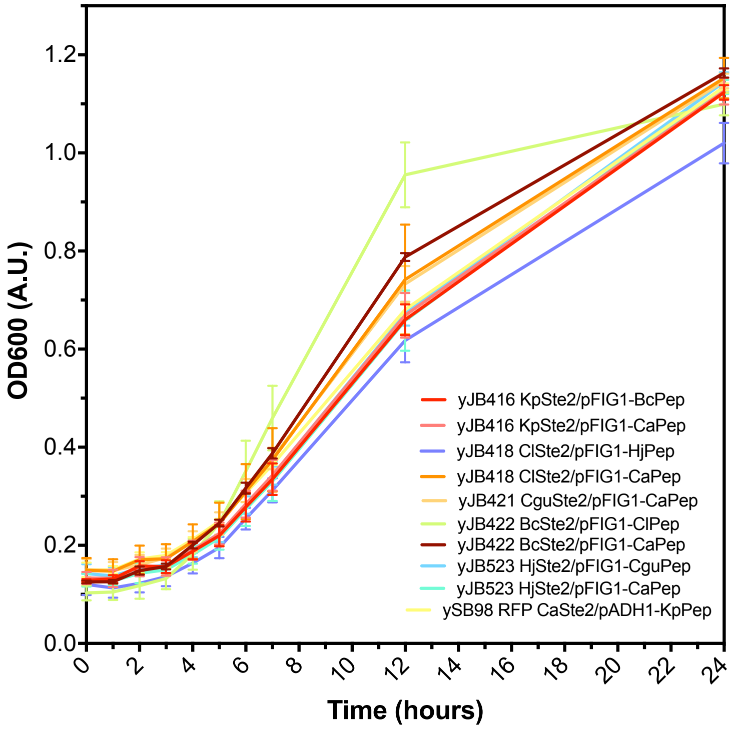

Supplementary Figure 20. Growth of communicating strains. Growth curves of the communication strains were created by seeding each strain in triplicate at OD=0.15 in 200 µL in a 96-well plate and measuring OD_{600} values over 24 hours. Error bars indicate standard deviation. The differential growth phenotypes were partly caused by the expression and secretion burden of specific combinations of GPCRs and peptides. This issue needs to be addressed by optimizing expression and secretion levels. Growth phenotypes were also caused by GPCR-activation (and downstream activation of the mating response) and could be alleviated by using an orthogonal Ste12* that decouples GPCR-activation from the mating response (**Supplementary Figure 24**).

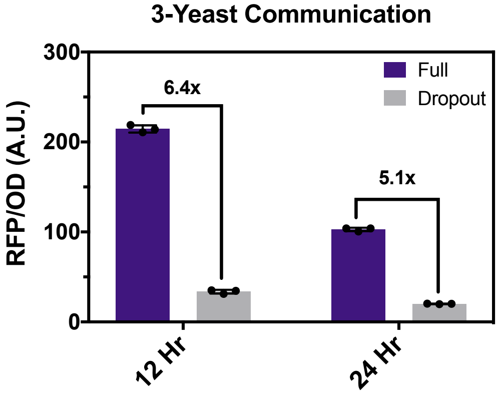

	Percent of Population (%)		
	ySB98 CaSte2 pADH1-KpPep	yJB416 KpSte2 pFIG1-BcPep	yJB422 BcSte2 pFIG1-CaPep
**0 Hr**	37.5	25	37.5
**12 Hr**	52.2	8.7	39.1
**24 Hr**	56.5	4.3	39.1

Supplementary Figure 21. Colony PCR was performed to confirm the presence of co-cultured strains. Samples were taken from a representative three-yeast communication loop and dropout control and plated to get single colonies on selective SD plates. Colony PCR was performed on 24 colonies from each time-point, running three separate PCR reactions in parallel, one for each strain using the integrated GPCR sequence as the strain-specific tag. The three separate PCR reactions were then pooled and visualized on a gel, and bands were counted to determine the ratios of the three communication strains. OD_{600} and red fluorescence measurements were taken in triplicate and processed as for the multi-yeast communication loops. Error bars indicate standard deviation.

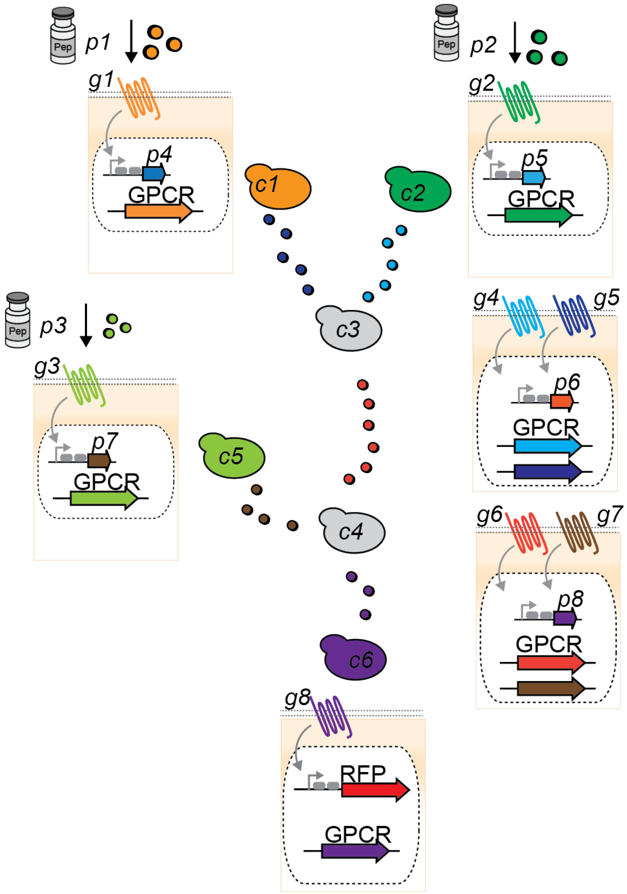

Supplementary Figure 22. Illustration of the six-yeast branched tree-topology (Topology 7, Figure 3c). *c1, c2* and *c5* are induced with synthetic peptides *p1, p2 and p3* to start communication**. Main Figure 3f** features induction with each single peptide, all combinations of two peptides or all three peptides. *c6* serves as closing node. Measuring fluorescence read-out in *c6* allows the assessment of functional signal transmission through the topology. **Topology 6** of **Figure 3c** involves cells *c3*, *c4* and *c6*. **Topology 7** of **Figure 3c** involves cells *c1, c2, c3, c4, c5* and *c6.*

Bus Topology (Figure 3e)

	Peptide	Strain 1	Strain 2	Strain 3
–	Blank	ySB98 pFUS1-RFP Ca.Ste2	ySB315 Cl.Ste2/Sj.Ste2/pFIG1-CaPep	ySB316 BcSte2/SoSte2/pFIG1-ClPep
p1	Bc	ySB98 pFUS1-RFP Ca.Ste2	ySB315 Cl.Ste2/Sj.Ste2/pFIG1-CaPep	ySB316 BcSte2/SoSte2/pFIG1-ClPep
p2	So	ySB98 pFUS1-RFP Ca.Ste2	ySB315 Cl.Ste2/Sj.Ste2/pFIG1-CaPep	ySB316 BcSte2/SoSte2/pFIG1-ClPep
p1+p2	Bc+So	ySB98 pFUS1-RFP Ca.Ste2	ySB315 Cl.Ste2/Sj.Ste2/pFIG1-CaPep	ySB316 BcSte2/SoSte2/pFIG1-ClPep

Tree Topology (Figure 3f)

	Peptide	Strain 1	Strain 2	Strain 3	Strain 4	Strain 5	Strain 6
–	Blank	ySB98 pFUS1-RFP Ca.Ste2	ySB315 Cl.Ste2/Sj.Ste2/pFIG1-CaPep	yJB523 Hj.Ste2/pFIG1-SjPep	ySB316 BcSte2/SoSte2/pFIG1-ClPep	yJB421 Cgu.Ste2/pFIG1-SoPep	yJB416 Kp.Ste2/pFIG1-BcPep
p1	Kp	ySB98 pFUS1-RFP Ca.Ste2	ySB315 Cl.Ste2/Sj.Ste2/pFIG1-CaPep	yJB523 Hj.Ste2/pFIG1-SjPep	ySB316 BcSte2/SoSte2/pFIG1-ClPep	yJB421 Cgu.Ste2/pFIG1-SoPep	yJB416 Kp.Ste2/pFIG1-BcPep
p2	Cgu	ySB98 pFUS1-RFP Ca.Ste2	ySB315 Cl.Ste2/Sj.Ste2/pFIG1-CaPep	yJB523 Hj.Ste2/pFIG1-SjPep	ySB316 BcSte2/SoSte2/pFIG1-ClPep	yJB421 Cgu.Ste2/pFIG1-SoPep	yJB416 Kp.Ste2/pFIG1-BcPep
p3	Hj	ySB98 pFUS1-RFP Ca.Ste2	ySB315 Cl.Ste2/Sj.Ste2/pFIG1-CaPep	yJB523 Hj.Ste2/pFIG1-SjPep	ySB316 BcSte2/SoSte2/pFIG1-ClPep	yJB421 Cgu.Ste2/pFIG1-SoPep	yJB416 Kp.Ste2/pFIG1-BcPep
p1+p2	Kp+Cgu	ySB98 pFUS1-RFP Ca.Ste2	ySB315 Cl.Ste2/Sj.Ste2/pFIG1-CaPep	yJB523 Hj.Ste2/pFIG1-SjPep	ySB316 BcSte2/SoSte2/pFIG1-ClPep	yJB421 Cgu.Ste2/pFIG1-SoPep	yJB416 Kp.Ste2/pFIG1-BcPep
p1+p3	Kp+Hj	ySB98 pFUS1-RFP Ca.Ste2	ySB315 Cl.Ste2/Sj.Ste2/pFIG1-CaPep	yJB523 Hj.Ste2/pFIG1-SjPep	ySB316 BcSte2/SoSte2/pFIG1-ClPep	yJB421 Cgu.Ste2/pFIG1-SoPep	yJB416 Kp.Ste2/pFIG1-BcPep
p2+p3	Cgu+Hj	ySB98 pFUS1-RFP Ca.Ste2	ySB315 Cl.Ste2/Sj.Ste2/pFIG1-CaPep	yJB523 Hj.Ste2/pFIG1-SjPep	ySB316 BcSte2/SoSte2/pFIG1-ClPep	yJB421 Cgu.Ste2/pFIG1-SoPep	yJB416 Kp.Ste2/pFIG1-BcPep
p1+p2+p3	Kp+Cgu+Hj	ySB98 pFUS1-RFP Ca.Ste2	ySB315 Cl.Ste2/Sj.Ste2/pFIG1-CaPep	yJB523 Hj.Ste2/pFIG1-SjPep	ySB316 BcSte2/SoSte2/pFIG1-ClPep	yJB421 Cgu.Ste2/pFIG1-SoPep	yJB416 Kp.Ste2/pFIG1-BcPep

Supplementary Figure 23. Key to the strains used to create the bus and branched tree topologies **(Figure 3e and f)**.

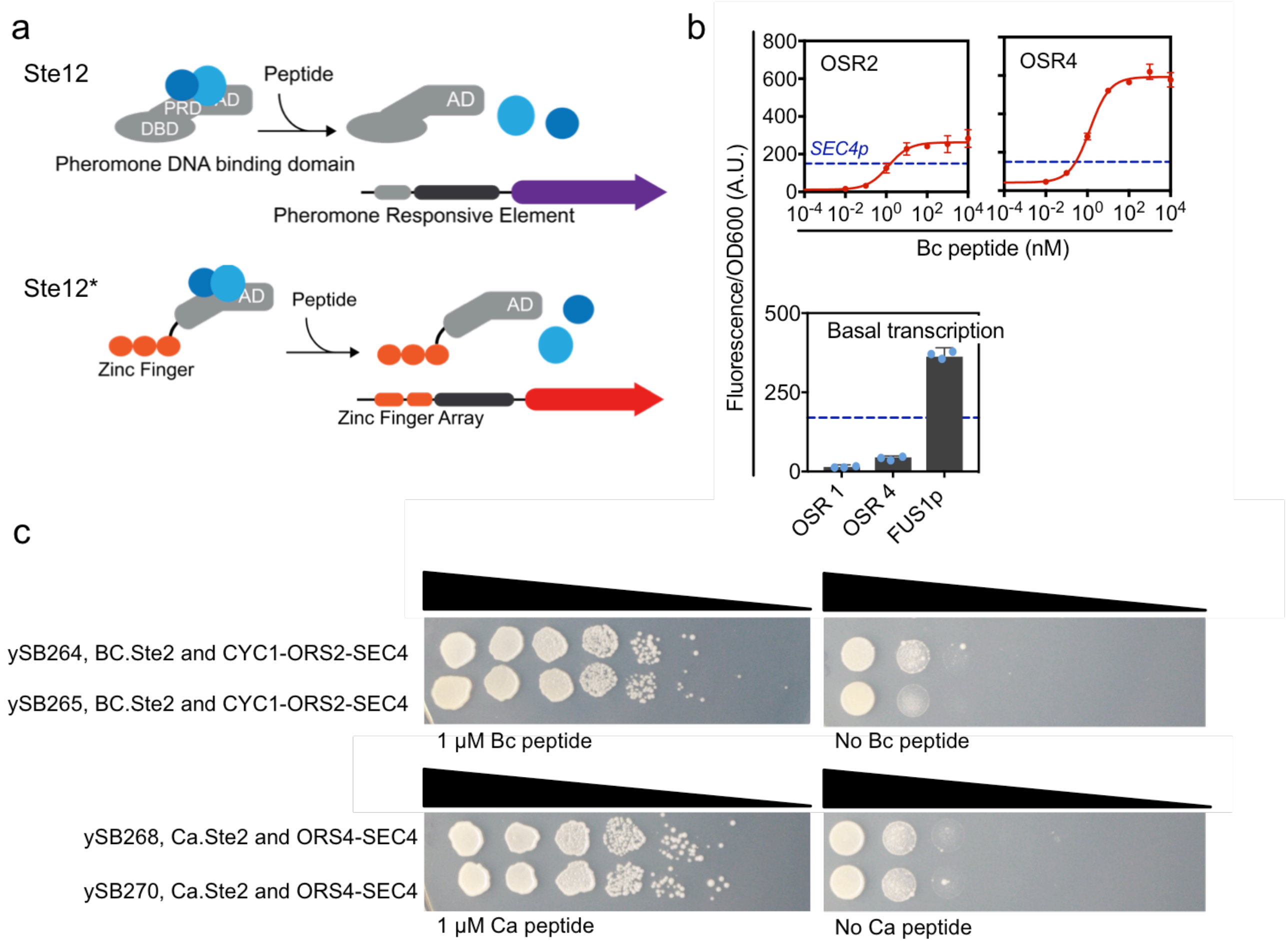

Supplementary Figure 24. Overview on Ste12*, OSR promoters and peptide dependent strains. a. The natural pheromone-inducible transcription factor Ste12 is composed of a DNA binding domain (DBD), a pheromone-responsive domain (PRD) and an activation domain (AD).[15] The orthogonal Ste12* was engineered by replacing the DBD by the zinc-finger-based DNA binding domain 43-8.[16] The Ste12* binds to a zinc-finger responsive element (ZFRE) in a given synthetic promoter. The Ste12* no longer recognizes the natural pheromone response element. **b:** Dose response curves of Bc.Ste2 using a red fluorescent protein driven by OSR2 and OSR4 as read-out. The dotted blue line indicates the expected intracellular levels of Sec4. Levels were estimated by cloning the *SEC4* promoter in front of a red fluorescent read-out and comparing fluorescent/OD values to the OSR promoter read-out. The lower panel highlights the basal transcription levels from the OSR and OSR4 promoters in the absence of plasmid. They are compared to the basal transcription levels of the *FUS1* promoter, which is relatively leaky. Designed orthogonal ste12*-responsive promoters (OSR promoters) feature a core promoter with an 8x repetitive ZFRE upstream of it. OSR2 features a CYC1t core promoter with an integrated upstream repressor element (URS)[17] to reduce basal transcription. OSR4 features the synthetic core promoter 2[18]**. c:** Dot assay of peptide dependent strains ySB268/270 (Ca peptide-dependent strains), ySB188 (Vp1 peptide-dependent strain) and ySB24/265 (Bc peptide-dependent strains) in the presence and absence of peptide. Serial 10-fold dilutions of overnight cultures were spotted on SD agar plates supplemented with or without 1 µM peptide and incubated at 30ºC for 48 hours. Strains ySB264 and ySB268 are individually isolated replicate colonies of strains ySB265 and ySB270.

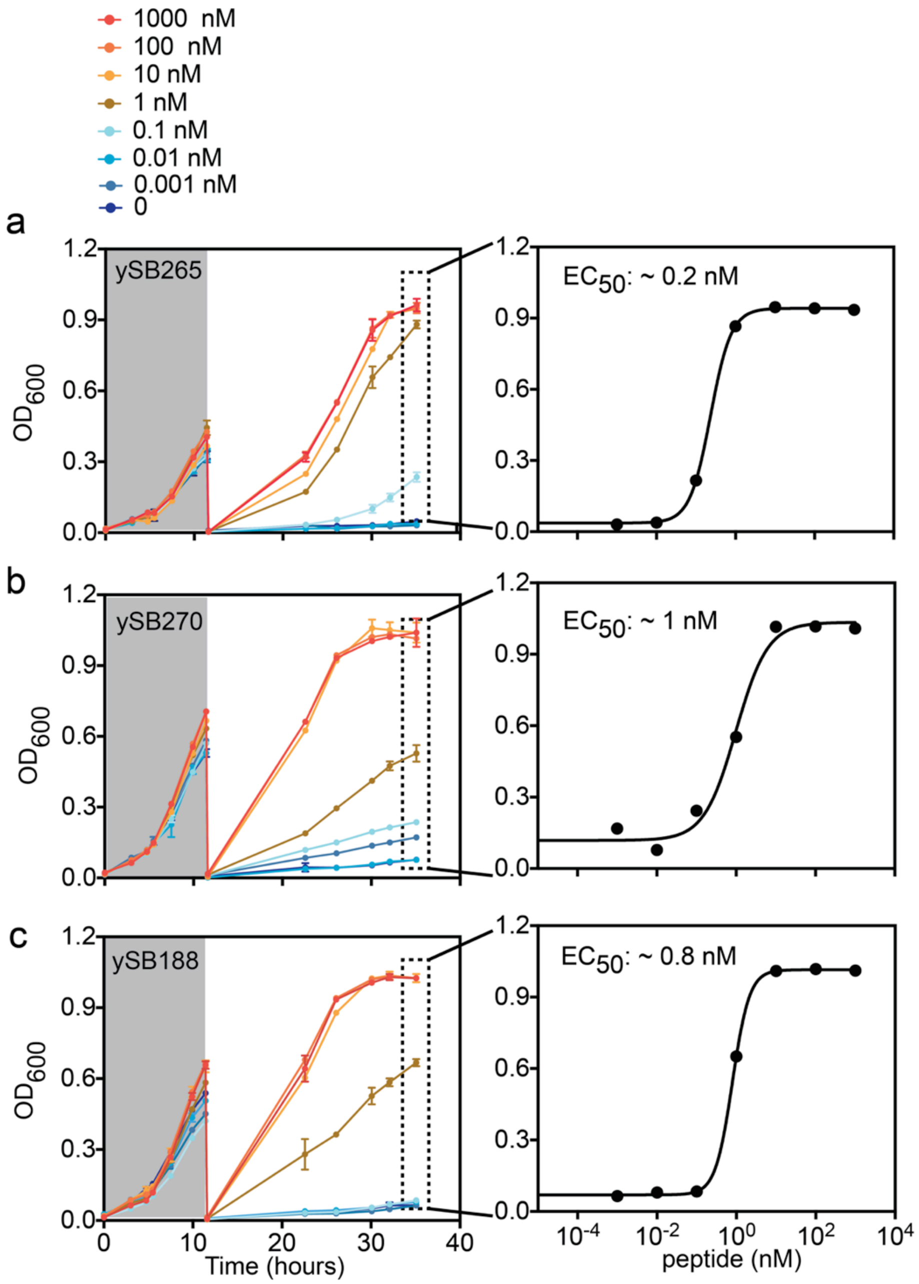

Supplementary Figure 25. EC_{50} of growth for peptide dependent strains. After several doublings the peptide-dependent strains ySB265 (Bc.Ste2) **(a)**, ySB270 (Ca.Ste2) **(b)** and ySB188 (Vp1.Ste2) **(c)** show peptide-concentration dependent growth behavior. The final OD of this experiment (indicated by a dotted box in each panel) was used to calculate the EC_{50} of growth for each strain: OD values were plotted against the log_{10}-converted peptide concentrations peptide concentration and the data were fit to a four-parameter non-linear regression model using Prism (GraphPad). Strains were cultured overnight in the presence of 100 nM peptide in SC(–His). Cells were washed five times with one volumes of water. Cells were than seeded in 200 µl SC (no selection) at an OD_{600} of 0.06 and cultured at 30˚C and 800RPM shaking. Cells were exposed to the indicated concentrations of peptide and OD_{600} was determined at the indicated time points After an initial 12-hour growth, cells were diluted 1:20 into fresh media. Growth was then followed over the course of an additional 24 hours. Growth experiments for each concentration were run in triplicates and errors represent the standard deviation.

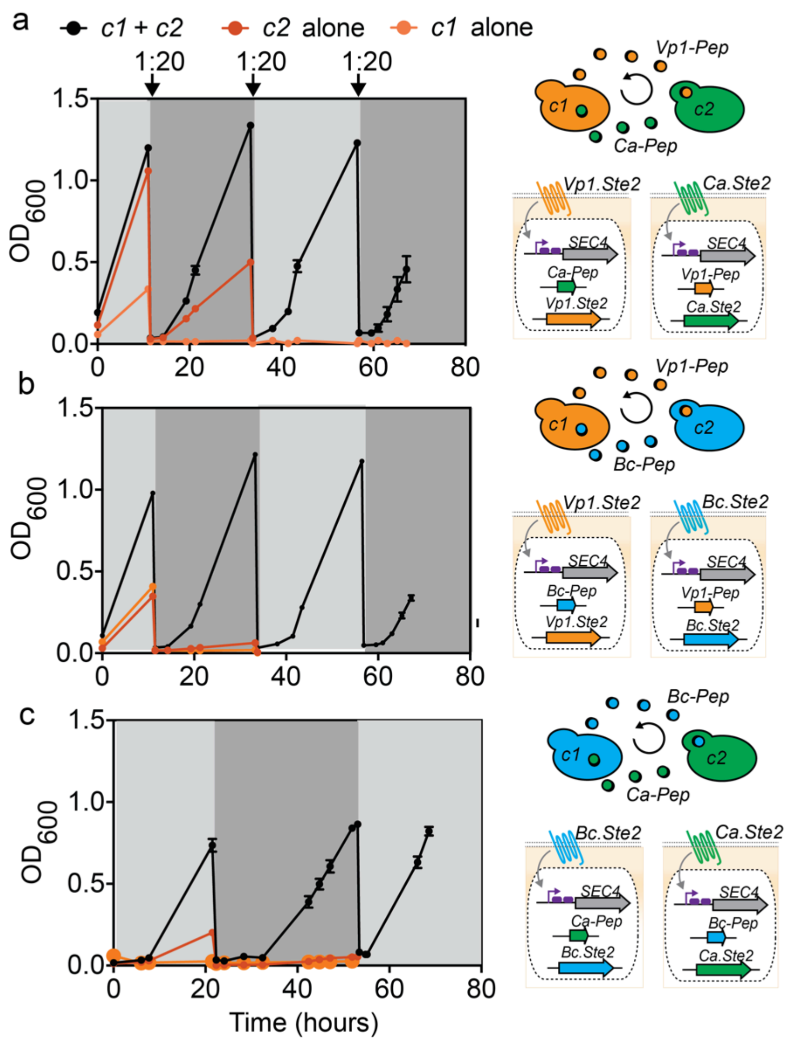

Supplementary Figure 26. Interdependent Two-Yeast links. Strains ySB265 (Bc.Ste2), ySB270 (Ca.Ste2) and ySB188 (Vp1.Ste2) were transformed with the appropriate peptide secretion vectors (Bc, Ca or Vp1) featuring peptide expression under the constitutive *ADH1* promoter. The six resulting strains were used to assemble all three possible two-yeast combinations. The key to the peptide and GPCR combinations is given in the right panels **(a-c)**. The resulting peptide-secreting strains were seeded in the appropriate combination in a 1:1 ratio. The same cell number of single strains was seeded alone and cultured in parallel as control. OD_{600} measurements were taken at the indicated time points and cultures were diluted 1:20 into fresh media at the indicated time points. Co-cultured were maintained for 67 hours. Experiments were run in triplicates and errors represent the standard deviation.

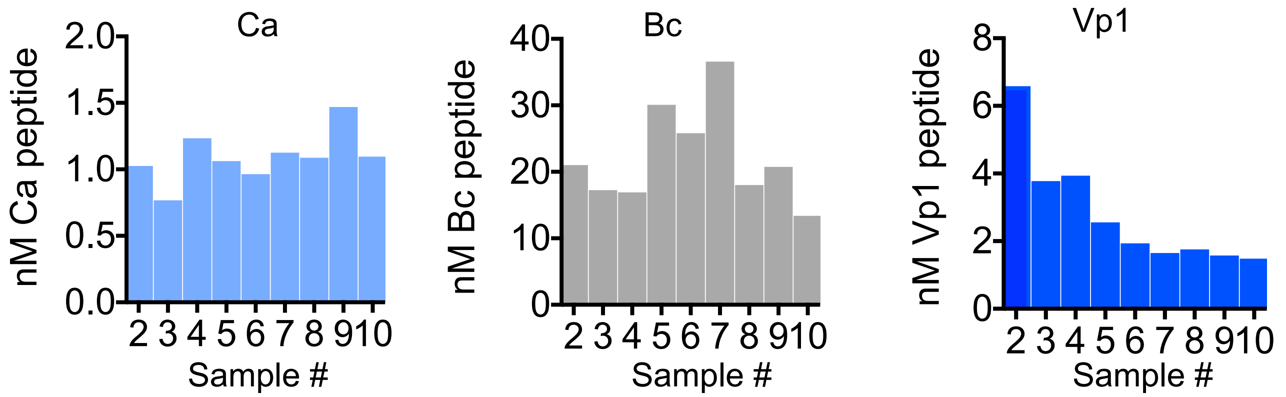

Supplementary Figure 27. Peptide concentrations in the Three-Yeast ecosystem. For one of the three-yeast cultures the peptide concentration in each sample (sample number corresponds to **Figure 2f**) was determined by using the corresponding GPCR/Fluorescent read-out strain (JTy014 expressing Bc, Ca or Vp1.Ste2). **a:** Ca peptide; **b:** Bc peptide; **c:** Vp1 peptide. The linear range of the dose response curve of each GPCR was used for peptide quantification. Note: The Ca peptide could not be accurately quantified as several fluorescent values were out of the linear range. The Y-axis of panel **a** therefore gives approximate amounts. For each sample, a single peptide quantification measurement was performed.

Supplementary References

1. Gibson, D.G. et al. Enzymatic assembly of DNA molecules up to several hundred kilobases. *Nat Methods* **6**, 343-U341 (2009).
2. Kurjan, J. & Herskowitz, I. Structure of a Yeast Pheromone Gene (Mf-Alpha) - a Putative Alpha-Factor Precursor Contains 4 Tandem Copies of Mature Alpha-Factor. *Cell* **30**, 933-943 (1982).
3. Martin, S.H., Wingfield, B.D., Wingfield, M.J. & Steenkamp, E.T. Causes and Consequences of Variability in Peptide Mating Pheromones of Ascomycete Fungi. *Mol Biol Evol* **28**, 1987-2003 (2011).
4. Egelmitani, M. & Hansen, M.T. Nucleotide-Sequence of the Gene Encoding the Saccharomyces-Kluyveri Alpha-Mating Pheromone. *Nucleic Acids Res* **15**, 6303-6303 (1987).
5. Wong, S., Fares, M.A., Zimmermann, W., Butler, G. & Wolfe, K.H. Evidence from comparative genomics for a complete sexual cycle in the 'asexual' pathogenic yeast Candida glabrata. *Genome Biol* **4**, R10 (2003) DOI: 10.1186/gb-2003-4-2-r10.
6. Bennett, R.J., Uhl, M.A., Miller, M.G. & Johnson, A.D. Identification and characterization of a Candida albicans mating pheromone. *Mol Cel Biol* **23**, 8189-8201 (2003).
7. Imai, Y. & Yamamoto, M. The Fission Yeast Mating Pheromone P-Factor Its Molecular-Structure, Gene Structure, and Ability to Induce Gene-Expression and G(1) Arrest in the Mating Partner. *Gene Dev* **8**, 328-338 (1994).
8. Gomes-Rezende, J.A. et al. Functionality of the Paracoccidioides mating alpha-pheromone-receptor system. *PloS one* **7**, e47033 (2012) DOI:10.1371/journal.pone.0047033.
9. Dyer, P.S., Paoletti, M. & Archer, D.B. Genomics reveals sexual secrets of Aspergillus. *Microbiology* **149**, 2301-2303 (2003).
10. Bobrowicz, P., Pawlak, R., Correa, A., Bell-Pedersen, D. & Ebbole, D.J. The Neurospora crassa pheromone precursor genes are regulated by the mating type locus and the circadian clock. *Mol Microbiol* **45**, 795-804 (2002).
11. Ostrov, N. et al. A modular yeast biosensor for low-cost point-of-care pathogen detection. *Science Advances* **3**, e1603221 (2017) DOI: 10.1126/sciadv.1603221.
12. Brachmann, C.B. et al. Designer deletion strains derived from Saccharomyces cerevisiae S288C: a useful set of strains and plasmids for PCR-mediated gene disruption and other applications. *Yeast* **14**, 115-132 (1998).
13. Sievers, F. et al. Fast, scalable generation of high-quality protein multiple sequence alignments using Clustal Omega. *Mol Syst Biol* **7**, 539 (2011) DOI: 10.1038/msb.2011.75.
14. Crooks, G.E., Hon, G., Chandonia, J.M. & Brenner, S.E. WebLogo: A sequence logo generator. *Genome Res* **14**, 1188-1190 (2004).
15. Pi, H.W., Chien, C.T. & Fields, S. Transcriptional activation upon pheromone stimulation mediated by a small domain of Saccharomyces cerevisiae Ste12p. *Mol Cell Biol* **17**, 6410-6418 (1997).
16. Khalil, A.S. et al. A Synthetic Biology Framework for Programming Eukaryotic Transcription Functions. *Cell* **150**, 647-658 (2012).
17. Vidal, M., Brachmann, R.K., Fattaey, A., Harlow, E. & Boeke, J.D. Reverse two-hybrid and one-hybrid systems to detect dissociation of protein-protein and DNA-protein interactions. *Proc Natl Sci USA* **93**, 10315-10320 (1996).
18. Redden, H. & Alper, H.S. The development and characterization of synthetic minimal yeast promoters. *Nat Commun* **6**, 7810 (2015).
